# Impact of Different Screw Designs on Durability of Fracture Fixation: In Vitro Study with Cyclic Loading of Scaphoid Bones

**DOI:** 10.1371/journal.pone.0145949

**Published:** 2016-01-07

**Authors:** Dominik Gruszka, Robert Herr, Hans Hely, Peer Hofmann, Daniela Klitscher, Alexander Hofmann, Pol Maria Rommens

**Affiliations:** 1 Department of Orthopaedics and Traumatology, University Medical Center of Johannes Gutenberg University, Mainz, Germany; 2 Physics Division, RheinMain University of Applied Sciences, Wiesbaden, Germany; University of Sheffield, UNITED KINGDOM

## Abstract

**Purpose:**

The use of new headless compression screws (HCSs) for scaphoid fixation is growing, but the nonunion rate has remained constant. The aim of this study was to compare the stability of fixation resulting from four modern HCSs using a simulated fracture model to determine the optimal screw design(s).

**Methods:**

We tested 40 fresh-frozen cadaver scaphoids treated with the Acumed Acutrak 2 mini (AA), the KLS Martin HBS2 midi (MH), the Stryker TwinFix (ST) and the Synthes HCS 3.0 with a long thread (SH). The bones with simulated fractures and implanted screws were loaded uniaxially into flexion for 2000 cycles with a constant bending moment of 800 Nmm. The angulation of the fracture fragments was measured continuously. Data were assessed statistically using the univariate ANOVA test and linear regression analysis, and the significance level was set at p < 0.05.

**Results:**

The median angulation of bone fragments φ allowed by each screw was 0.89° for AA, 1.12° for ST, 1.44° for SH and 2.36° for MH. With regards to linear regression, the most reliable curve was achieved by MH, with a coefficient of determination of R^2^ = 0.827. This was followed by AA (R^2^ = 0.354), SH (R^2^ = 0.247) and ST (R^2^ = 0.019). Data assessed using an adapted ANOVA model showed no statistically significant difference (p = 0.291) between the screws.

**Conclusions:**

The continuous development of HCSs has resulted in very comparable implants, and thus, at this time, other factors, such as surgeons’ experience, ease of handling and price, should be taken into consideration.

## Introduction

Scaphoid fractures are common and problematic in young active males[1]. The incidence of scaphoid fractures is second only to distal radius fractures among all wrist injuries[2,3]. Because conservative treatment requires a long period of rest, scaphoid fractures increase the use of worker’s compensation and may result in socioeconomic losses to families[4].

Fracture displacement is considered an absolute indication for fracture reduction and fixation. In particular, acute fractures of the proximal pol, fractures with preexisting cystic bone formation, transscaphoid perilunate dislocations, untreated fractures that are more than 4 weeks old and nonunion fractures require rigid stabilization. Internal fixation with a screw, via either the dorsal or the palmar approach, has become the gold standard[1,4–6]. Additionally, the expectations of young individuals to be able to quickly return to work are increasing the trend of fixing nondisplaced scaphoid fractures[1,7,8].

Over the last three decades, scaphoid fracture stabilization using a single screw has been developed. Although the first screw to use a pitch difference between the proximal and distal threads was biomechanically inferior to the conventional screw designs[9,10], the concept of scaphoid fracture treatment introduced by Herbert was a milestone in the reduction of scaphoid nonunions and long-term complications. The original headless compression screw (HCS) invented by Herbert and Fisher[11] was not cannulated, and it has provided an incentive in the last few years to develop multiple generations of screws with increased biomechanical advantages.

The mean time to union after internal screw fixation of acute, nondisplaced fractures of the scaphoid waist is approximately 6–8 weeks, which is consistently shorter than the time to union after nonsurgical treatment (12–15 weeks)[12–14]. The main problem with operative treatment is the higher complication rate compared to plaster treatment[15]. This difference can result from an improper technique of internal fixation, suboptimal screw placement or insufficient durability of stabilization if the fracture is not casted postoperatively. The operative technique can be improved with adequate training and surgeon experience, although it is unclear whether new implants offer more reliable fracture stabilization.

The purpose of this study was to compare the robustness of internal fixation with four leading HCSs of varying design under cyclic loading in a simulated scaphoid fracture. Each screw offers a different method of building compression. The primary outcome was the angulation allowed by each screw between the bone fragments after loading.

## Materials and Methods

We obtained 45 fresh-frozen scaphoids with written approval from the Ethics Committee of the Landesaerztekammer Rheinland-Pfalz, Mainz, Germany (Consent No. 837.088.07). Samples were procured from the donation center at The Institute of Anatomy of The Johannes Gutenberg University Mainz (JGU).[16] The bones were explanted from cadavers and frozen at -24°C until CT measurements, instrumentation and testing.

After performing a CT scan of all specimens (Siemens Somatom, Munich, Germany), bones that were previously broken, were malformed by arthritis or were too small to fix in the loading device were excluded, as a typical patient with a scaphoid fracture is unlikely to have these features. Forty bones were included in the study. Further, 3D objects were built to calculate the mean voxel value (Hounsfield units). The air entrapments within bones were excluded prior to the calculation, as they do not provide support for the screw fixation. The mean voxel value in Hounsfield units was noted in previous studies to correlate with bone mineral density units, and thus, these values were accepted as sufficient for the purpose of our study[17]. The mean age of the specimens was 77 years (range 61–97). Mean bone density was 470.6 HU (range 276.3–813.9, SD 21.9, CI 95% 44.4)

Bones of similar length were stratified into 10 groups of four bones each to minimize measurement error in the loading device. We compared differences in the bone density between the groups for AA, MH, ST and SH, but there was no statistical significance (p = 0.505). In every group of four bones, one screw was randomly assigned to each bone. (S1 and S2 Tables)

### Implants

**Fig 1:**
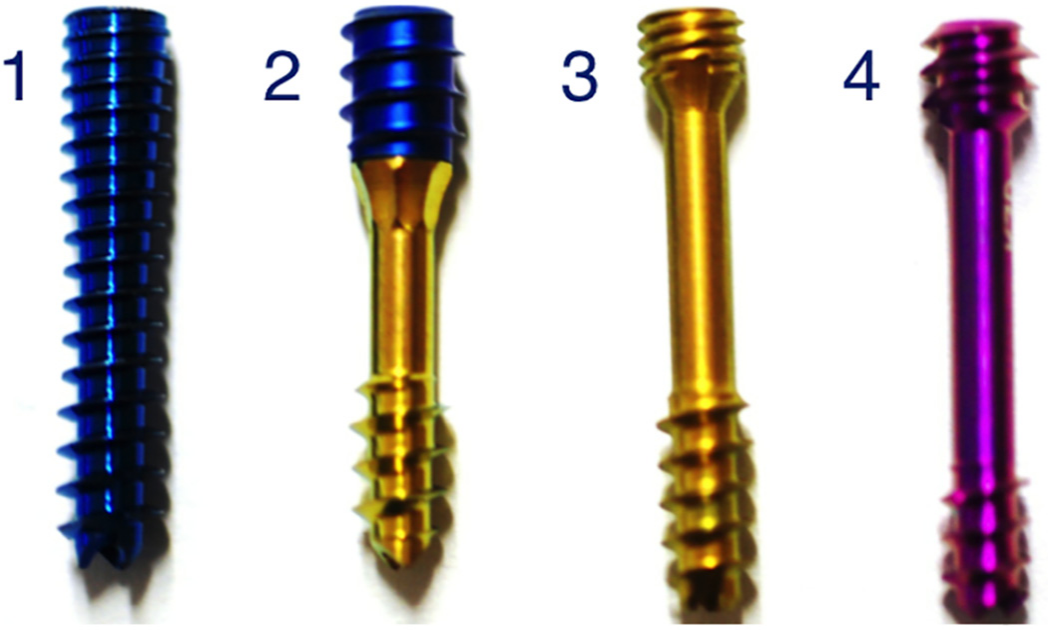
Competing screws. 1—Acumed Acutrak 2 Mini (AA), 2—Stryker TwinFix (ST), 3—Synthes 3.0 HCS with a long thread (SH), 4—KLS Martin HBS 2 Midi (MH).

We chose four implants that are currently on the market from leading manufacturers. Each screw has a different mechanism for building compression (Fig 1). Three screws were previously tested for loss of compression force over time in a different experiment[18]. The fourth screw is the updated version of the well-known Herbert screw.

1. Acumed Acutrak 2 Mini (AA; Acumed, Hillsboro, OR, USA) has a conical screw design and a continuous, variable thread. The wider thread pitch at the tip of the screw penetrates the bone faster than the finer trailing thread, which gradually compresses the two fragments as the screw is advanced. (S1 Text)
2. Stryker TwinFix (ST; Stryker, Kalamazoo, MI, USA) has proximal and distal screw threads that work independently to allow for in situ dynamic adjustable interfragmentary compression. Edges at the screw body enable reaming at the core hole for the trailing screw thread. This screw requires a special screwdriver with two concentric hexagonal heads and a clutch. (S2 Text)
3. Synthes 3.0 HCS (SH; Synthes, Solothurn, Switzerland) has an additional instrument, a compression sleeve, which brings the fractured fragments together and allows for accurate placement of the screw under the cartilage surface. The screw is first screwed into the cannulated compression sleeve with a trailing thread. It is then inserted into the bone using a firmly connected compression sleeve. As the compression sleeve is wider than the head of the screw, once the tip of the compression sleeve lies on the bone, the fracture gap is closed and compressed by further turning the screw with the sleeve. Finally, the screw is countersunk into the bone with an additional screwdriver while the compression sleeve is held stationary. The pitch of the trailing thread is doubled, with the same lead between the leading and trailing thread. Therefore, with one rotation of 360°, both the leading and trailing threads move the same distance. As a result, no extra compression is generated during countersinking of the screw into the bone. We used screws with a long leading thread. (S3 Text)
4. KLS Martin HBS 2 Midi (MH; KLS Martin; Tuttlingen, Germany) offers a design with differential proximal and distal pitch to induce interfragmentary compression according to the idea of Herbert[11]. It is self-drilling and self-cutting and also works in the reverse direction. The overall management of instruments was addressed during the development of the second generation. Technical specifications are outlined in Table 1. (S4 Text)

**Table 1:** Specifications of the tested screws. Ø–diameter.

	AA	ST	SH	MH
**Guide pin size**	Ø1.1 mm	Ø1.0 mm	Ø1.1 mm	Ø1.1 mm
**Insertion technique**	Self-tapping; optionally self-drilling	Self-tapping; leading and trailing head move independently	Self-tapping; optionally self-drilling; compression sleeve	Self-tapping; optionally self-drilling and compression sleeve
**Screwdriver**	Hex Driver	Double Hex with a clutch	T8 StarDrive	T8 StarDrive
**Screw diameter of the trailing/ leading thread [mm] and thread type**	Ø3.6/3.5; continuous, conical, variable thread pitch	Ø4.2/3.2; identical thread pitch and lead	Ø3.5/3.0; double trailing thread with the same lead as the leading thread	Ø3.9/3.0; thread increases from 1.0 to 1.25 mm

### Test set-up

**Fig 2:**
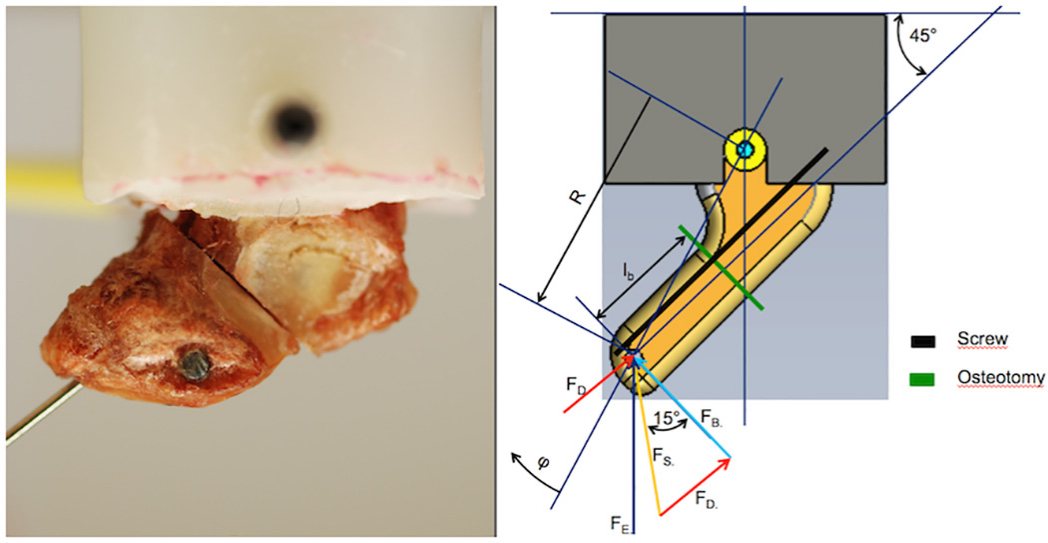
Specimen fixation and forces. Left side—scaphoid fixed in PMMA cement before the testing procedure. K-wire located in the screw to indicate the 45° angle of the screw to the ground plate. Gray points are the axes, which are parallel through the bone to allow for loading and measurements. Right side—force vectors and angles used in this study.

A new loading device was developed with the Physics Division of The RheinMain University of Applied Sciences, Wiesbaden, Germany. Each bone was first sawed at an angle perpendicular to the long axis in the waist, simulating the most common B2 fracture according to The Herbert Classification[11]. Afterwards, the bone fragments were fixed with the appropriate screw according to the manufacturer’s guidelines and with original instruments. Care was taken to introduce screws centrally with the use of a scaphoid jig, as described by Menapace et al[19], (S1 Fig). The self-drilling options available for AA, SH and MH were not used. In the next step, two parallel axes with 2-mm diameters were placed in the proximal and distal fragments to allow for loading and concurrent measurements. The prepared scaphoid was then fixed at a 45° angle to the ground plate of the loading device with PMMA cement at the proximal pole, as shown in Fig 2.

The loading force coming from a screw gear driven by a stepping motor worked indirectly through a free-floating plate with the vector F_S,_ which resulted in the decomposition of vectors into F_D_ and F_B._ R was the continuously measured distance between parallel axes. φ was the continuously measured angulation between the fragments during loading. l_b_ was the distance from the middle of the distal axis to the osteotomy.

**Fig 3:**
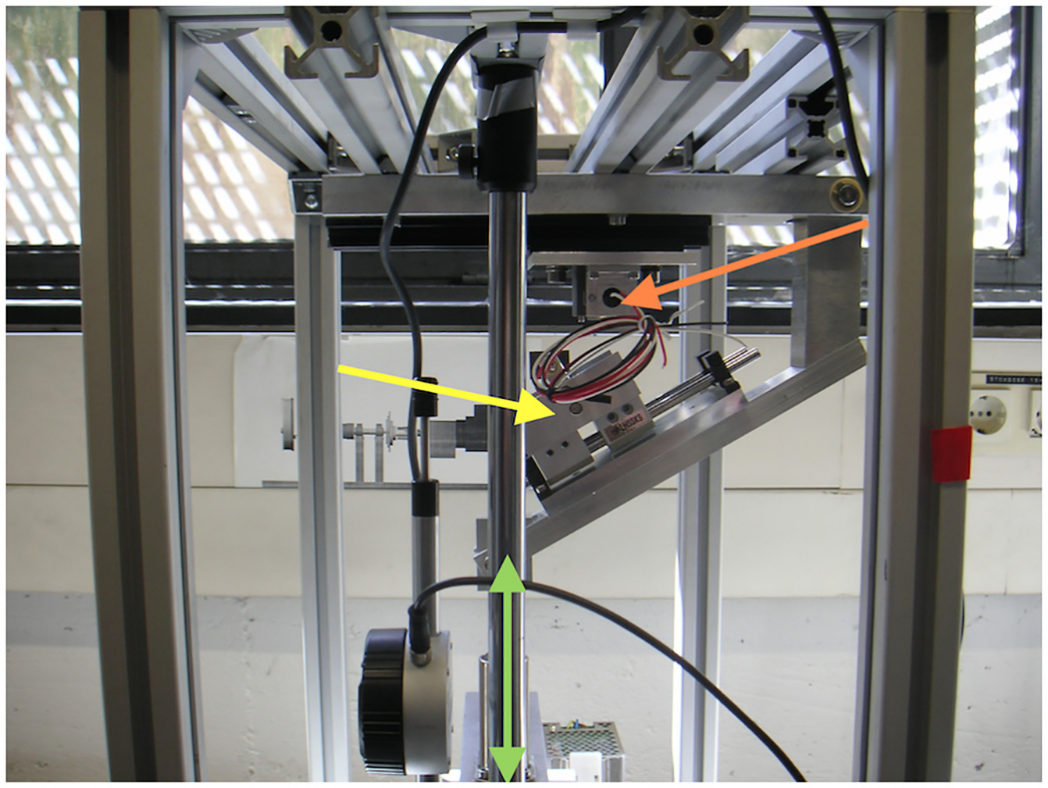
Loading device. Red arrow—position of the scaphoid during the testing procedure. Yellow arrow—free-floating loading plate. Green arrow—vector of a loading force from a motor.

The cement cube was then interposed into the loading machine as shown in Fig 3. The proximal end of the implant was not fixed in cement.

**Fig 4:**
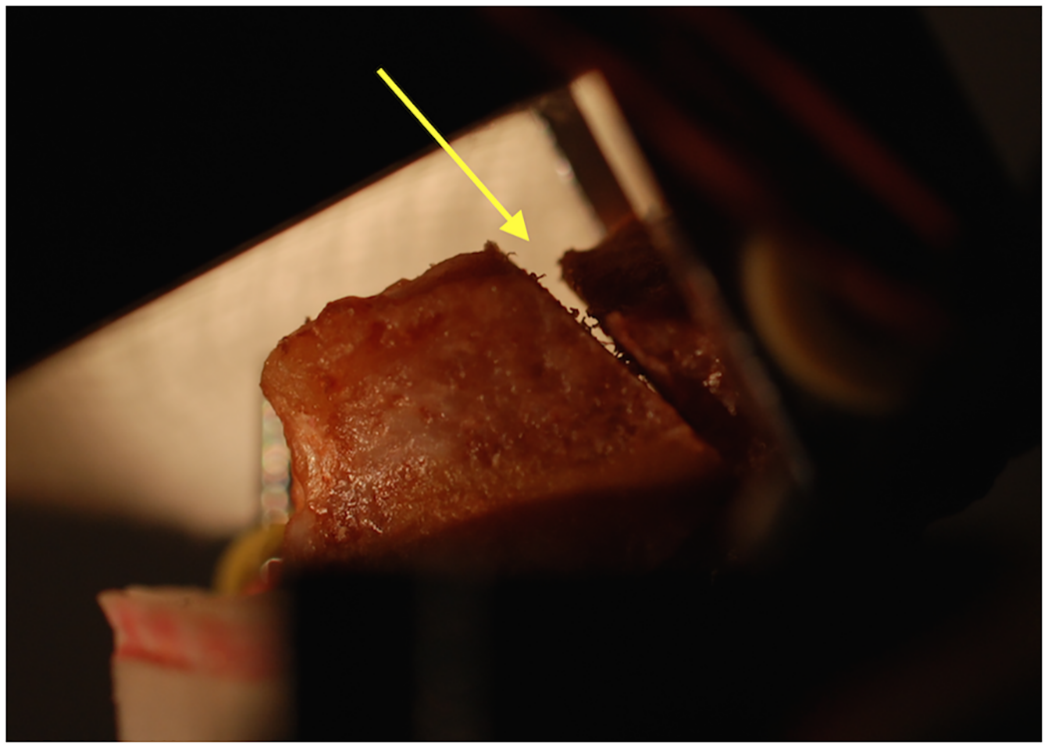
Scaphoid during the test procedure. Arrow shows an opening of the simulated fracture site under loading.

The actual loading of the scaphoid during daily motion remains unknown. In the pre-testing phase, we first checked the elasticity of the screws in sawbone models. In the next step, bone specimens with different loads were tested to identify the appropriate loading force at which no failure would occur before 2000 cycles and at which the results would be recognizable (Fig 4).

The appropriate load (62.5–90 N) was computed for different bone lengths to assure the same bending moment of 800 Nmm for each specimen. Bones were then loaded over 2000 cycles. ´LabVIEW Professional 2011´ (National Instruments, Austin, TX, USA) was used to control the device and collect the data.

### Statistical methods

All statistical analyses presented in this report were produced using IBM SPSS Statistics version 22 (IBM, Armonk, NY). We performed a univariate analysis of variance (ANOVA). The effects of each screw on the φ were adjusted to the bone density of the specimens. P values <0.05 were considered statistically significant. Linear regression lines were drawn to analyze the individual properties of each screw.

## Results

Thirty-six bones underwent the complete procedure (10 with AA, 9 with ST, 8 with MH and 9 with SH). In one specimen, the head of ST lost grip with the screwdriver during the screw insertion due to a quick wearing of the screw head, and further closing of a fracture gap was not possible. This specimen had high bone density. Additional, there was one case of breakage of the blue ring holding a trailing thread in ST, but it did not significantly influence the residual stability of fixation. In the remaining three cases, there were problems building up compression in the osteoporotic bone specimens.

**Fig 5:**
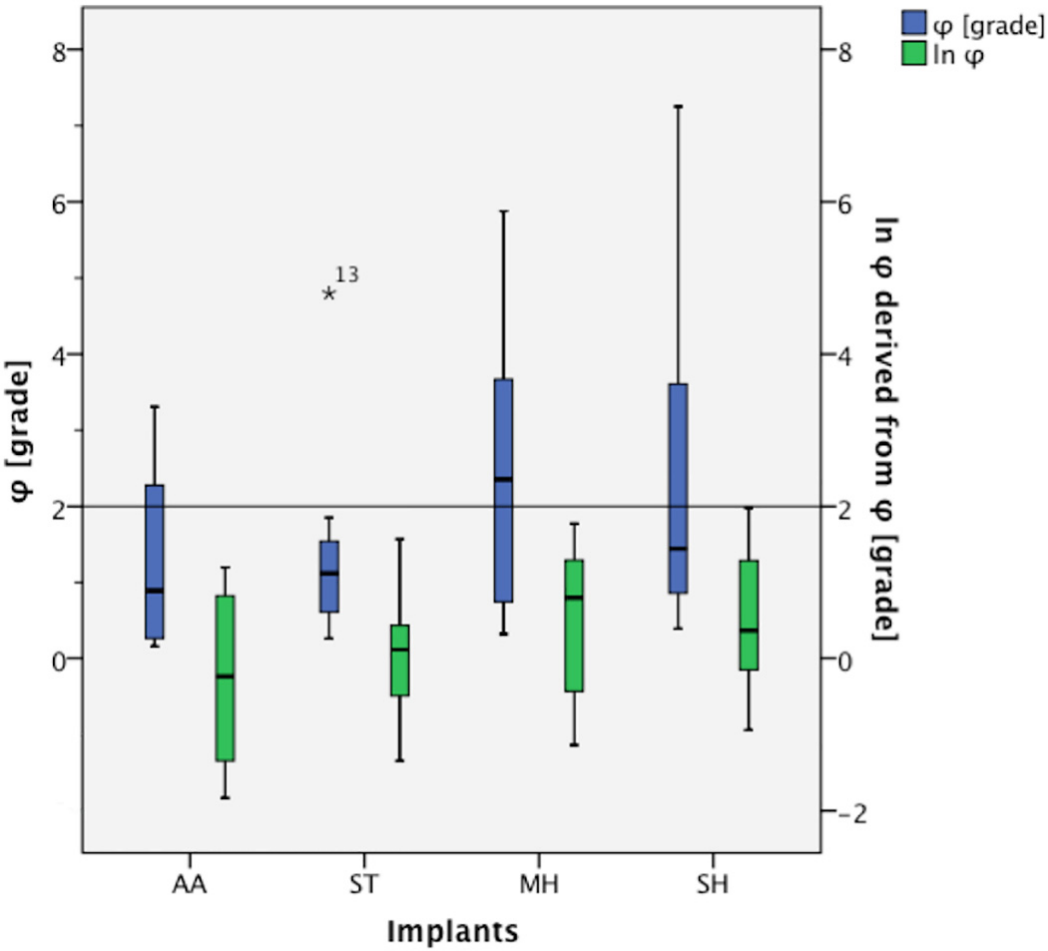
Distribution of φ [grade] and ln φ.

The values of φ [grade] appeared skewed, so to adjust the statistical model, we performed a logarithmical conversion of the angle difference (Table 2 and Fig 5). Further data were assessed using an adapted ANOVA model with R^2^ = 0.327. After adjustment for bone density, no statistically significant difference (p = 0.291) between the screws could be shown. The factor that significantly influenced stability of the fixation was bone density (p = 0.003). In this case, further between-subject comparisons were redundant.

**Table 2:** Descriptive statistics of φ [grade] and ln φ.

**Implants**		**N**	**Minimum**	**Maximum**	**Mean**	**Median**	**SD**	**Skewness**
**AA**	φ [grade]	10	.16	3.31	1.27	**0.89**	1.13	.63
	ln φ	10	1.83	1.20	-.25	-.24	1.22	-.84
**ST**	φ [grade]	9	.26	4.80	1.41	**1.12**	1.39	2.14
	ln φ	9	-1.35	1.57	-.03	.11	.93	.02
**MH**	φ [grade]	8	.32	5.88	2.47	**2.36**	1.96	.57
	ln φ	8	-1.14	1.77	.49	.80	1.09	-.60
**SH**	φ [grade]	9	.39	7.25	2.41	1.44	2.27	1.36
	ln φ	9	-.94	1.98	.47	0.36	.98	.15

**Fig 6:**
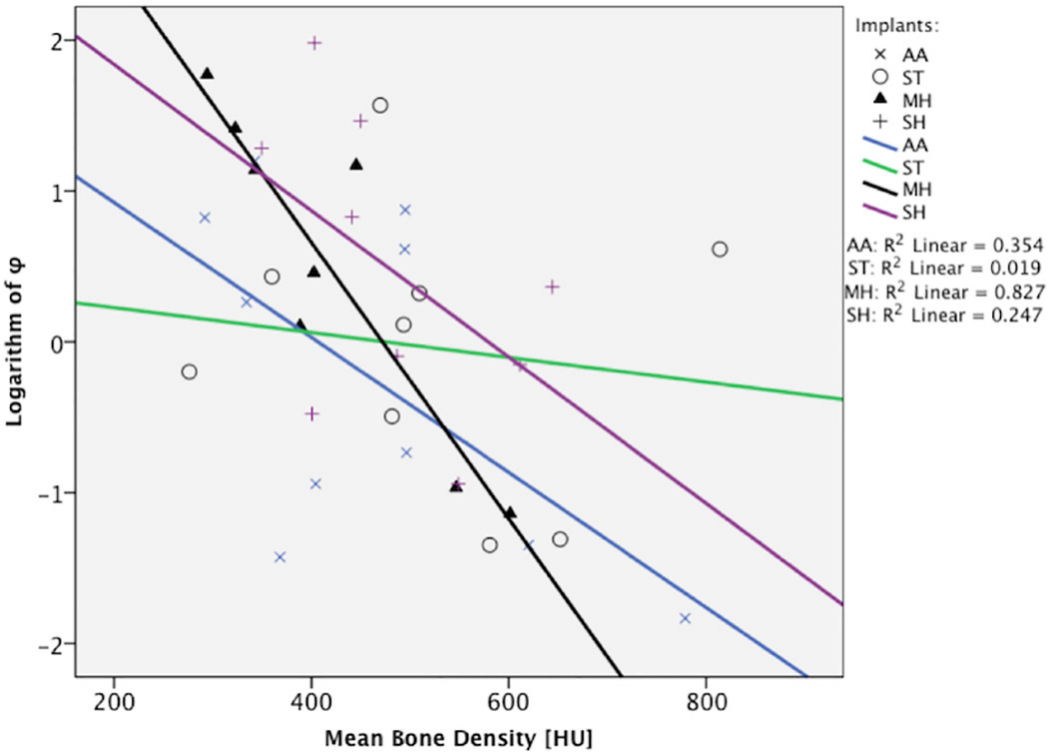
Linear regression analysis of the tested screws.

MH showed a coefficient of determination of R^2^ = 0.827, followed by AM (R^2^ = 0.354), SH (R^2^ = 0.247) and ST (R^2^ = 0.019). The results of linear regression are shown in Fig 6.

## Discussion

The purpose of this study was to compare four different designs of leading HCSs in a simulated scaphoid fracture. The primary outcome was the degree of dislocation between bone fragments under cyclical loading.

No statistically significant difference between the screw designs could be shown. The median angle difference φ, as an indicator of loosening of the internal fixation, was lowest in AA (0.89°) and ST (1.12°), followed by SH (1.44°) and MH (2.36°). ST offered similar bone purchase for bones with low or high bone density. However, according to our findings, MH showed the most predictable results of dislocation of the fracture fragments expressed by the highest coefficient of determination (R^2^ = 0.827). The results from the other screws showed a wider spread, and thus further conclusions are less reliable.

MH and AA allowed low angulation in bones with higher density, which is common in patients with acute scaphoid fracture (Fig 6). Considering that a typical patient with a scaphoid fracture is young and male, we expect good bone quality and good results with these screws. The results of SH were similar to those of other screws, although it needs to be inserted carefully to avoid reduction of the compression force[18,20].

We were unable to measure personal preference of handling among screw types. Screwdrivers with heads that are large enough to transmit appropriate torque and that have a large, soft rubber grip allow high precision of insertion of AA. In contrast, ST requires fine mechanics of a rotating thread and a double hex screwdriver, which can cause problems during insertion, as mentioned in the Results. Advantages of MH include additional features such as the optional compression sleeve or different lengths of the leading thread.

The micro-motion at the fracture site and the resulting shearing forces lead to fibrous tissue formation and failure of bony healing[21]. The scaphoid, which links the distal carpal row through ligaments coming from the trapezium, trapezoid, and capitate to the proximal row by the scapholunate ligament, needs to transfer complex forces that can be divided into bending and shearing[22]. As a result, a successful fixation device needs to offer high stability to resist these complex forces. As 75% of the scaphoid is covered with a joint surface[23], the optimal fixation device should be able to withstand loading during daily activities to allow direct bone healing. Otherwise, early wrist arthritis due to callus formation may occur.

Compression offered by older HCSs has been thoroughly tested[10,24–26]. To compare new screws with a conventional head screw, our group has already tested AA, ST and SH against a 2.0-mm cortical screw for changes in compression with time after screw insertion[18]. In this study, ST offered the highest amount of compressive force over 12 hours, followed by AA and SH. All HCSs were significantly better than the reference screw.

Optimal screw placement in the scaphoid according to biomechanical and computer-aided studies was also extensively examined[19,27–30]. Next, we analyzed the literature for studies examining stability of fracture stabilization of cadaver scaphoid fractures under cyclical loading. There is very little in the literature regarding testing cadaver scaphoid bone fixation, likely due to the technically demanding test procedure. Toby et al. studied biomechanical properties of the original Herbert screw against either an AO 3.5 mm cannulated screw, a Herbert-Whipple screw, an Acutrak cannulated screw or a Universal Compression screw[31]. His group compared matched pairs of formalin-fixed scaphoids with a simulated scaphoid fracture under cyclical bending. They proved that different screws varied significantly in their ability to resist cyclical bending loads. The AO screw and Herbert-Whipple screws showed superior resistance against cyclical bending compared to the Herbert screw. The Acutrak screw was significantly better than the Herbert screw only with regard to the number of cycles required to produce 1.0 and 1.5 mm of displacement. However, fresh bones, which were used in our study, were proven by Burkhart et al. to better represent in vivo conditions than formalin-fixed bones[32]. Currently, similar studies on the new generation of screws are lacking.

Our experiment has weaknesses. There was large variation in the density of the old-age cadaver bone specimens during in vitro testing. As in many other studies, biomechanical testing shows differences between implants in an artificial situation and under artificial loading. The axes of movement in the wrist were examined and were shown to be very complex[33]. The complexity of movement of the scaphoid in a wrist could not be simulated in vitro in a cost-effective manner due to its small size, difficulties in fixation, unreliable loading and measurement of results. Similar problems occur in a whole-wrist model and during testing of an isolated bone. As nonunions tend to collapse into a flexed position[34] with wearing of the volar cortex, an uniaxial loading simulating flexion was chosen. Screws used for internal fixation should be able to prevent this effect. Isolating the scaphoid allows fine and more direct measurements of displacement and loss of fixation by the screws than in the whole-wrist model. Due to its complex geometry and non-homogeneous nature, actual scaphoid rather than sawbone models were found to be advantageous.

Another limitation appeared after study completion. The osteotomy we performed was made perpendicular to the long axis of the scaphoid, as we wanted to represent the most common type of waist fracture. In a recent paper, based on 3D CT assessment, Luria et al. refuted this assumption and found that most waist fractures have a volar distal to dorsal proximal (horizontal oblique) inclination[35]. This fact may increase instability of the internal fixation with a screw placed in the long axis of the bone that is not perpendicular to the fracture line.

After the primary statistical analysis, two cases were found to be outliers. The rotating head of one ST screw was found to have broken free, although the screw provided high stability. In one SH, the interfragmentary compression could not be built due to a technical error during implantation. In this case, the SH was introduced too deep after leaving the compression sleeve. This issue is a known problem of the insertion technique that can cause almost complete lack of compression[18,20]. Despite these concerns, the screws were retained in the analysis due to the ‘intention-to-treat’ design, which has implications for daily use. Both of these problems may occur in the operating theater, and surgeons need to be prepared for them when using these implants.

The question of whether different designs of modern HCSs have benefits for patients remains open. We seek better implants due to the increasing pressure from patients for greater treatment efficiency, quicker healing time and earlier return to work. However, an often unstated problem is that orthopedic surgeons often look for state-of-the-art devices solely to offer a novel device to fulfill patients’ desire for the latest trends in treatment. Based on our results, we advise surgeons to concentrate on problems such as appropriate surgical technique, screw length, placement and correct soft tissue management. These factors reduce the risk of poor outcomes after scaphoid fracture more than the choice of a novel implant.

### Conclusions

The continuous development of HCSs has resulted in comparable implants. According to our results, MH and AA offered slightly more stability in bones with higher density. The ST design offered consistent biomechanical results but may result in a malfunctioning implant, and the data we obtained are not reliable. SH is a cheaper alternative that is not statistically inferior to other screws. Finally, the choice of an implant should be made according to the surgeons’ experience with the device, its ease of use and the local price of the screw.

## Supporting Information

S1 Fig Scaphoid jig. (JPG)

S1 Table Data table. (SAV)

S2 Table Randomization table. (XLSX)

S1 Text Technique guide for AA. (PDF)

S2 Text Technique guide for ST. (PDF)

S3 Text Technique guide for SH. (PDF)

S4 Text Technique guide for MH. (PDF)

S5 Text AJE Certificate of English proof-read. (PDF)
