# Supplementary figures and images for: Impact of Different Screw Designs on Durability of Fracture Fixation: In Vitro Study with Cyclic Loading of Scaphoid Bones
Source: PLoS One. 2016 Jan 7;11(1):e0145949. doi: 10.1371/journal.pone.0145949 (PMC4704798; doi:10.1371/journal.pone.0145949)

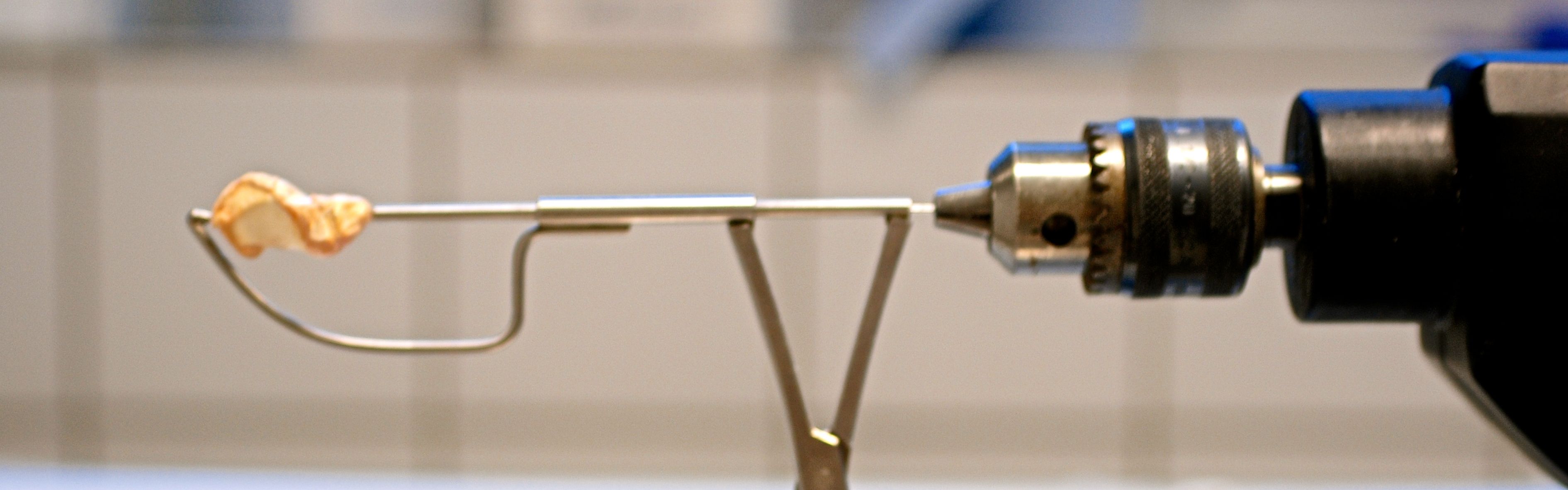

Supplement: S1 Fig — (JPG) [file pone.0145949.s001.jpg]
