# Supplementary material for: Impact of Different Screw Designs on Durability of Fracture Fixation: In Vitro Study with Cyclic Loading of Scaphoid Bones
Source: PLoS One. 2016 Jan 7;11(1):e0145949. doi: 10.1371/journal.pone.0145949 (PMC4704798; doi:10.1371/journal.pone.0145949)
Supplement: S1 Text — (PDF) [file pone.0145949.s004.pdf]

ACUMED®

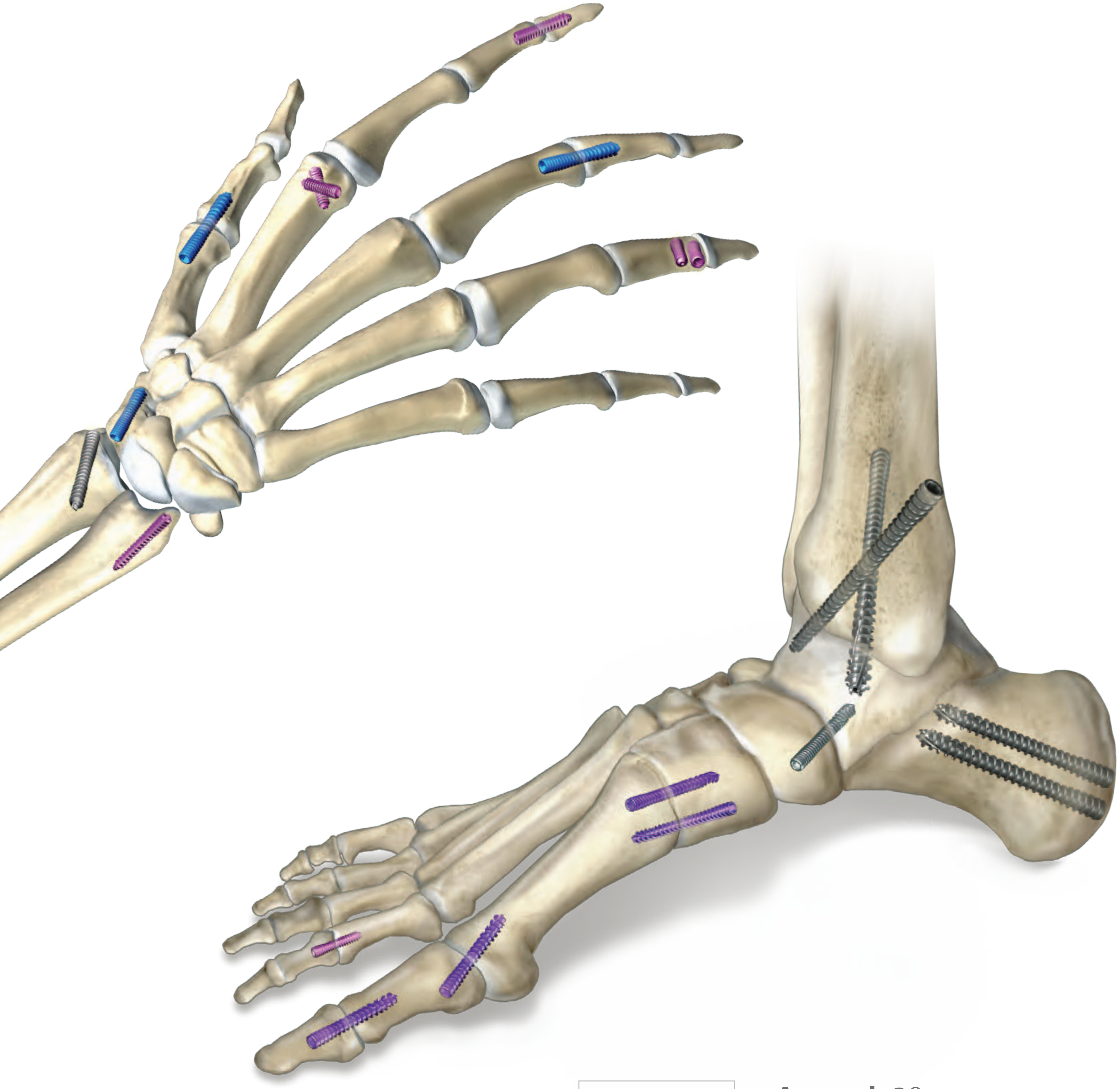

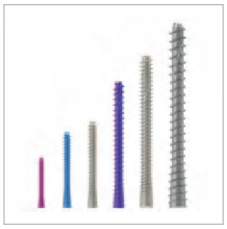

# Acutrak 2® Headless Compression Screw System

Surgical Technique Guide

# Acutrak 2® Headless Compression Screw

*Acumed® is a global leader of innovative orthopaedic and medical solutions.*

*We are dedicated to developing products, service methods and approaches that improve patient care.*

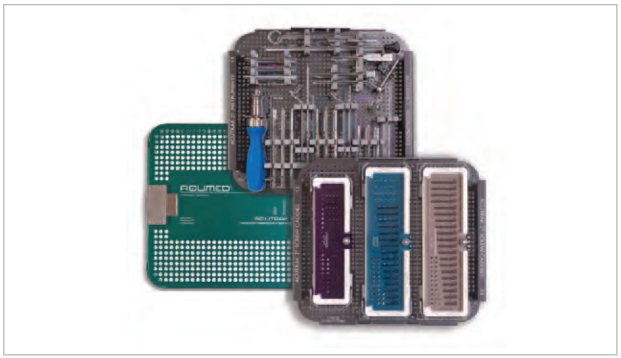


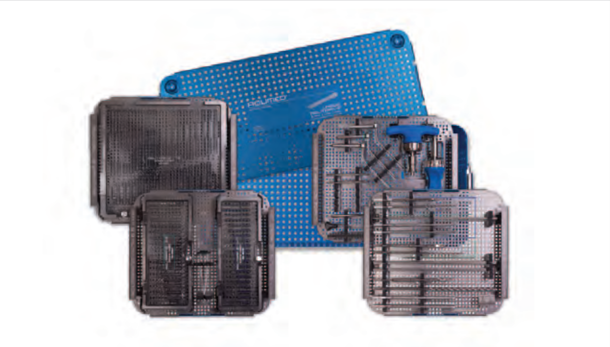

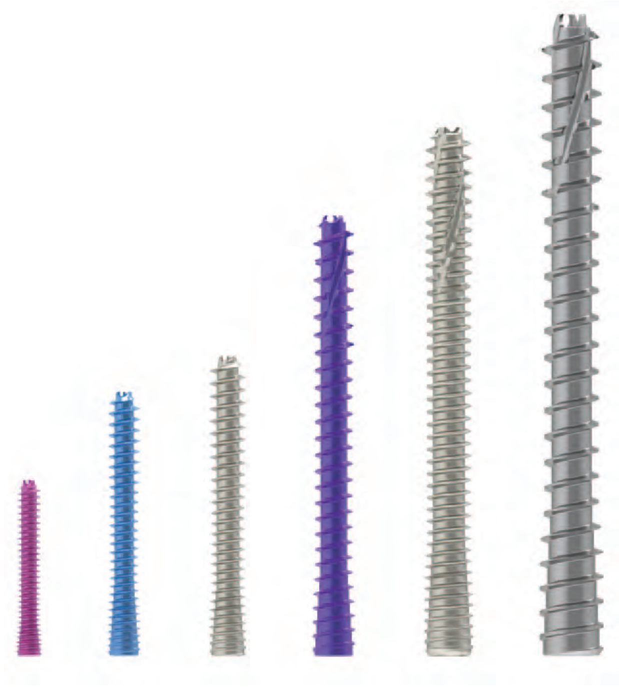

Since its introduction in 1994, the Acutrak® Headless Compression Screw Technology has revolutionized the way surgeons treat fractures, fusions, and osteotomies. The Acutrak 2® represents the latest innovation in fully threaded headless fixation. Long term surgeon feedback has helped develop a high performance implant with intuitive instrumentation designed to simplify the surgical technique.

The Acutrak 2® family is comprised of 63 unique screw size options to fit a wide variety of applications throughout the body, from 2 mm X 8 mm up to 7.5 mm X 120 mm.

## Contents


Acutrak 2® Features 3

Indications for the Hand and Wrist 4

Indications for the Foot and Ankle 4

Acutrak 2® Quick Reference Chart 5

Surgical Techniques

- Volar Scaphoid Technique 6
- Dorsal Scaphoid Technique 8
- Jones Fracture Technique 10
- Calcaneal Osteotomy 12

Non-Sterile Screw Tray Overview 15

Ordering Information 16

**Headless -** Allows the titanium screws to be implanted in and around articular regions with minimal risk of impingement or soft tissue irritation.

**Variable Thread Pitch -** The wider thread pitch at the tip of the screw penetrates the bone faster than the finer trailing threads, compressing the two fragments gradually as the screw is advanced.

**Self-Cutting -** The cutting flutes on the distal tip of the screw allow the Acutrak 2® to be inserted with an advanced, straightforward surgical technique.

**Cannulated -** Facilitates accurate percutaneous insertion with minimal soft tissue dissection.

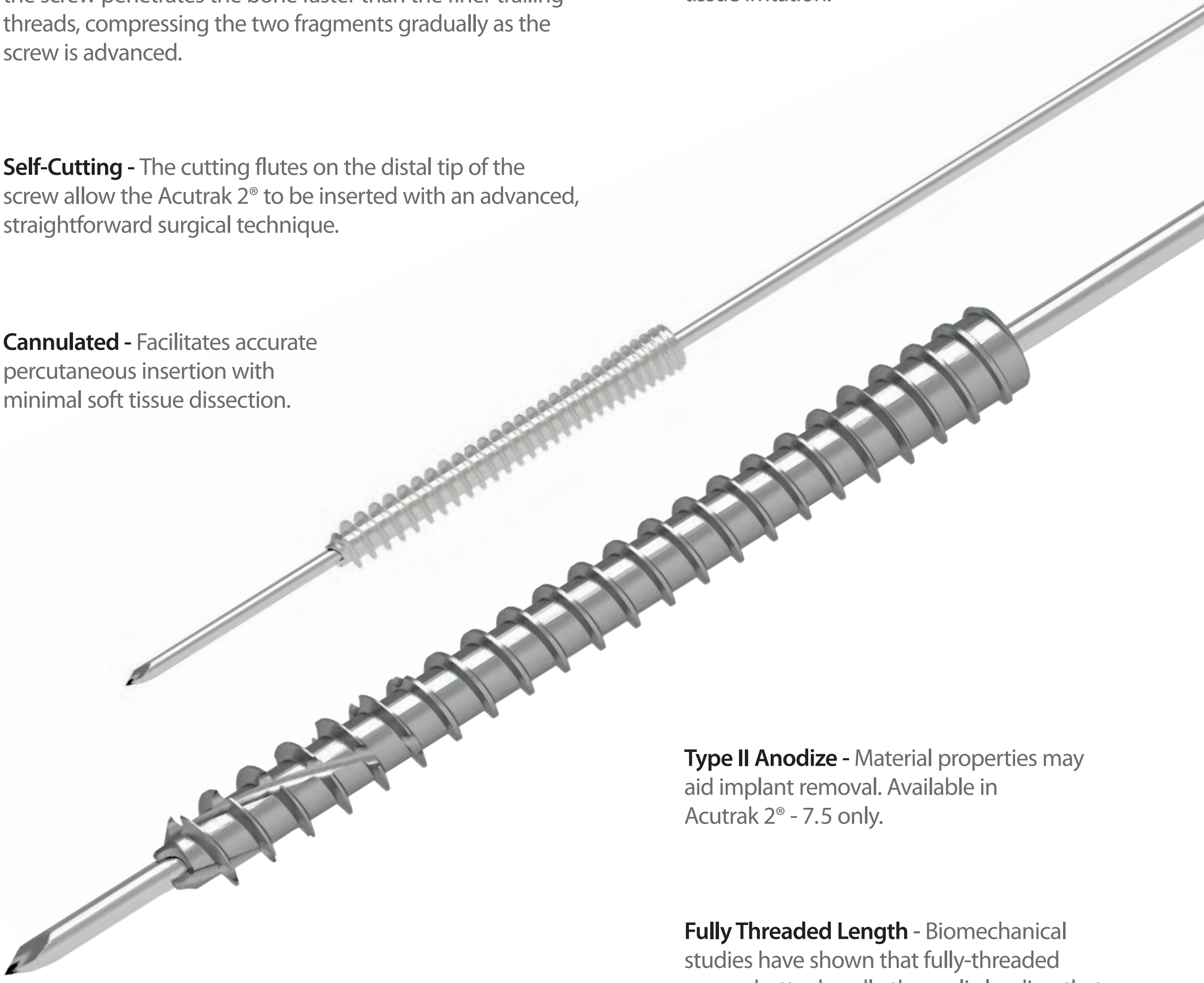

**Type II Anodize -** Material properties may aid implant removal. Available in Acutrak 2® - 7.5 only.

**Fully Threaded Length -** Biomechanical studies have shown that fully-threaded screws better handle the cyclic loading that may occur during healing. In addition, this feature allows a fracture or osteotomy site to lie almost anywhere along the length of the screw[1].

**Helical Relief Flutes -** Helical relief flutes on the distal portion of the screw assist in bone removal to ease screw insertion. Available in Acutrak 2® - 4.7, 5.5 and 7.5 only.

1. Wheeler, Donna. Biomechanical Assesment of Compresssion Screws. 350. Clinical Orthopedics and Related Research, 1998. 237-245.

# Acutrak 2® Indications for the Hand and Wrist

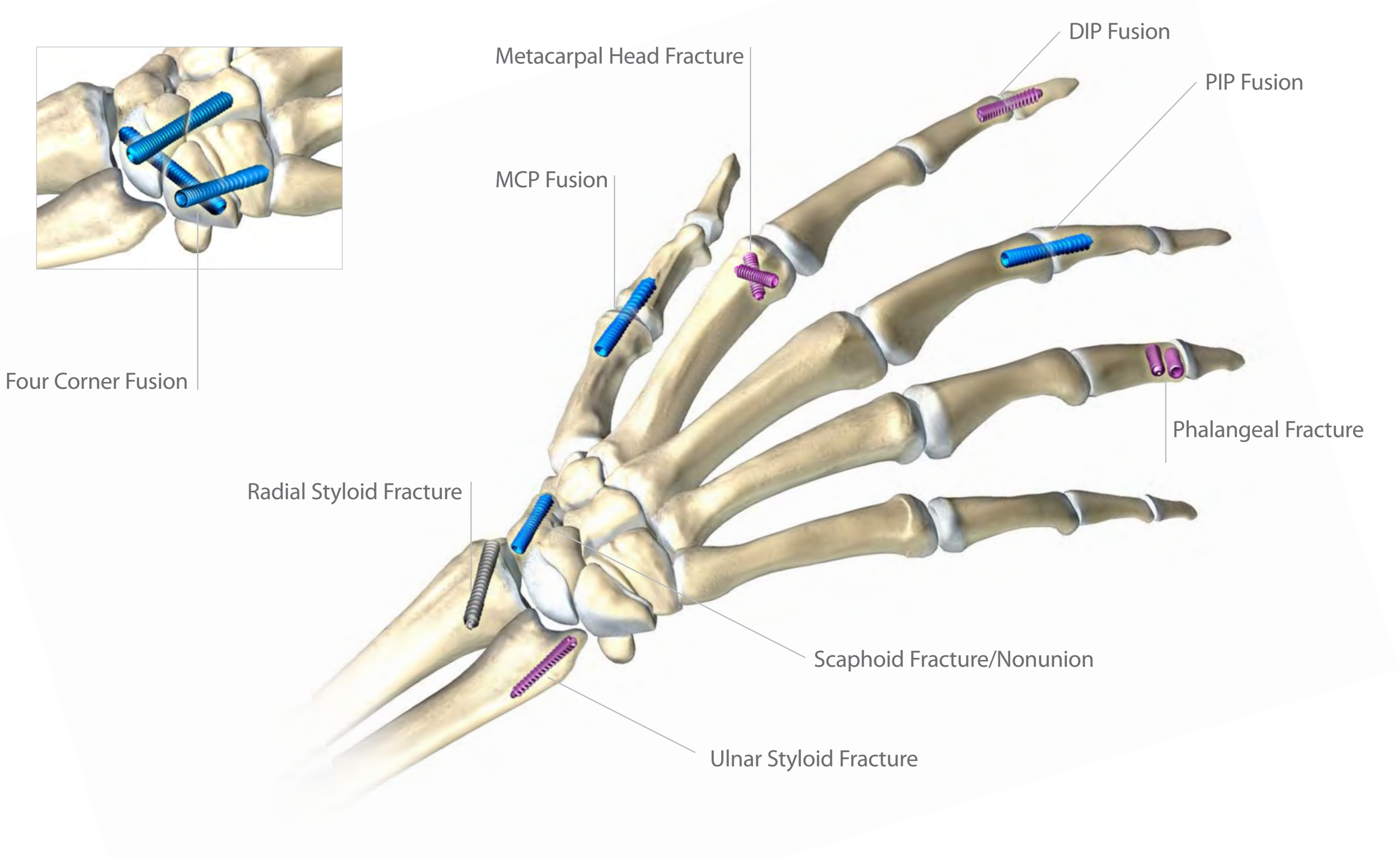


# Acutrak 2® Indications for the Foot and Ankle

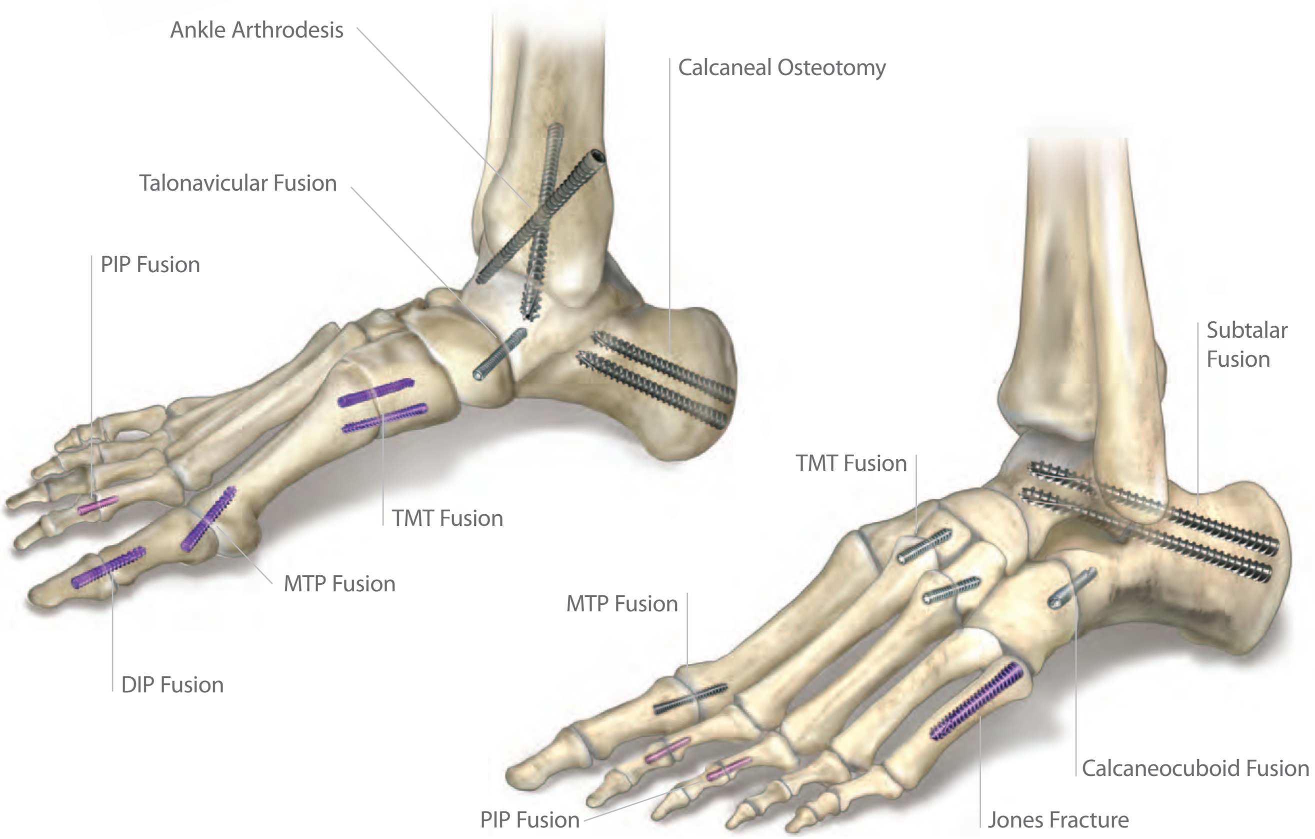

# Acutrak 2® Quick Reference Chart

| | Diameter | Length | Properties |
|---|---|---|---|
| **Micro** 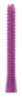 | Tip: 2.5 mm<br>Tail: 2.8 mm | 8 mm, 9 mm,<br>10 mm, 11 mm,<br>12 mm, 13 mm,<br>14 mm, 16 mm,<br>18 mm, 20 mm | • Use in lieu of a 2.0 - 2.4 mm Headed Screw<br>• 1.5 mm Hex Driver<br>• .035” (.88 mm) Guide Wire |
| **Mini** 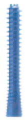 | Tip: 3.5 mm<br>Tail: 3.6 mm | 16 mm, 18 mm,<br>20 mm, 24 mm,<br>26 mm, 28 mm,<br>30 mm | • Use in lieu of a 3.5 - 4.0 mm Headed Screw<br>• 2.0 mm Hex Driver<br>• .045” (1.1 mm) Guide Wire |
| **Standard** 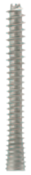 | Tip: 4.0 mm<br>Tail: 4.1 mm | 16 mm, 18 mm,<br>20 mm, 22 mm,<br>24 mm, 26 mm,<br>28 mm, 30 mm,<br>32 mm, 34 mm | • Use in lieu of a 3.5 - 4.0 mm Headed Screw<br>• 2.5 mm Hex Driver<br>• .054” (1.4 mm) Guide Wire |
| **4.7** 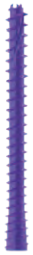 | Tip: 4.5 mm<br>Tail: 4.7 mm | 20 mm, 22 mm,<br>24 mm, 26 mm,<br>28 mm, 30 mm,<br>35 mm, 40 mm,<br>45 mm, 50 mm | • Use in lieu of a 4.5 - 6.5 mm Headed Screw<br>• 3.0 mm Hex Driver<br>• .062” [1.6 mm] Guide Wire |
| **5.5** 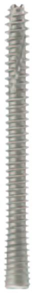 | Tip: 5.2 mm<br>Tail: 5.5 mm | 25 mm, 30 mm,<br>35 mm, 40 mm,<br>45 mm, 50 mm,<br>55 mm, 60 mm | • Use in lieu of a 4.5 - 6.5 mm Headed Screw<br>• 3.0 mm Hex Driver<br>• .062” [1.6 mm] Guide Wire |
| **7.5** 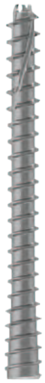 | Tip: 7.0 mm<br>Tail: 7.5 mm | 40 mm, 45 mm,<br>50 mm, 55 mm,<br>60 mm, 65 mm,<br>70 mm, 75 mm,<br>80 mm, 85 mm,<br>90 mm, 95 mm,<br>100 mm, 105 mm,<br>110 mm, 115 mm,<br>120 mm | • Use in lieu of a 6.0 - 7.5 mm Headed Screw<br>• 4.0 mm Hex Driver<br>• .094” [2.4 mm] Guide Wire |

# Volar Scaphoid Technique: Acutrak 2® - Micro, Mini, and Standard

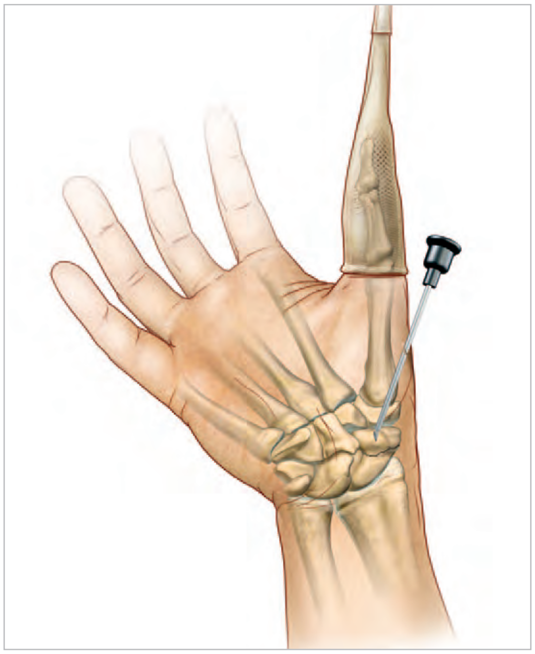

**1** The procedure can be carried out using the volar traction approach or using a conventional volar type approach with the arm supine on a hand table. The volar traction approach facilitates reduction of a displaced fracture and permits arthroscopy to ensure accuracy of the reduction. Fluoroscopy is used throughout.

The entry point is then located using a 12 or 14 gauge IV needle introduced on the antero-radial aspect of the wrist just radial to and distal to the scaphoid tuberosity. This serves as a trochar for the guide wire and is a directional aid to establish a central path along the scaphoid. The needle is then insinuated into the scapho-trapezial joint, tilted into a more vertical position and the position is checked on the under image intensifier. By gently levering on the trapezium this maneuver brings the distal pole of the scaphoid more radial and thus ultimately facilitates screw insertion. The entry point should be approximately 1/3 the way across the scaphoid from the tuberosity in the A/P plane and central in the lateral plane.

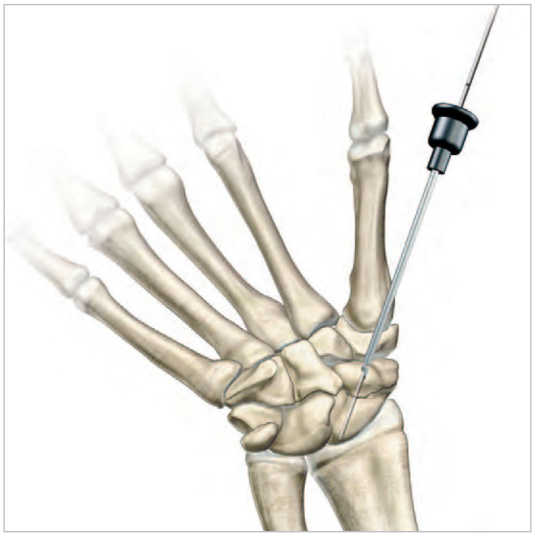

**2** Pass the guide wire through the needle and drill it across the fracture, continually checking the direction on the image intensifier and correcting as necessary, aiming for the radial aspect of the proximal pole. It is extremely important not to bend the guide wire and any adjustments in direction should be made using the needle as a guide rather than attempting to alter the line of the guide wire alone.

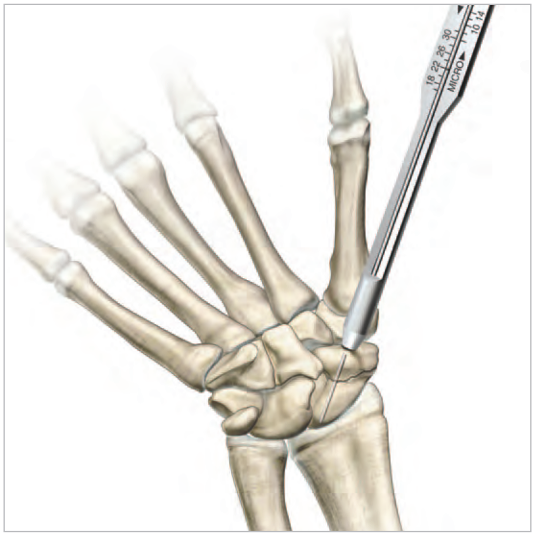


**3** Advance the guide wire to stop just short of the articular surface and the wire should not breach it at this stage. The position, alignment and length are checked once more. Make a simple stab incision at the entry point of the wire, and deepen this down to the distal pole of the scaphoid using a small hemostat and blunt dissection. This is a relatively safe zone with minimal risk to the adjacent neuro-vascular structures.

Determine the length of the screw either with the appropriate depth gauge or by advancing a second guide wire of the same length up the distal cortex of the scaphoid and subtracting the difference between the two. When using the volar approach, the correct screw size is 2-4 mm shorter than the measured length so as to ensure that the proximal tip of the screw is fully buried below the cartilage and the cortical surface.

**4** Advance the guide wire through the proximal pole of the scaphoid so as to exit on the dorsal aspect of the wrist. This is a precautionary measure to minimize the risk of inadvertent withdrawal of the wire during the reaming process and screw insertion and to facilitate removal of the proximal portion if the wire were to break. A second de-rotation wire can then be inserted in those cases where it is felt that there is a possibility of rotational instability of the fracture.

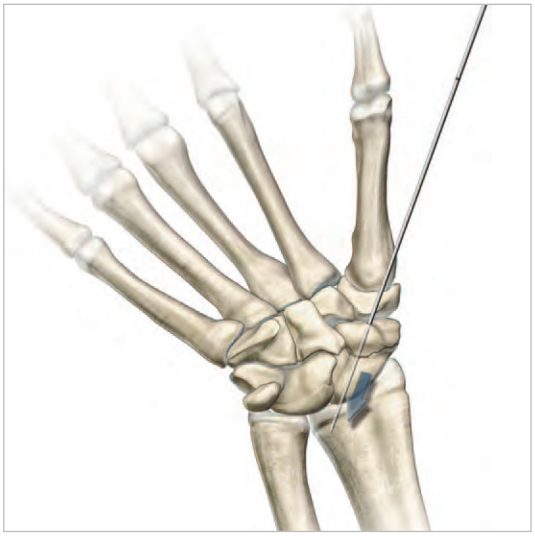

**5** Remove the 12 gauge needle and pass the cannulated profile drill over the wire using either a power drill or hand reamer stopping 1-2 mm short of the articular surface. The long drill is recommended to mitigate the effects of varying bone density and distraction upon screw insertion.

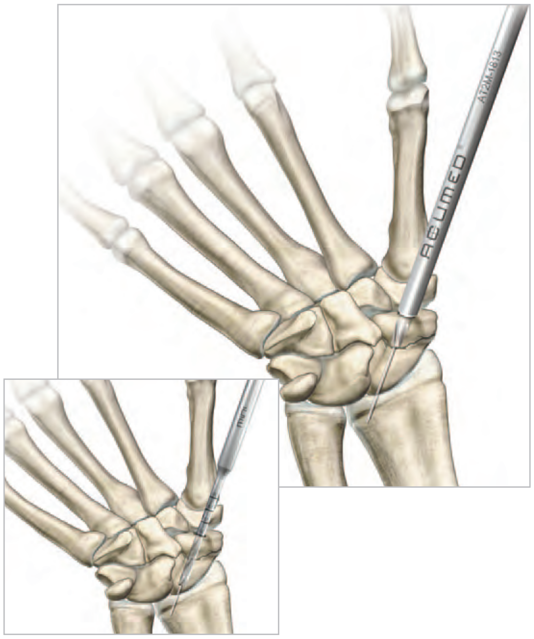


**6** The self-tapping screw is then advanced over the guide wire and the wire removed. Compression can then be confirmed radiographically on the image intensifier.

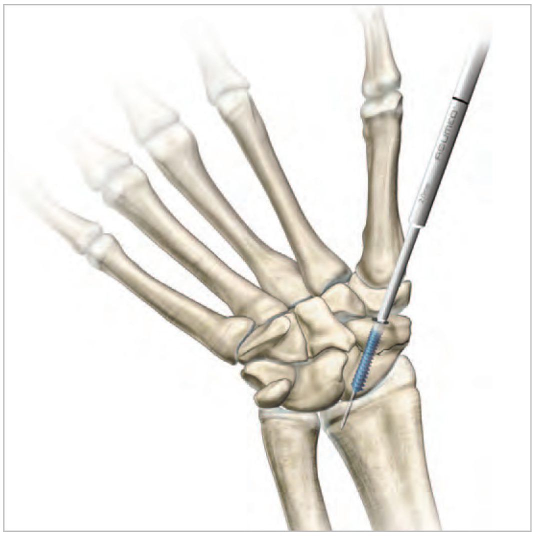

# Dorsal Scaphoid Technique: Acutrak 2® - Micro, Mini, and Standard

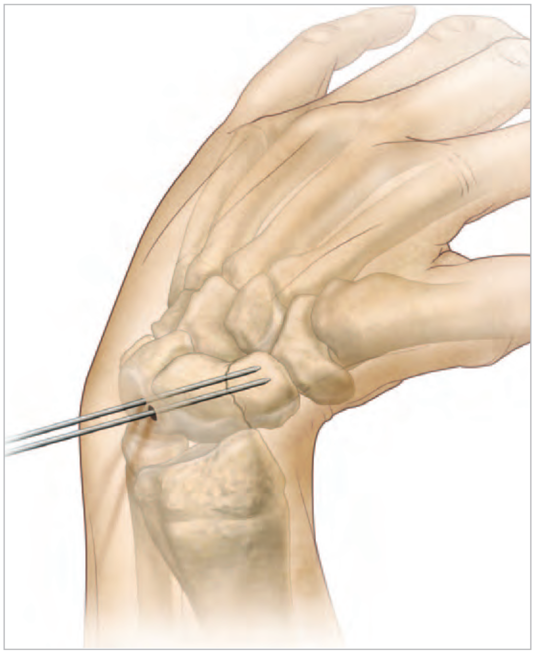

**1** The entry point in the proximal pole is at the tip of the scaphoid immediately adjacent to the sacpho-lunate ligament. This can be located either using an arthroscopy or mini open dorsal approach between the 3rd and 4th extensor compartments. Whichever approach is employed, it is essential to ensure that the guide wire does not transfix an extensor tendon.

Having established the entry point, introduce the appropriate guide wire aiming for the base of the thumb and check the position on the fluoroscope. Aim to place the leading edge of the guide wire in the subchondral surface of the distal pole of the scaphoid. Confirm the wire placement and depth under imaging.

**Optional:** A 14 gauge IV cannula is a useful aid in determining the entry point and acts as both a guide and soft tissue protector.

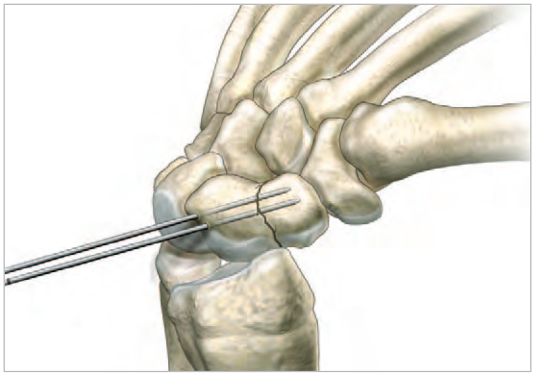

**2** If the fracture is unstable it may be helpful to place a second parallel guide wire using the parallel wire guides which are available for all three Acutrak 2® screw families.

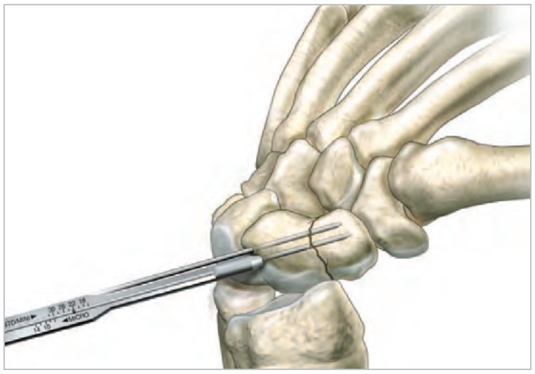

**3** Measure guide wire length using either the percutaneous screw sizer, or by placing a second wire at the entry point and subtracting the difference. The screw sizer cannot be used with the arthroscopic technique due to the limited access. 4 mm should be subtracted from the measured length to ensure that both ends of the screw are buried within the bone.

**4** Advance the guide wire through the far cortex so that it lies in the subcutaneous tissues. This minimizes the risk of accidental withdrawal of the guide wire while drilling and facilitates wire removal if it should break.

**Tip:** For most adult males the screw should not be longer than 26 mm, and in females 22 mm.

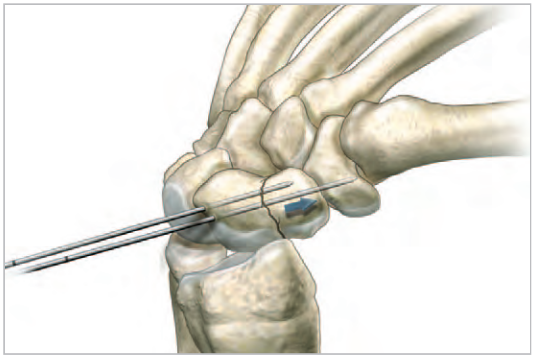

**5** Open the near cortex with the appropriate profile drill.

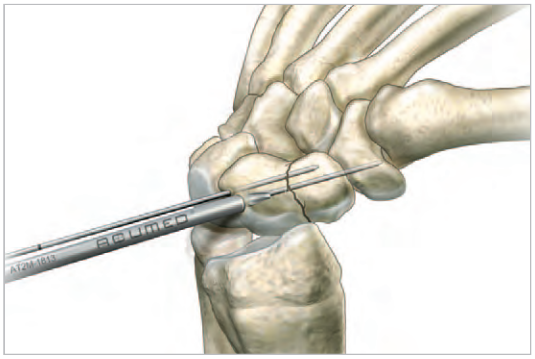

**6** Next, drill into the far fragment with the long drill. To be effective the drill only has to advance 4-5 mm past the fracture site.

**Tip:** The long drill is recommended to mitigate the effects of varying bone density and distraction upon screw insertion.

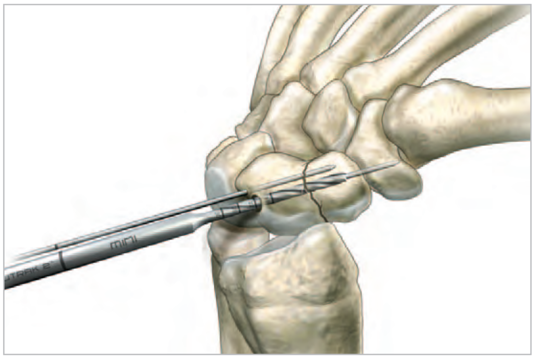

**7** Insert the correctly sized screw with the appropriate hex driver. If resistance is met upon insertion or if distraction occurs, stop, remove the screw, redrill with the long drill and re-insert the screw. Confirm placement and length of the screw on imaging, ensuring that both leading and trailing edges of the screw are beneath the articular surfaces. Finally remove the guide wires.

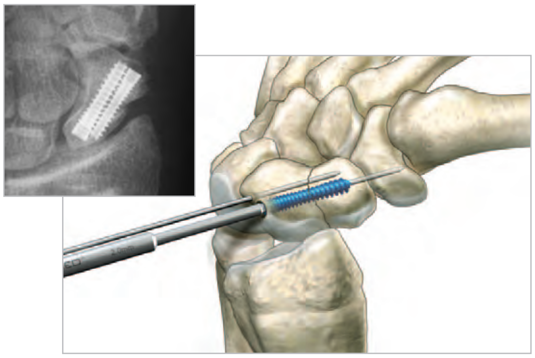

# Jones Fracture Technique: Acutrak 2® - 4.7 and 5.5

**Purpose:** Fracture in hypo-vascular zone of base of fifth metatarsal-"Jones" fracture-Torg 1, 2 and 3. Type 3 fractures may be bone grafted internally prior to screw insertion.

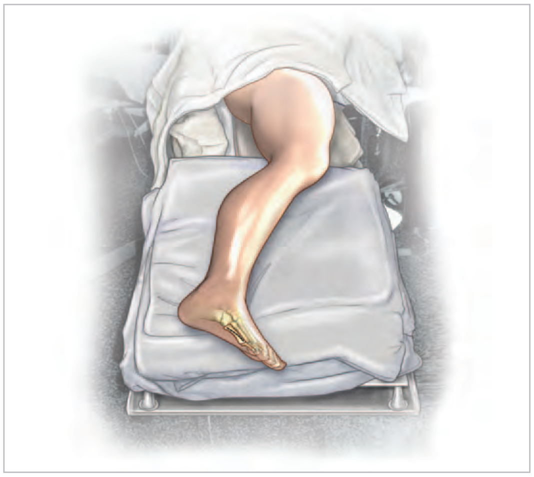

**1** Position the patient in a semi-lateral position utilizing a bean bag body positioner. The patient should be moved to the distal end of the bed and the operative leg draped free as the side up. Exertion of the operative limb should be checked prior to prep and drape to confirm that the operative limb can be positioned on the mini c-arm during surgery.

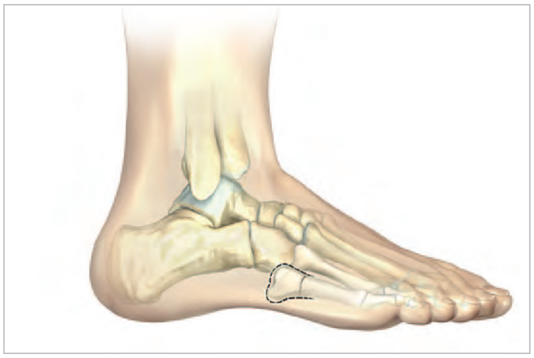

**2** The base of the fifth metatarsal is outlined, including the insertions of the peroneus brevis and tertius tendons.

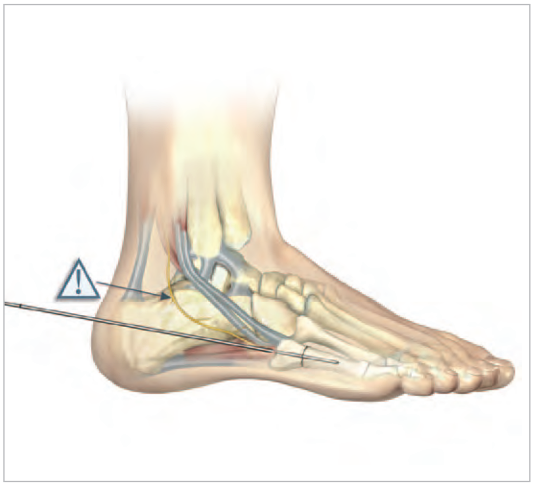

**3** The guide wire, .062", for the Acutrak 2® - 4.7 screw can be positioned at the base of the fifth metatarsal under fluoroscopic guidance. A small incision is made at the base of the fifth metatarsal at the intersection of the peroneus brevis and tertius tendons. ⚠ Care is made to identify and protect the sural nerve branches which run over the peroneal tendons. If necessary, fibers of the lateral aponeurosis and peroneus brevis tendon are separated and retracted away from the styloid process of the base of the fifth metatarsal. A mini Hohman retractor is placed on the plantar aspect of the base of the fifth metatarsal. The surgeon's fingers can be used to reduce the fifth metatarsal fracture by placing them in between the fourth and fifth metarsals. This closes down the fifth metatarsal fracture site during guide wire, drill and screw placement. A guide wire is drilled from the base of the fifth metatarsal into the central portion of the metatarsal shaft. It is maintained within the intramedullary canal in order to avoid distal penetration. Confirm placement with fluoroscopy.

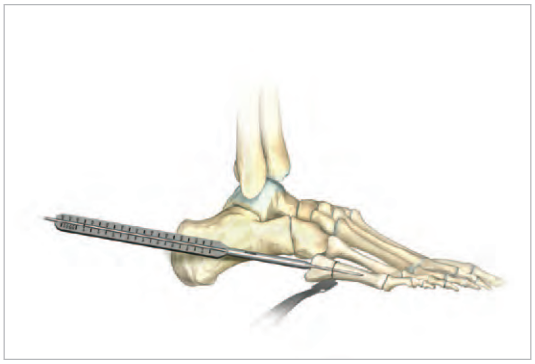

**4** Depth is measured from the exposed portion of the guide wire with the cannulated depth gauge.

**5** After selecting the size, advance the guide wire approximately 5 mm to maintain distal pin fixation before drilling.

**Caution:** Make sure not to compromise distal joint surfaces when advancing the guide wire.

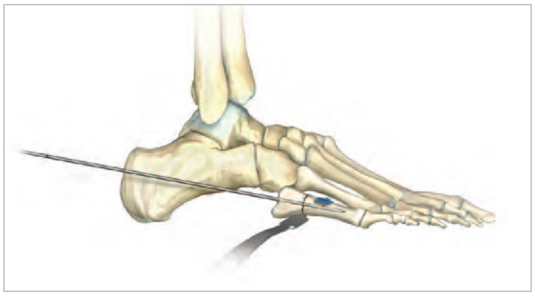

**6** Place the soft tissue guide (the guide should be used throughout) over the guide wire and open the near cortex using the appropriate cannulated profile drill.

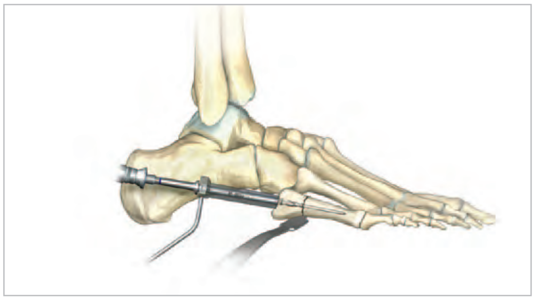

**7** Leaving the soft tissue guide in place, drill into the far fragment with the appropriate cannulated, long drill. Reference the markings on the drill to confirm desired depth.

**Tip:** The long drill is recommended to mitigate the effects of varying bone density and distraction upon screw insertion.

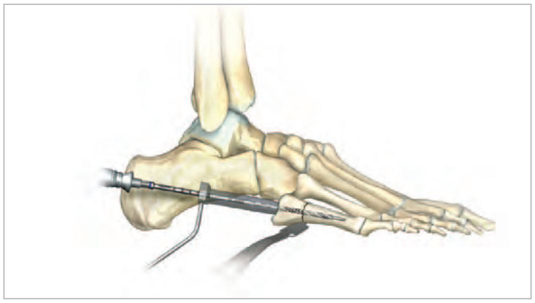

**8** In order to account for countersinking and fracture compression, a screw that measures 5 mm shorter than the measured total depth is characteristically inserted over the guide wire while protecting the soft tissues with a soft tissue guide.

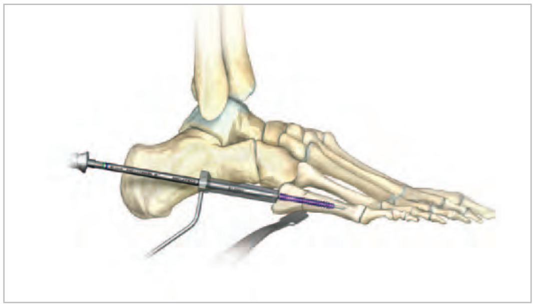

**9** The screw is placed while under fluoroscopic guidance in order to avoid cortical penetration.

**Post-op protocol:** The patient is placed into a soft dressing, supported by a fiberglass splint.

Patients can be made non-weight bearing for a period of 2-6 weeks post-operatively depending upon Torg type of fracture, bone quality and underlying morbidities.

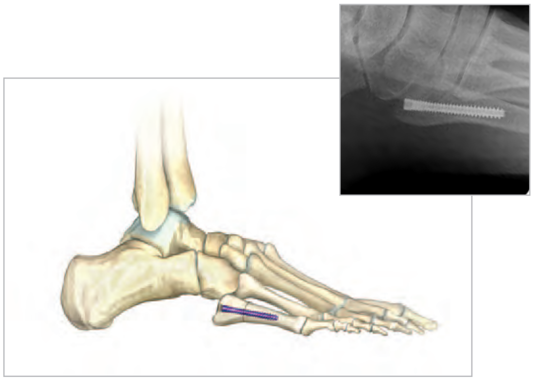

# Calcaneal Osteotomy Technique: Acutrak 2® - 7.5

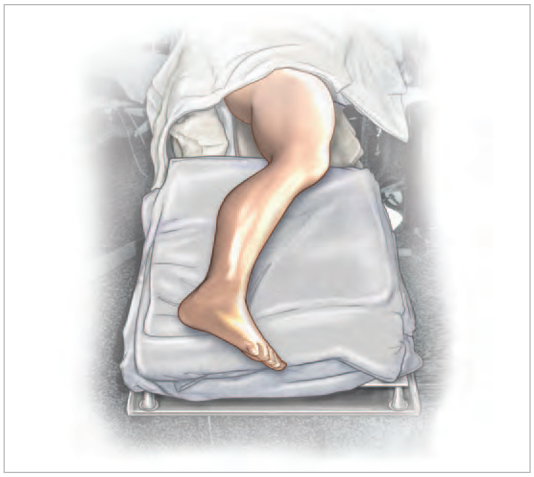

**Purpose:** Medial displacing calcaneal osteotomies are frequently performed to correct hindfoot valgus deformity. Lateral displacing calcaneal osteotomies are performed in patients with a cavus foot arising from the hindfoot. The plantar fascia must be released through a separate incision medially for a lateral displacing osteotomy, whereas the abductor hallucis muscle and medial neurovascular structures can be pushed away through the osteotomy bluntly prior to medial displacement of the osteotomized calcaneus.

1 Position the patient at the end of the bed, semi-lateral. Check that the leg can be placed easily onto the mini-c-arm prior to preparation of the operative limb.

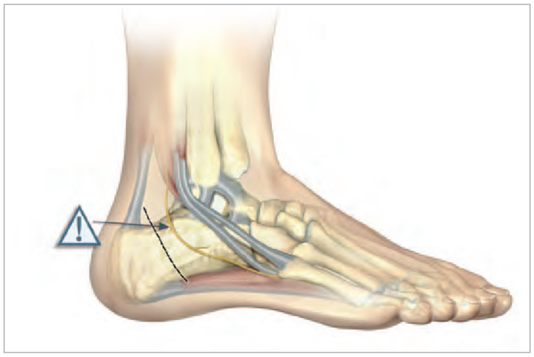

2 An incision is made posterior to the peroneal tendons, perpendicular to the body of the calcaneus. Cephalad and caudal mini hohman retractors are placed to protect the neurovascular structures and plantar fascia. ⚠ Care is made to preserve the peroneal tendons and the sural nerve.

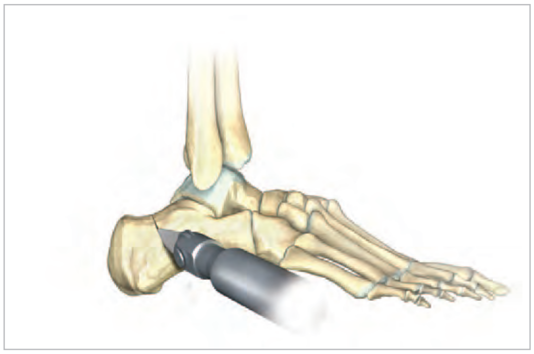

3 An oscillating saw is used to make the osteotomy cut perpendicular to the body of the calcaneus. The saw is not used to complete the cut through the medial cortex. This is completed with an osteotome in order to avoid damaging medial neurovascular structures.

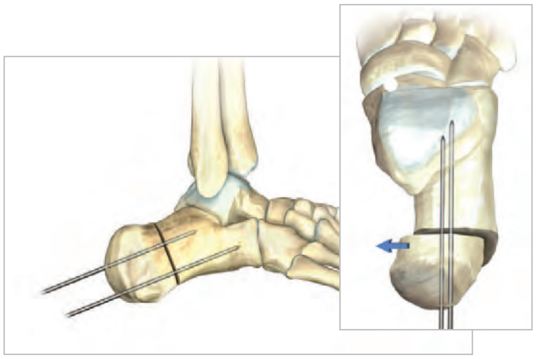

4 The body of the calcaneus is displaced medially or laterally and held in place with two guide pins.

The distal portion of the pins are placed at the volar aspect of the angle of gissane in order to capture solid bone distally and assist with compression of the osteotomy by the screws. Confirm guide pin placement under fluoroscopy.

**Tip:** The soft tissue protector and arthroscopic probe can be used to assist in guide wire placement.

**5** Place the cannulated depth gauge over the guide wire and seat the tip of the depth gauge on the bone. Depth is measured by reading where the laser mark on the guide wire lines up on the depth gauge.

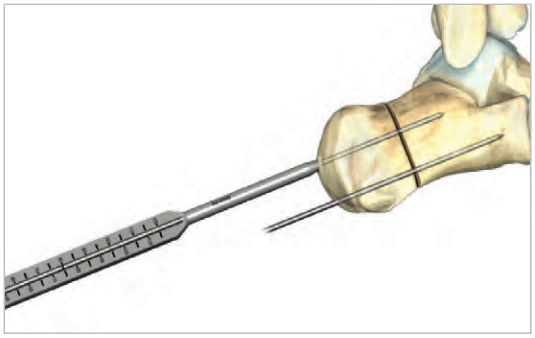

**6** In order to account for countersinking and fracture compression, select a screw one size shorter than the measured total depth.

After selecting the size, advance the guide wire approximately 5 mm to maintain distal pin fixataion before drilling.

**Caution:** Make sure not to compromise joint surfaces when advancing the guide wire.

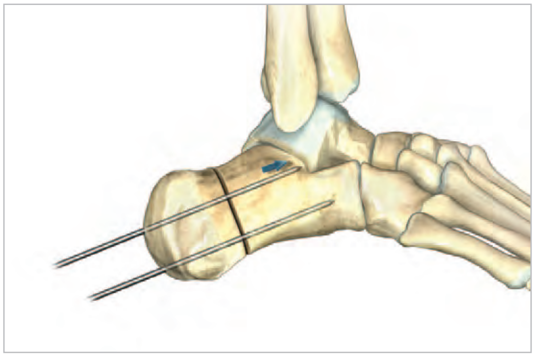

**7** Place the soft tissue guide over the guide wire and open the near cortex using the appropriate cannulated profile drill.

**Tip:** Drills 80-0945, 80-0946, 80-0976 should be advanced slowly with continuous irrigation to reduce the potential of heat build-up. Clean drill periodically during each procedure to optimize performance.

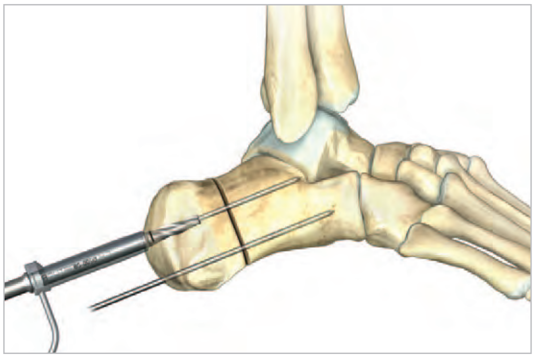

**8** Leaving the soft tissue guide in place, drill into the far fragment with the appropriate cannulated, long profile drill. Reference the markings on the drill to confirm desired depth.

**Tip:** the long drill is recommended to mitigate the effects of varying bone density and distraction upon screw insertion.

**Note:** the Acutrak 2® - 7.5 Long Drill does not show depth markings relative to the bone surface.

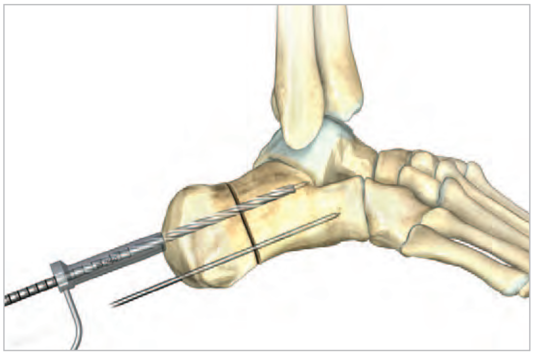

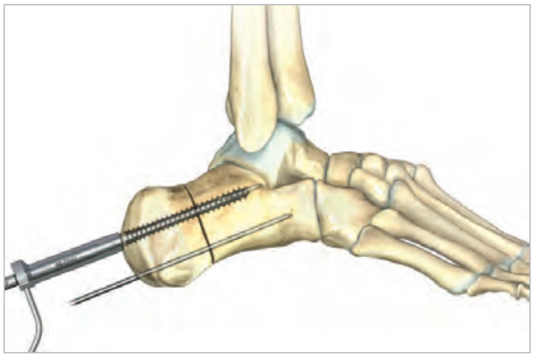

**9** Cannulated Acutrak 2® screws are placed. Placement is confirmed by lateral and axial fluoroscopy views in the operating room.

**Caution:** The marking on the driver shows when the screw is approximately flush with the end of the soft tissue protector; assure that the soft tissue protector is touching bone to accurately determine screw depth. Verify final screw position with fluoroscopy.

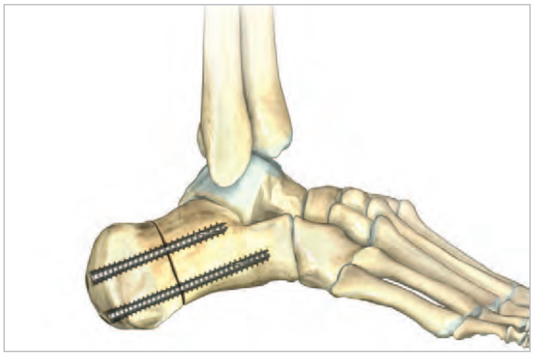

**10** Repeat steps 5-9 for each additional screw placement.

The operative limb is placed into a bulky compression dressing. A splint is also placed. Patients are made non-weight bearing in a cast, boot or splint for 6 weeks after surgery.

**Caution:** Bone density has a great effect on the performance of drills. Peck drilling with long drills is advised.

Drills 80-0945, 80-0946, 80-0976 should be advanced slowly with continuous irrigation to reduce the potential of heat build-up. Clean drill periodically during each procedure to optimize performance.

# Non-Sterile Screw Tray Overview

The Acutrak 2® Instrumentation is available in two modular formats, 1) Micro, Mini, Standard, and 2) 4.7, 5.5, 7.5. All screws available in Non Sterile and Sterile packaging. See ordering information for more details.

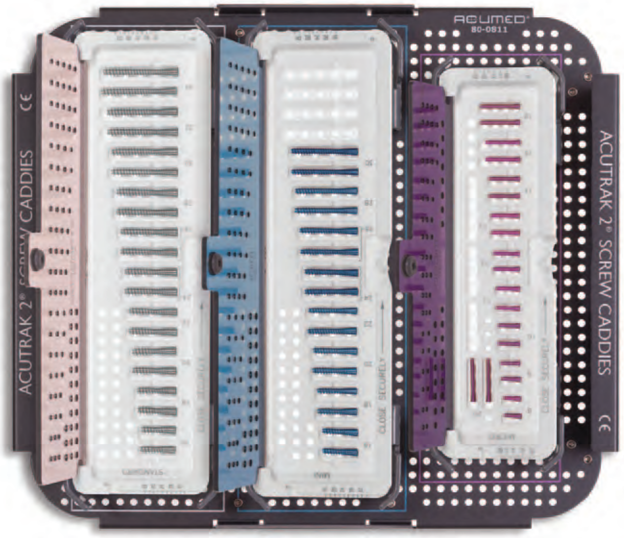


Standard, Mini and Micro Screw Tray

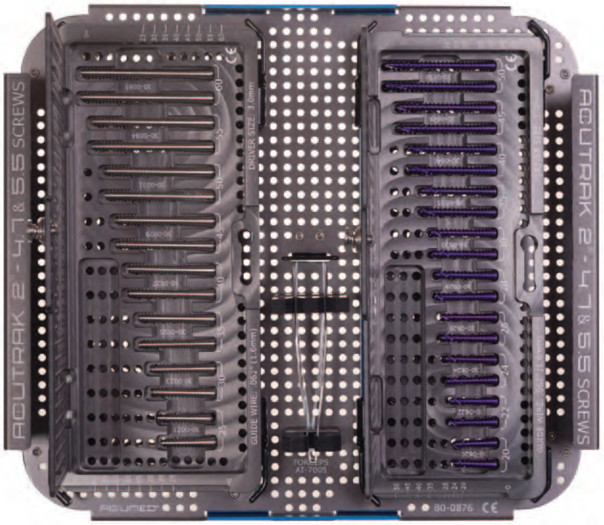


Acutrak 2® - 4.7 & 5.5 Screw Tray

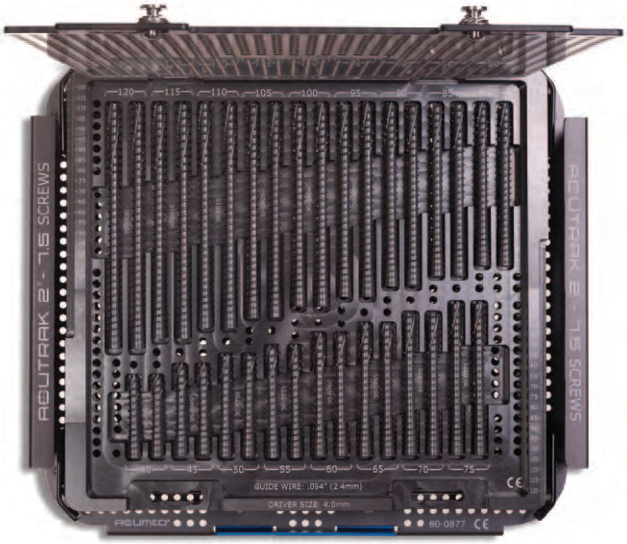


Acutrak 2® - 7.5 Screw Tray

# Ordering Information

### Acutrak 2® Micro Implants

| | |
|---|---|
| Sterile 8 mm Micro Acutrak 2® | AT2-C08-S |
| Sterile 9 mm Micro Acutrak 2® | AT2-C09-S |
| Sterile 10 mm Micro Acutrak 2® | AT2-C10-S |
| Sterile 11 mm Micro Acutrak 2® | AT2-C11-S |
| Sterile 12 mm Micro Acutrak 2® | AT2-C12-S |
| Sterile 13 mm Micro Acutrak 2® | AT2-C13-S |
| Sterile 14 mm Micro Acutrak 2® | AT2-C14-S |
| Sterile 16 mm Micro Acutrak 2® | AT2-C16-S |
| Sterile 18 mm Micro Acutrak 2® | AT2-C18-S |
| Sterile 20 mm Micro Acutrak 2® | AT2-C20-S |
| Non-Sterile 8 mm Micro Acutrak 2® | AT2-C08 |
| Non-Sterile 9 mm Micro Acutrak 2® | AT2-C09 |
| Non-Sterile 10 mm Micro Acutrak 2® | AT2-C10 |
| Non-Sterile 11 mm Micro Acutrak 2® | AT2-C11 |
| Non-Sterile 12 mm Micro Acutrak 2® | AT2-C12 |
| Non-Sterile 13 mm Micro Acutrak 2® | AT2-C13 |
| Non-Sterile 14 mm Micro Acutrak 2® | AT2-C14 |
| Non-Sterile 16 mm Micro Acutrak 2® | AT2-C16 |
| Non-Sterile 18 mm Micro Acutrak 2® | AT2-C18 |
| Non-Sterile 20 mm Micro Acutrak 2® | AT2-C20 |

### Acutrak 2® Micro Instruments

| | |
|---|---|
| Micro Acutrak 2® Parallel Wire Guide Assy | AT2-3500 |
| .035" X 6.0" K-Wire | WS-0906ST |
| Micro Acutrak 2® Profile Drill | AT2-1509 |
| Micro Acutrak 2® Long Profile Drill | 80-0100 |
| 1.5 mm Cannulated Hex Driver | HT-0915 |
| Acutrak 2® Micro X-Ray Temp | ACT70-02 |

### Additional Standard, Mini And Micro Instruments

| | |
|---|---|
| Arthroscopic Cannula Assembly | 80-0519 |
| Acutrak 2®- Arthroscopic Probe | AT2-0402 |
| Acutrak 2® Perc. Screw Sizer (Std, Mini, Micro) | AT2-SMCZ |
| Plunger Assembly | AT-7060 |

### Acutrak 2® Mini Implants

| | |
|---|---|
| Sterile 16 mm Mini Acutrak 2® | AT2-M16-S |
| Sterile 18 mm Mini Acutrak 2® | AT2-M18-S |
| Sterile 20 mm Mini Acutrak 2® | AT2-M20-S |
| Sterile 22 mm Mini Acutrak 2® | AT2-M22-S |
| Sterile 24 mm Mini Acutrak 2® | AT2-M24-S |
| Sterile 26 mm Mini Acutrak 2® | AT2-M26-S |
| Sterile 28 mm Mini Acutrak 2® | AT2-M28-S |
| Sterile 30 mm Mini Acutrak 2® | AT2-M30-S |
| Non-Sterile 16 mm Mini Acutrak 2® | AT2-M16 |
| Non-Sterile 18 mm Mini Acutrak 2® | AT2-M18 |
| Non-Sterile 20 mm Mini Acutrak 2® | AT2-M20 |
| Non-Sterile 22 mm Mini Acutrak 2® | AT2-M22 |
| Non-Sterile 24 mm Mini Acutrak 2® | AT2-M24 |
| Non-Sterile 26 mm Mini Acutrak 2® | AT2-M26 |
| Non-Sterile 28 mm Mini Acutrak 2® | AT2-M28 |
| Non-Sterile 30 mm Mini Acutrak 2® | AT2-M30 |

### Acutrak 2® Mini Instruments

| | |
|---|---|
| Mini Acutrak 2® Parallel Wire Guide Assy | AT2-4500 |
| .045" x 6.0" K-Wire | WS-1106ST |
| Mini Acutrak 2® Profile Drill | AT2M-1813 |
| Mini Acutrak 2® Long Drill | AT2M-L1813 |
| 2.0 mm Cannulated Hex Driver | HT-1120 |
| Acutrak 2® Mini X-ray Template | ACT70-03 |

### Acutrak 2® Standard Implants

| | |
|---|---|
| Sterile 16 mm Standard Acutrak 2® | AT2-S16-S |
| Sterile 18 mm Standard Acutrak 2® | AT2-S18-S |
| Sterile 20 mm Standard Acutrak 2® | AT2-S20-S |
| Sterile 22 mm Standard Acutrak 2® | AT2-S22-S |
| Sterile 24 mm Standard Acutrak 2® | AT2-S24-S |
| Sterile 26 mm Standard Acutrak 2® | AT2-S26-S |
| Sterile 28 mm Standard Acutrak 2® | AT2-S28-S |
| Sterile 30 mm Standard Acutrak 2® | AT2-S30-S |
| Sterile 32 mm Standard Acutrak 2® | AT2-S32-S |
| Sterile 34 mm Standard Acutrak 2® | AT2-S34-S |
| Non-Sterile 16 mm Standard Acutrak 2® | AT2-S16 |
| Non-Sterile 18 mm Standard Acutrak 2® | AT2-S18 |
| Non-Sterile 20 mm Standard Acutrak 2® | AT2-S20 |
| Non-Sterile 22 mm Standard Acutrak 2® | AT2-S22 |
| Non-Sterile 24 mm Standard Acutrak 2® | AT2-S24 |
| Non-Sterile 26 mm Standard Acutrak 2® | AT2-S26 |
| Non-Sterile 28 mm Standard Acutrak 2® | AT2-S28 |
| Non-Sterile 30 mm Standard Acutrak 2® | AT2-S30 |
| Non-Sterile 32 mm Standard Acutrak 2® | AT2-S32 |
| Non-Sterile 34 mm Standard Acutrak 2® | AT2-S34 |

### Acutrak 2® Standard Instruments

| | |
|---|---|
| Standard Acutrak 2® Parallel Wire Guide Assy | AT2-5400 |
| .054" x 7.0" K-Wire | WS-1407ST |
| Standard Acutrak 2® Profile Drill | AT2-2515 |
| Standard Acutrak 2® Long Drill | AT2-L2515 |
| 2.5 mm Cannulated Hex Driver | HT-1725 |
| Acutrak 2® STD X-ray Temp | ACT70-01 |

### Universal Platter Standard, Mini and Micro Tray Additional Inst.

| | |
|---|---|
| 1.5 mm Easyout, QR | 80-0598 |
| 2.0 mm Easyout, QR | 80-0599 |
| 2.5 mm Easyout, QR | 80-0600 |
| Medium Ratcheting Driver Handle | 80-0663 |
| 6 mm Graft Removal Paddle Assembly | BG-8064 |
| 7 mm Bone Graft Drill Assembly | PL-BG07 |
| Universal Acutrak 2® Std, Mini & Micro Instrument Base | 80-0808 |
| Universal Acutrak 2® Std, Mini & Micro Instrument Platter Lid | 80-0809 |

# Ordering Information

### Acutrak 2® 4.7- Implants

| | |
|---|---|
| Sterile 20.0 mm Acutrak 2® - 4.7 Screw | 30-0620-S |
| Sterile 22.0 mm Acutrak 2® - 4.7 Screw | 30-0622-S |
| Sterile 24.0 mm Acutrak 2® - 4.7 Screw | 30-0624-S |
| Sterile 26.0 mm Acutrak 2® - 4.7 Screw | 30-0626-S |
| Sterile 28.0 mm Acutrak 2® - 4.7 Screw | 30-0628-S |
| Sterile 30.0 mm Acutrak 2® - 4.7 Screw | 30-0630-S |
| Sterile 35.0 mm Acutrak 2® - 4.7 Screw | 30-0635-S |
| Sterile 40.0 mm Acutrak 2® - 4.7 Screw | 30-0640-S |
| Sterile 45.0 mm Acutrak 2® - 4.7 Screw | 30-0645-S |
| Sterile 50.0 mm Acutrak 2® - 4.7 Screw | 30-0650-S |
| Non-Sterile 20.0 mm Acutrak 2® - 4.7 Screw | 30-0620 |
| Non-Sterile 22.0 mm Acutrak 2® - 4.7 Screw | 30-0622 |
| Non-Sterile 24.0 mm Acutrak 2® - 4.7 Screw | 30-0624 |
| Non-Sterile 26.0 mm Acutrak 2® - 4.7 Screw | 30-0626 |
| Non-Sterile 28.0 mm Acutrak 2® - 4.7 Screw | 30-0628 |
| Non-Sterile 30.0 mm Acutrak 2® - 4.7 Screw | 30-0630 |
| Non-Sterile 35.0 mm Acutrak 2® - 4.7 Screw | 30-0635 |
| Non-Sterile 40.0 mm Acutrak 2® - 4.7 Screw | 30-0640 |
| Non-Sterile 45.0 mm Acutrak 2® - 4.7 Screw | 30-0645 |
| Non-Sterile 50.0 mm Acutrak 2® - 4.7 Screw | 30-0650 |

### Acutrak 2® 4.7 Instruments

| | |
|---|---|
| Acutrak 2® - 4.7 Profile Drill | 80-0945 |
| Acutrak 2® - 4.7 Long Drill | 80-0946 |

### Acutrak 2® - 4.7 and 5.5 Instruments

| | |
|---|---|
| 1.6 mm Wire Probe | 80-0992 |
| 1.6mm (.062") x 9.25" GUIDE WIRE | 80-0950 |
| Cannulated Driver, 3.0 mm Acutrak 2® | 80-0958 |
| Solid Driver, 3.0 mm Acutrak 2® | 80-0959 |

### Additional Acutrak 2® - 4.7, 5.5 and 7.5 Instruments

| | |
|---|---|
| Soft Tissue Protector | 80-0990 |
| Large Acutrak 2® Screw Sizer | 80-0996 |
| Plunger | AT-7060 |

### Acutrak 2® 5.5 - Implants

| | |
|---|---|
| Sterile 25 mm Acutrak 2® - 5.5 Screw | 30-0021-S |
| Sterile 30 mm Acutrak 2® - 5.5 Screw | 30-0023-S |
| Sterile 35 mm Acutrak 2® - 5.5 Screw | 30-0025-S |
| Sterile 40 mm Acutrak 2® - 5.5 Screw | 30-0027-S |
| Sterile 45 mm Acutrak 2® - 5.5 Screw | 30-0029-S |
| Sterile 50 mm Acutrak 2® - 5.5 Screw | 30-0031-S |
| Sterile 55 mm Acutrak 2® - 5.5 Screw | 30-0084-S |
| Sterile 60 mm Acutrak 2® - 5.5 Screw | 30-0085-S |
| Non-Sterile 25 mm Acutrak 2® - 5.5 Screw | 30-0021 |
| Non-Sterile 30 mm Acutrak 2® - 5.5 Screw | 30-0023 |
| Non-Sterile 35 mm Acutrak 2® - 5.5 Screw | 30-0025 |
| Non-Sterile 40 mm Acutrak 2® - 5.5 Screw | 30-0027 |
| Non-Sterile 45 mm Acutrak 2® - 5.5 Screw | 30-0029 |
| Non-Sterile 50 mm Acutrak 2® - 5.5 Screw | 30-0031 |
| Non-Sterile 55 mm Acutrak 2® - 5.5 Screw | 30-0084 |
| Non-Sterile 60 mm Acutrak 2® - 5.5 Screw | 30-0085 |

### Acutrak 2® 5.5 Instruments

| | |
|---|---|
| Acutrak 2® 5.5 Profile Drill | 80-0955 |
| Acutrak 2® 5.5 Long Drill | 80-0956 |

## Acutrak 2® 7.5- Implants

| | |
|---|---|
| Sterile 40 mm Acutrak 2® - 7.5 Screw | 30-0740-S |
| Sterile 45 mm Acutrak 2® - 7.5 Screw | 30-0745-S |
| Sterile 50 mm Acutrak 2® - 7.5 Screw | 30-0750-S |
| Sterile 55 mm Acutrak 2® - 7.5 Screw | 30-0755-S |
| Sterile 60 mm Acutrak 2® - 7.5 Screw | 30-0760-S |
| Sterile 65 mm Acutrak 2® - 7.5 Screw | 30-0765-S |
| Sterile 70 mm Acutrak 2® - 7.5 Screw | 30-0770-S |
| Sterile 75 mm Acutrak 2® - 7.5 Screw | 30-0775-S |
| Sterile 80 mm Acutrak 2® - 7.5 Screw | 30-0780-S |
| Sterile 85 mm Acutrak 2® - 7.5 Screw | 30-0785-S |
| Sterile 90 mm Acutrak 2® - 7.5 Screw | 30-0790-S |
| Sterile 95 mm Acutrak 2® - 7.5 Screw | 30-0795-S |
| Sterile 100 mm Acutrak 2® - 7.5 Screw | 30-0800-S |
| Sterile 105 mm Acutrak 2® - 7.5 Screw | 30-0805-S |
| Sterile 110 mm Acutrak 2® - 7.5 Screw | 30-0810-S |
| Sterile 115 mm Acutrak 2® - 7.5 Screw | 30-0815-S |
| Sterile 120 mm Acutrak 2® - 7.5 Screw | 30-0820-S |
| Non-Sterile 40 mm Acutrak 2® - 7.5 Screw | 30-0740 |
| Non-Sterile 45 mm Acutrak 2® - 7.5 Screw | 30-0745 |
| Non-Sterile 50 mm Acutrak 2® - 7.5 Screw | 30-0750 |
| Non-Sterile 55 mm Acutrak 2® - 7.5 Screw | 30-0755 |
| Non-Sterile 60 mm Acutrak 2® - 7.5 Screw | 30-0760 |
| Non-Sterile 65 mm Acutrak 2® - 7.5 Screw | 30-0765 |
| Non-Sterile 70 mm Acutrak 2® - 7.5 Screw | 30-0770 |
| Non-Sterile 75 mm Acutrak 2® - 7.5 Screw | 30-0775 |
| Non-Sterile 80 mm Acutrak 2® - 7.5 Screw | 30-0780 |
| Non-Sterile 85 mm Acutrak 2® - 7.5 Screw | 30-0785 |
| Non-Sterile 90 mm Acutrak 2® - 7.5 Screw | 30-0790 |
| Non-Sterile 95 mm Acutrak 2® - 7.5 Screw | 30-0795 |
| Non-Sterile 100 mm Acutrak 2® - 7.5 Screw | 30-0800 |
| Non-Sterile 105 mm Acutrak 2® - 7.5 Screw | 30-0805 |
| Non-Sterile 110 mm Acutrak 2® - 7.5 Screw | 30-0810 |
| Non-Sterile 115 mm Acutrak 2® - 7.5 Screw | 30-0815 |
| Non-Sterile 120 mm Acutrak 2® - 7.5 Screw | 30-0820 |

## Acutrak 2® 7.5- Instruments

| | |
|---|---|
| 2.4 mm Wire Probe | 80-0994 |
| 2.4 mm (.094") X 9.25" Guide wire | 80-0970 |
| 2.4 mm (.094") X 9.25" Guide wire, threaded | 80-0971 |
| Acutrak 2® - 7.5 Profile drill | 80-0975 |
| Acutrak 2® - 7.5 Long drill | 80-0976 |
| Cannulated Driver, 4.0 mm Acutrak 2® 6" long | 80-0978 |
| Solid Driver, 4.0 mm Acutrak 2 | 80-0979 |

### Universal Platter Acutrak 2® - 4.7, 5.5 and 7.5 Tray Additional Inst.

| | |
|---|---|
| Large Acutrak 2® Drills & Driver Platter | 80-0870 |
| Large Acutrak 2® Common Inst. Platter | 80-0871 |
| A/O Quick Release Handle | 80-0398 |
| Forceps | AT-7005 |
| Ratchet T-Handle with A/O Connection | 80-0999 |
| Sharp Hook | PL-CL06 |
| 3.0 mm Easyout, QR | 80-0601 |
| 4.0 mm Easyout, QR | 80-0603 |

### Universal Platter Acutrak 2® - 4.7, 5.5 and 7.5 Tray Additional Instruments - Sterile

| | |
|---|---|
| Large Acutrak 2® Screw System Lid | 80-0869 |

### Universal Platter Acutrak 2® - 4.7, 5.5 and 7.5 Tray Additional Instruments - Non-Sterile

| | |
|---|---|
| Large Acutrak 2® 4.7 & 5.5 Screw Platter | 80-0876 |
| Large Acutrak 2® 7.5 Screw Platter | 80-0877 |
| Large Acutrak 2® 4.7 Screw Caddy | 80-0878 |
| Large Acutrak 2® 5.5 Screw Caddy | 80-0880 |
| Large Acutrak 2® 7.5 Screw Caddy | 80-0882 |
| Large Acutrak 2® Screw 2x2 Base | 80-0884 |
| Large Acutrak 2® Screw Lid | 80-0885 |

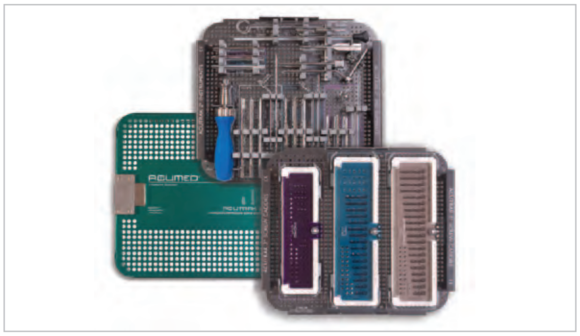

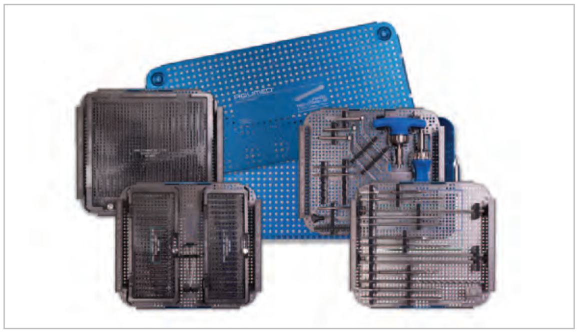

ACUMED®
5885 NW Cornelius Pass Road
Hillsboro, OR 97124
(888) 627-9957
www.acumed.net

Distributed by:

These materials contain information about products that may or may not be available in any particular country or may be available under different trademarks in different countries. The products may be approved or cleared by governmental regulatory organizations for sale or use with different indications or restrictions in different countries. Products may not be approved for use in all countries. Nothing contained on these materials should be construed as a promotion or solicitation for any product or for the use of any product in a particular way which is not authorized under the laws and regulations of the country where the reader is located. Specific questions physicians may have about the availability and use of the products described on these materials should be directed to their particular local sales representative. Specific questions patients may have about the use of the products described in these materials or the appropriateness for their own conditions should be directed to their own physician.

SPF00-02-E
Effective: 05/2012
© 2012 Acumed® LLC
