# Supplementary material for: Impact of Different Screw Designs on Durability of Fracture Fixation: In Vitro Study with Cyclic Loading of Scaphoid Bones
Source: PLoS One. 2016 Jan 7;11(1):e0145949. doi: 10.1371/journal.pone.0145949 (PMC4704798; doi:10.1371/journal.pone.0145949)
Supplement: S2 Text — (PDF) [file pone.0145949.s005.pdf]

stryker®

TwinFix
Cannulated Compression Screw

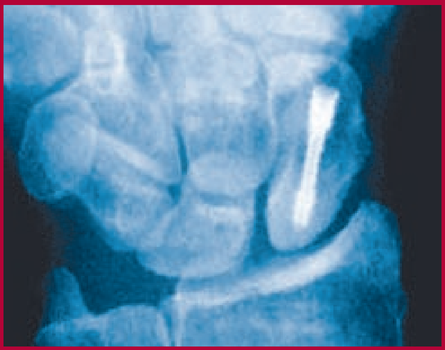

Leibinger Solutions for Hand Surgery

Procedural Guide

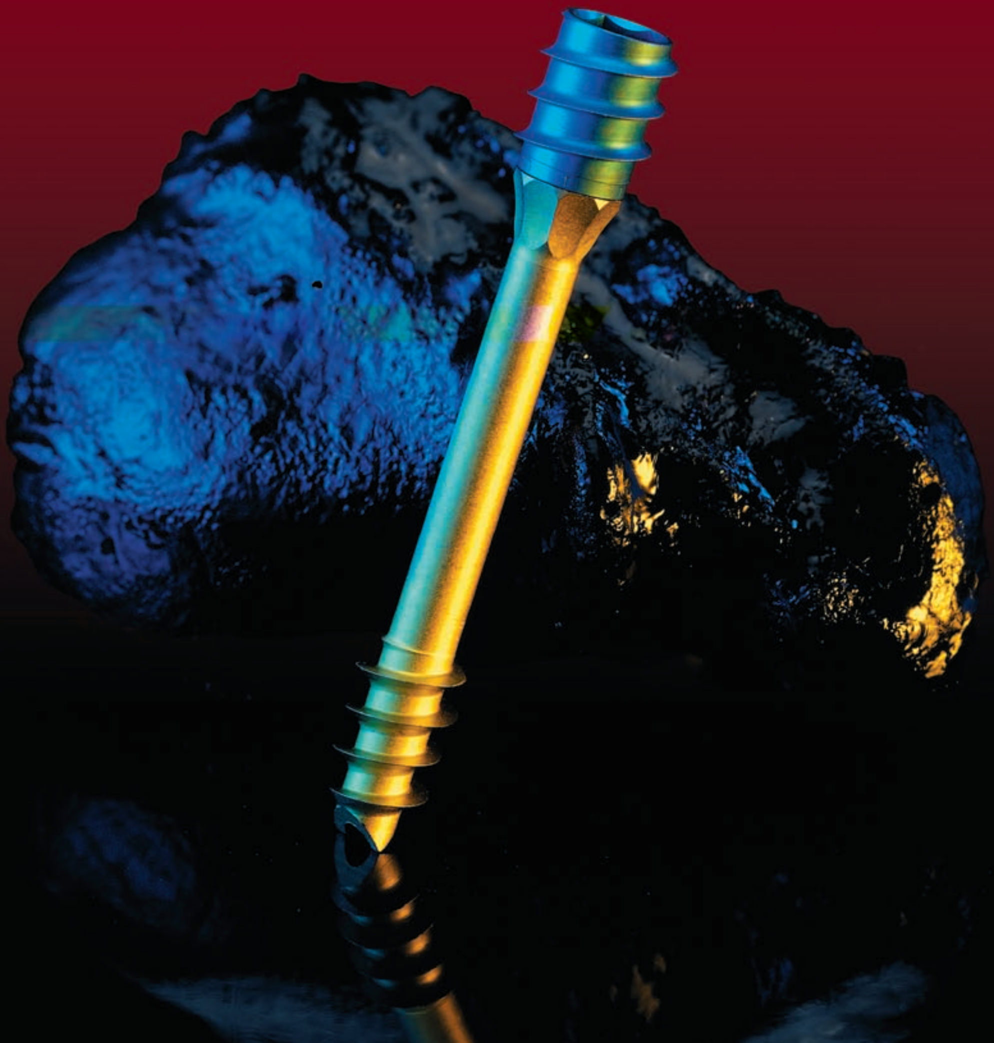

TwinFix

Sterilization, Organization, Storage

**29-12020**	Profyle MODULAR Sterilizing Container
**29-40162**	TwinFix Implant Module
**29-12007**	Instrument Rack
**29-12022**	Rack for Implant Module
**29-12024**	Profyle MODULAR Generic Instrument tray with silicone insert
**29-12021**	Lid for Sterilizing Container

Instrumentation for Percutaneous Approach

**07-40250**	Drill and Screw Guide
**07-40211**	Sleeve for Target Device (07-40210) and Drill and Screw Guide (07-40250)
**07-40215**	Drill Guide for 1 mm K-Wires
**07-40240**	Cannulated Screwdriver Handle
**07-40230**	Cannulated Drill 2.4 mm, with depth stop
**07-40232**	Cannulated tap
**07-40220**	Cannulated Screwdriver Blade
**07-40280**	K-Wire, 1.0 x 160 mm (Package of 10)
**07-40245**	Depth Measuring Gauge

Additional Instrumentation for Open Screw Osteosynthesis

**07-40210**	Target Device for Compression Screw
**07-40216**	Scaphoid Target Bow
**07-40270**	Self-retaining Retractor with Guiding Sleeves for Kirschner Wires 1.0/1.6 mm diam.

Optional

**07-40221**	Emergency blade for screw removal

3.2 mm TwinFix Cannulated Compression Screws

**58-30414**	Diam. 3.2 x 14 mm
**58-30416**	Diam. 3.2 x 16 mm
**58-30418**	Diam. 3.2 x 18 mm
**58-30420**	Diam. 3.2 x 20 mm
**58-30421**	Diam. 3.2 x 21 mm
**58-30422**	Diam. 3.2 x 22 mm
**58-30423**	Diam. 3.2 x 23 mm
**58-30424**	Diam. 3.2 x 24 mm
**58-30425**	Diam. 3.2 x 25 mm
**58-30426**	Diam. 3.2 x 26 mm
**58-30427**	Diam. 3.2 x 27 mm
**58-30428**	Diam. 3.2 x 28 mm
**58-30430**	Diam. 3.2 x 30 mm
**58-30432**	Diam. 3.2 x 32 mm
**58-30434**	Diam. 3.2 x 34 mm

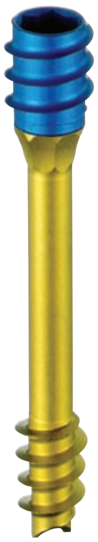

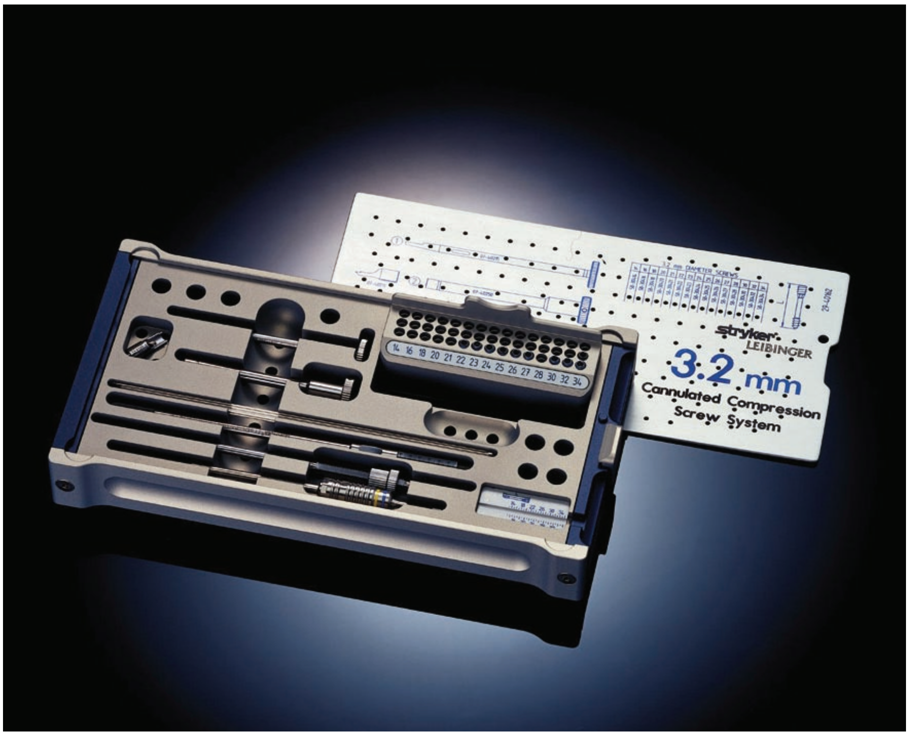

Indications

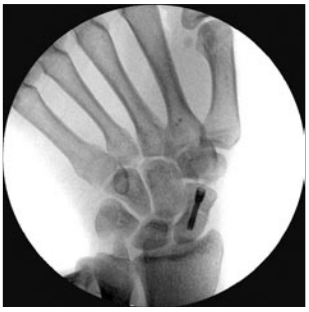

Scaphoid fracture

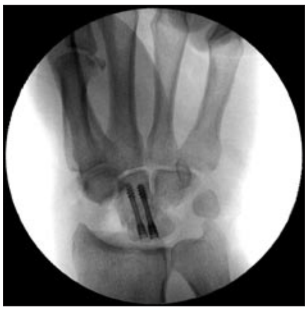

Intercarpal arthrodesis

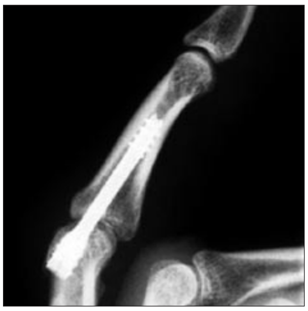

Finger joint arthrodesis

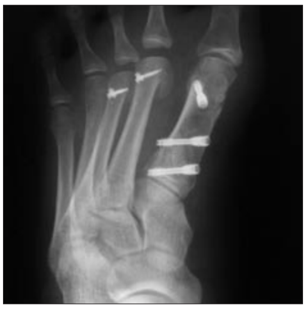

Corrective osteotomy of hallux valgus

Treatment of a scaphoid fracture

1. Insertion of the K-wire

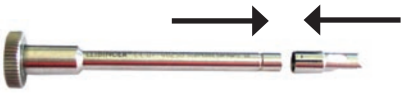

Drill and Screw Guide Sleeve

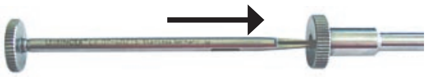

Drill Guide for K-wire Drill and Screw Guide

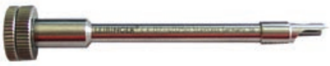

Completely assembled

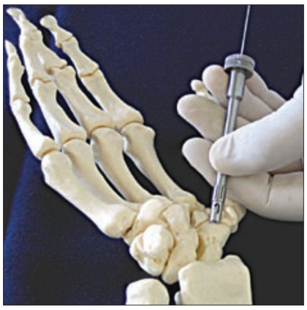

The K-wire is inserted using the drill and screw guide.

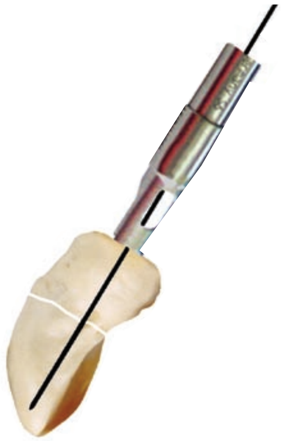

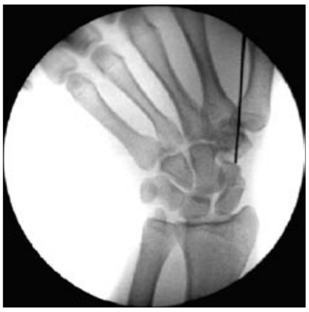

Maximum ulnar inclination of the wrist provides a good view of the scaphoid.

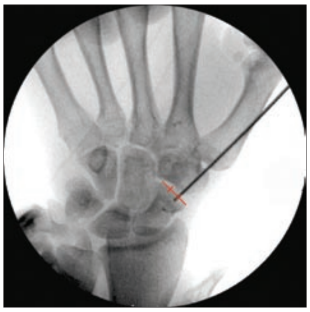

Under image intensifier, the K-wire is applied in p.a. projection at the junction of the radial/middle third of the scaphoid tubercle. Ensure that insertion is as far dorsally as possible on the edge of the trapezium.

The wire is then drilled proximally under repeated image intensifier control towards the „tip of the scaphoid“.

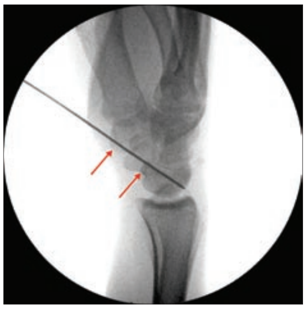

On the lateral projection, the K-wire must not penetrate the waist of the scaphoid. It should lie within the bone at a distance of approx. 2 mm from the palmar cortex.

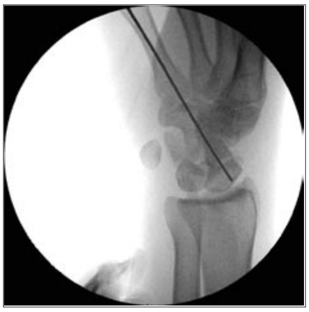

With the hand in 45° supination, the proximal pole of the scaphoid is seen very clearly. The tip of the wire is drilled forward in this position as far as the cortex but without penetrating it.
The position of the wire is then checked again carefully in all 4 planes.

This Step-by-Step Procedure has been developed in cooperation with Dr. Hans-Werner Bouman, ATOS Praxisklinik, Bismarckstraße 9-15, D-69115 Heidelberg.

2. Checking the position of the Kirschner wire in 4 planes under image intensifier control

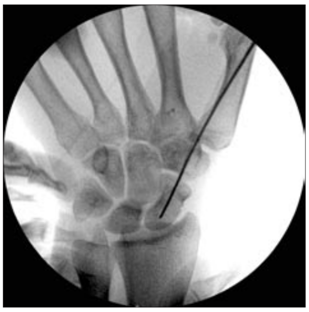

a.p. (axial alignment of the K-wire in the scaphoid)

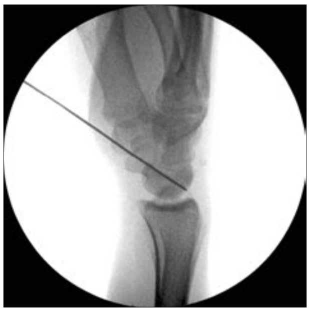

lateral (position relative to the trapezium and to the waist of the scaphoid)

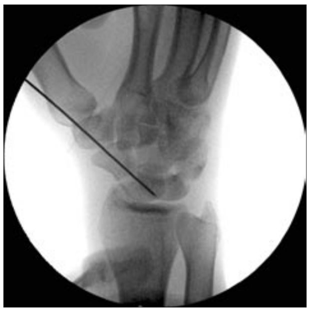

45° pronation (view of the head of the scaphoid)

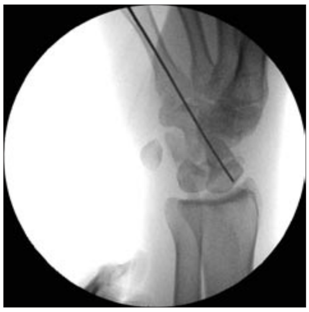

45° supination (view of the pole of the scaphoid)

---

3. Determining screw length

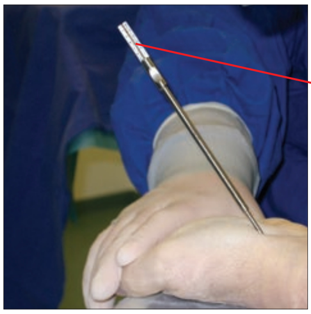

Measuring the length - the measuring sleeve is inserted over the K-wire. **Ensure that the tip sits firmly on the tubercle and that no soft tissue has become interposed.**

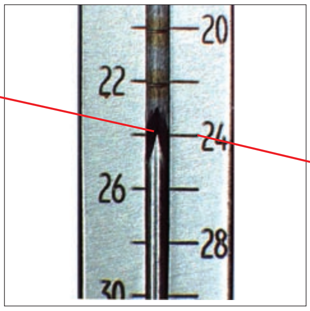

Reading the scale – the end of the K-wire defines the length of the screw.

Caution: for reasons of safety, the scale shows 2 mm less than the actual length of the wire.

4. Drilling through drill and screw guide

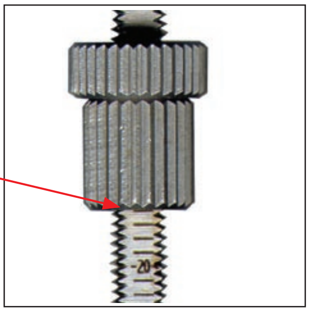

The length read off the depth measuring gauge is set on the cannulated drill by means of the knurled screw.

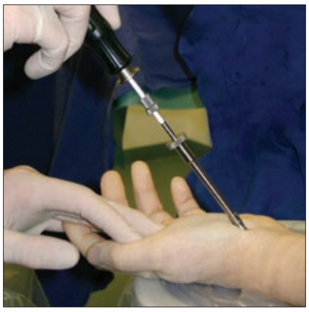

The drill guide with sleeve is positioned firmly to the tubercle (caution: interposed tissue). The opening on the sleeve faces towards the trapezium. The drill is inserted into the drill guide over the K-wire and is rotated until it meets the knurled nut. Do not change the angulation of the drill while the K-wire is inserted into the drill. Checking the position with the image intensifier is recommended.
Alternatively, drilling can be continued under constant image intensifier control until just before the tip of the K-wire.

---

5. Loading the screw

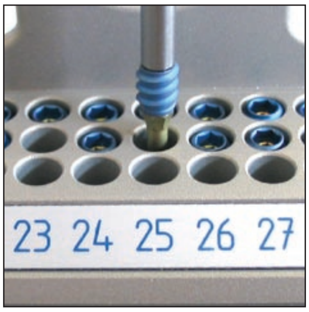

Check the screwdriver blade is in the locked position (yellow and blue ring visible), center the screwdriver blade and press firmly to remove the screw from the implant module.

6. Measuring screw length

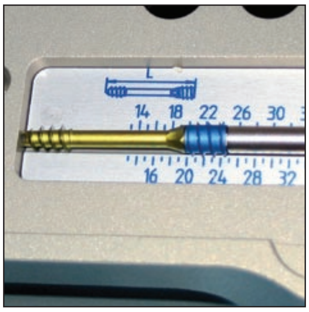

Optional: checking of the screw length on the measuring scale of the implant module.

7. Insertion of the screw

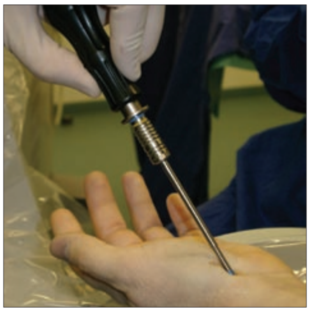

Selection of a screw shorter than the measured length is recommended. This guarantees that the head of the screw can be submerged sufficiently beneath the level of the cortex and the tip of the screw will not impact on the end of the drill hole in the proximal fragment and force the fracture apart.

7. Insertion of the screw

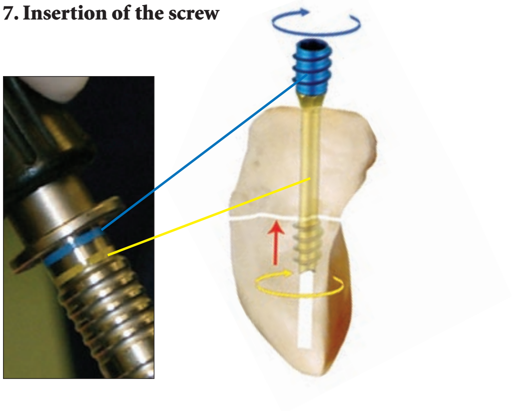

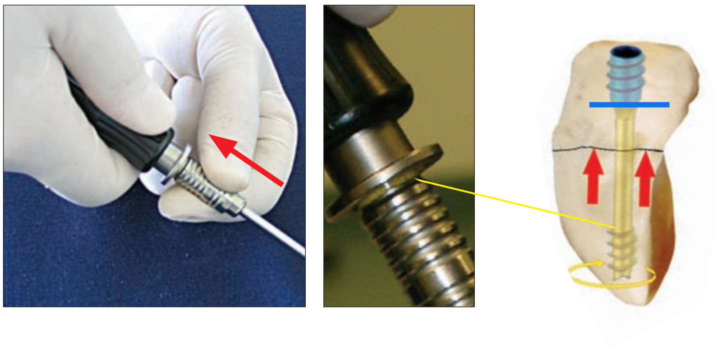

The screwdriver is locked when inserting the screw (coupling pushed completely forward both blue and yellow rings are visible), so that the screw head and screw foot turn simultaneously. When the reamer below the head reaches the cortex (see illustration), a certain degree of precompression of the fragments occurs.

When the screw head is completely submerged in the bone (image intensifier control in 45° pronation), the screwdriver is unlocked. To do this, the sleeve is pulled backward so that only the yellow ring is visible.
Now only the foot thread (gold) can turn. A ¼ rotation is usually sufficient to complete compression.

8. Checking the position of the screw in 4 planes

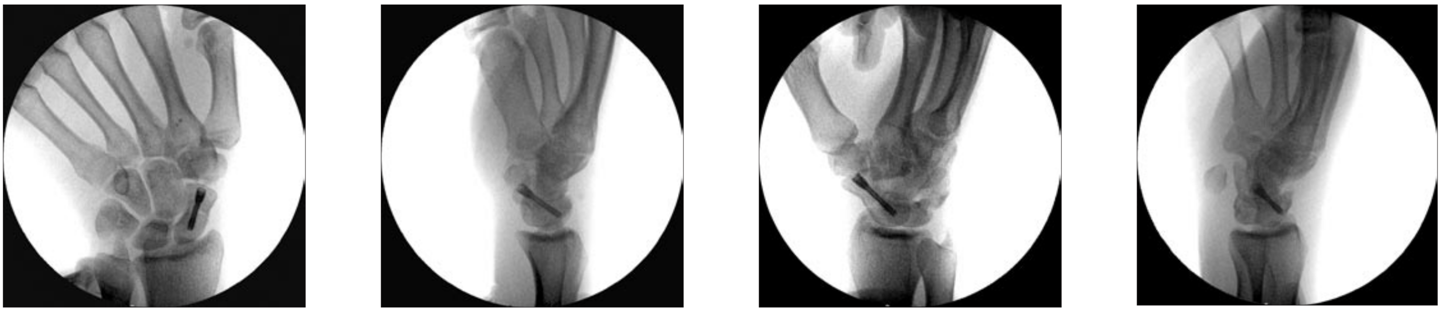

Open treatment of a scaphoid fracture or pseudarthrosis with the target device

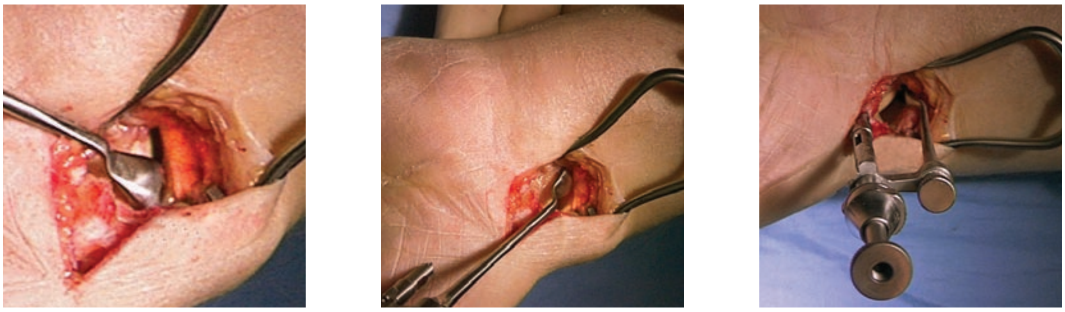

The target bow is introduced between the scaphoid and the styloid process of the radius with the wrist distracted.

The target bow is then pushed downwards. By rotating the target bow slightly, the edge of the spoon slots in between the scaphoid and the lunate and the joint surface of the radius.

The target device is now pressed together firmly so that the teeth of the sleeve attachment hook securely into the radial third of the tubercle. The lateral opening of the sleeve faces towards the trapezium and must lie as close to this as possible.

The required screw length is read from the side of the sleeve on the target device.

Caution:
When measuring with the target device, a safety distance of 4 mm is included so that the TwinFix screw can be submerged sufficiently beneath the level of the cortex.

All further steps are described under points 4 – 8.

For cleaning and sterilization please follow the instructions for use.

stryker®

Joint Replacements

Trauma, Extremities & Deformities

Craniomaxillofacial

Spine

Biologics

Surgical Products

Neuro & ENT

Interventional Pain

Navigation

Endoscopy

Communications

Imaging

Patient Handling Equipment

EMS Equipment

Stryker Leibinger GmbH &Co. KG
Bötzinger Straße 37-41
D-79111 Freiburg
Germany
Tel.: ++49 (0) 7 61 45 12-0
Fax: ++49 (0) 7 61 45 12-120

Stryker Leibinger Inc.
4100 East Milham Avenue
Kalamazoo, MI 49001
USA
Tel.: ++1-269-323-7700
Fax: ++1-269-648-7114

http://www.leibinger.com

The information presented in this brochure is intended to demonstrate a Stryker product. Always refer to the package insert, product label and/or user instructions before using any Stryker product. Products may not be available in all markets. Product availability is subject to the regulatory or medical practices that govern individual markets. Please contact your Stryker representative if you have questions about the availability of Stryker products in your area.

Products referenced with ™ designation are trademarks of Stryker.
Products referenced with ® designation are registered trademarks of Stryker.

Literature Number **90-07542**
Print Date 08/06 Rev. 1

Copyright © 2004 Stryker
