# Supplementary material for: Impact of Different Screw Designs on Durability of Fracture Fixation: In Vitro Study with Cyclic Loading of Scaphoid Bones
Source: PLoS One. 2016 Jan 7;11(1):e0145949. doi: 10.1371/journal.pone.0145949 (PMC4704798; doi:10.1371/journal.pone.0145949)
Supplement: S3 Text — (PDF) [file pone.0145949.s006.pdf]

HCS 2.4/3.0. The countersinkable compression screw.

Technique Guide

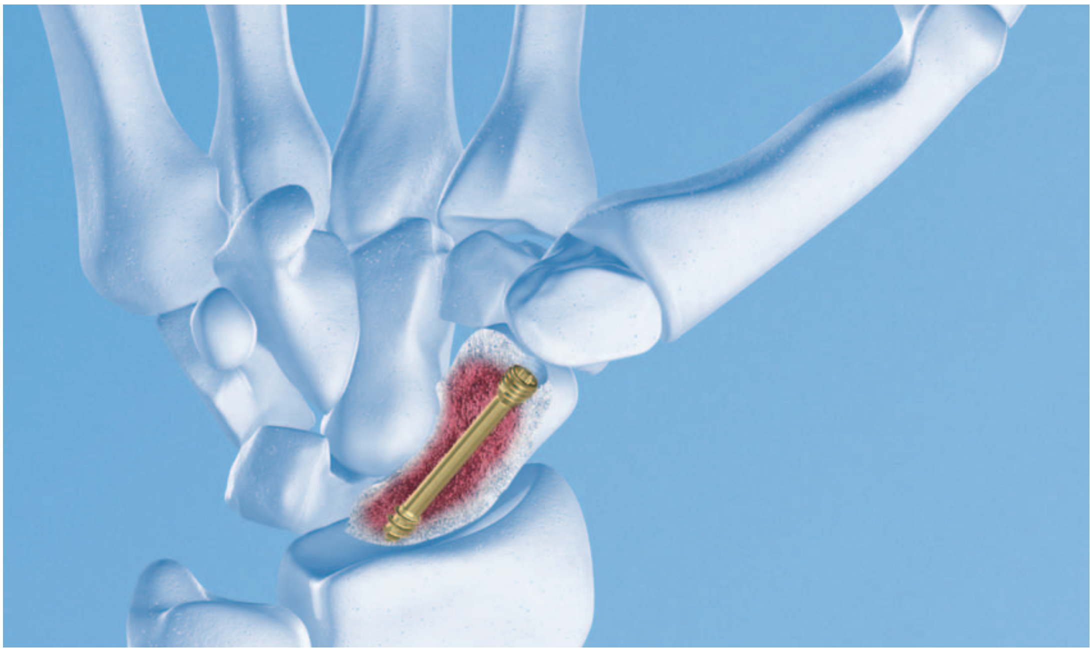

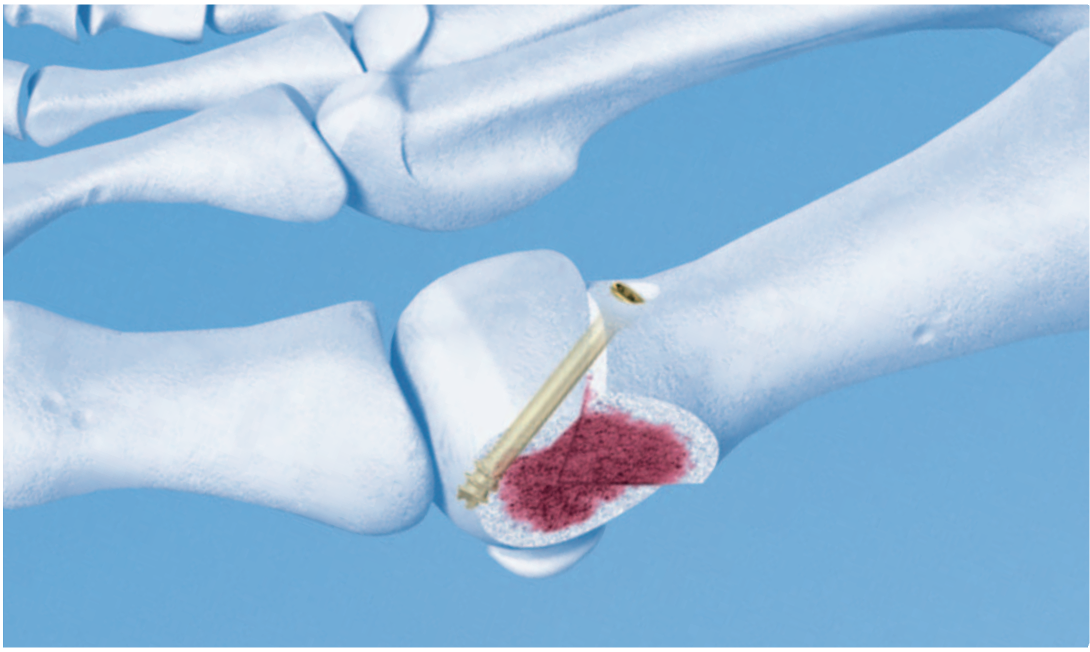

SYNTHES®

Instruments and implants
approved by the AO Foundation

Table of Contents

**Introduction**	Features and Benefits	2
	Functional Principle	3
	Indications	4
**Surgical Technique**	Hand – Scaphoid	5
	Foot – Chevron Osteotomy for Hallux Valgus	14
	Using the Optional Drill Guide with Stop	21
	Screw Extraction	22
**Product Information**	Implants HCS 2.4	23
	Implants HCS 3.0	25
	Instruments HCS 2.4 and 3.0	27
	Optional Instruments for HCS 2.4 and 3.0	29
	Setlists HCS 2.4 and 3.0	30

Image intensifier control

Warning
This description alone does not provide sufficient background for direct use of the instrument set. Instruction by a surgeon experienced in handling these instruments is highly recommended.

Reprocessing, Care and Maintenance of Synthes Instruments
For general guidelines, function control and dismantling of multi-part instruments, please refer to: www.synthes.com/reprocessing

Features and Benefits

Tip with self-drilling and self-tapping flutes
Shorter surgery due to simplified surgical technique

Cannulation
For minimally invasive technique and guided insertion

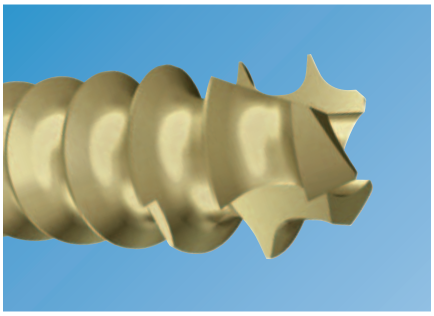

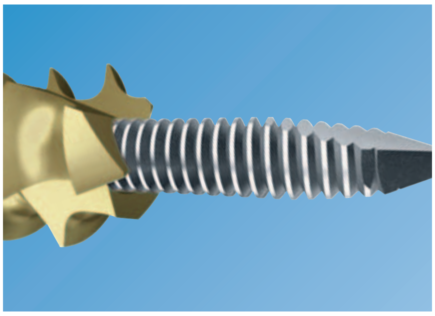

Head with self-tapping flutes
Facilitates countersinking of screw

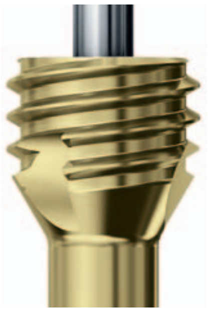

Two different thread lengths of the shaft
The optimal implant for every case due to threads available in different lengths

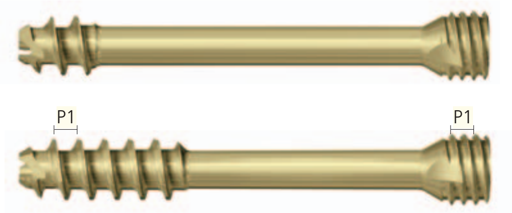

Identical pitch of head and shaft threads
For controlled closure and compression of the fracture gap

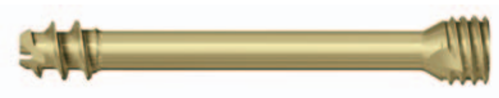

Available in steel and titanium
All Headless Compression screws from Synthes are available both in stainless implant-grade steel and high-quality bio-compatible titanium alloy (TAN)

Functional Principle

Lag Screw Technique with Compression Sleeve

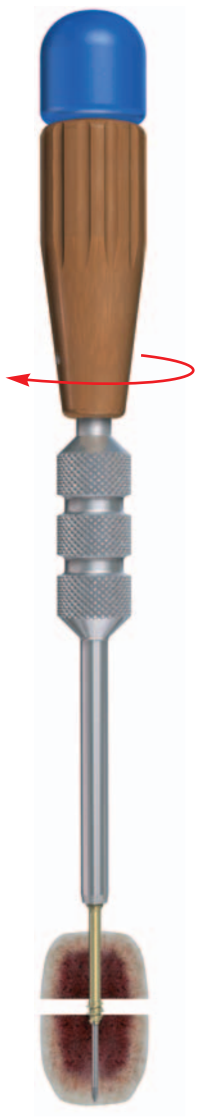

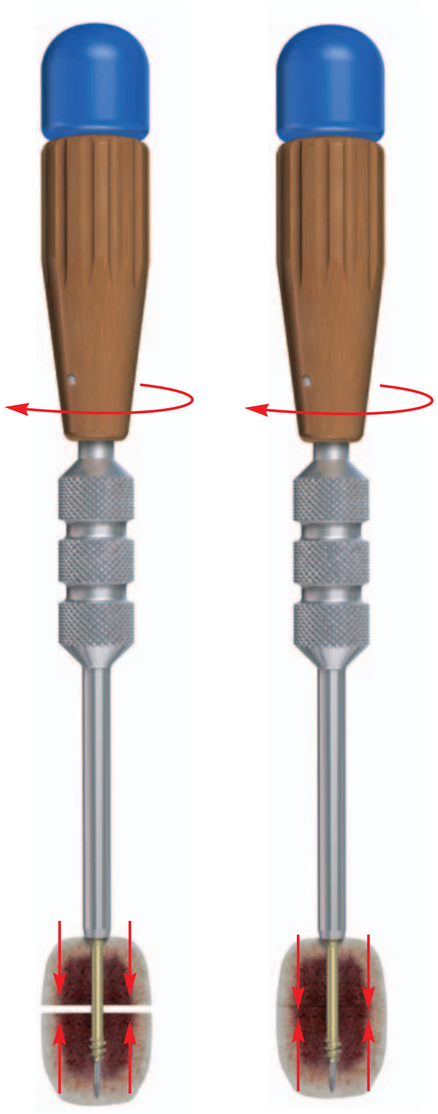

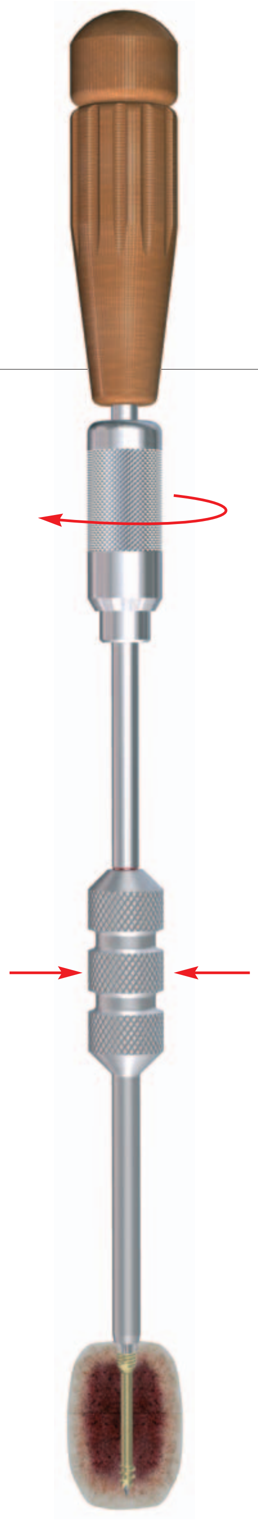

Step 1: Screw insertion
Insertion of the screw into the bone with the compression sleeve.

Step 2: Closure of gap and compression
Once the tip of the compression sleeve lies on the bone, the fracture gap is closed and compressed by further turning of the sleeve.

Step 3: Countersinking
Once the desired degree of compression is reached, the screw is countersunk into the bone with the screwdriver while the compression sleeve is held stationary. During countersinking no additional compression is generated.

Indications

– Fixation of intra-articular and extra-articular fractures and non-unions of small bones and small bone fragments
– Arthrodeses of small joints
– Bunionectomies and osteotomies

Examples include, but are not limited to scaphoid and other carpal bones, metacarpals, tarsals, metatarsals, patella, ulnar styloid, capitellum, radial head and radial styloid.

The following simplified surgical technique for a scaphoid fracture serves as example for the use of the HCS 2.4 or 3.0 in the hand.

1
Insert the guide wire

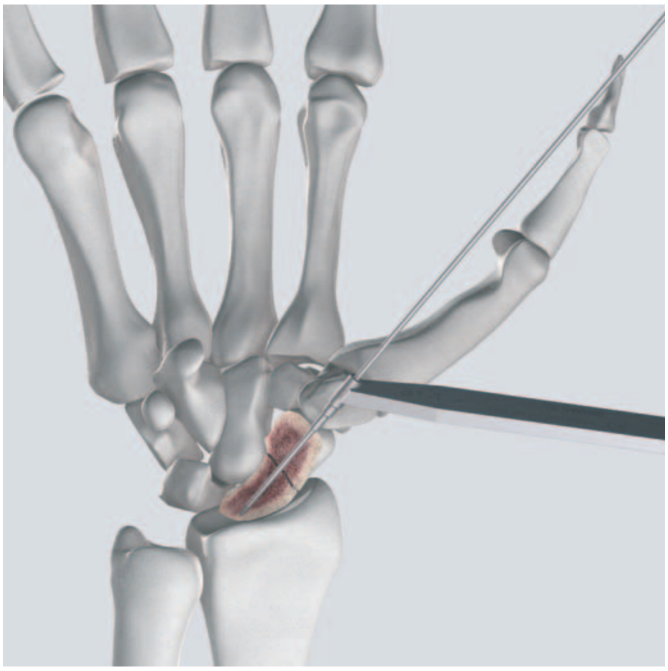

Instruments	
292.623	Guide Wire ∅ 1.1 mm with trocar tip, length 150 mm, Stainless Steel
or	
292.622	Guide Wire ∅ 1.1 mm with threaded tip, with trocar, length 150 mm, Stainless Steel
312.151	Double Drill Guide 2.0/1.1

While monitoring with the image intensifier, advance the guide wire through the drill guide into the bone until the thread tip is anchored in the far cortex.

Note: Do not forcefully insert the guide wire. This may cause it to bend.

2
Option: ream the trapezium

**Instruments**	
03.226.003	Trapezium Burr, cannulated, for HCS – Headless Compression Screw ∅ 2.4/3.0 mm
03.226.005	Protection Sleeve for HCS – Headless Compression Screw ∅ 2.4/3.0 mm, for Trapezium Burr
311.430	Handle with Quick Coupling, length 110 mm

To facilitate screw insertion, the flank of the trapezium can be removed with the trapezium burr.

Slide the trapezium burr with the protection sleeve over the guide wire and carefully ream the trapezium.

Ensure that the trapezium burr does not damage the scaphoid by using image intensification.

Note: Do not forcefully insert the trapezium burr since this may damage the guide wire.

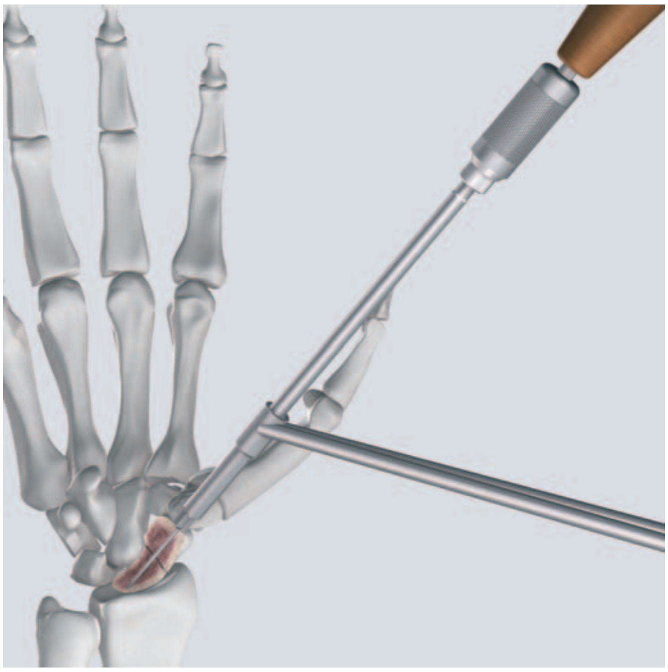

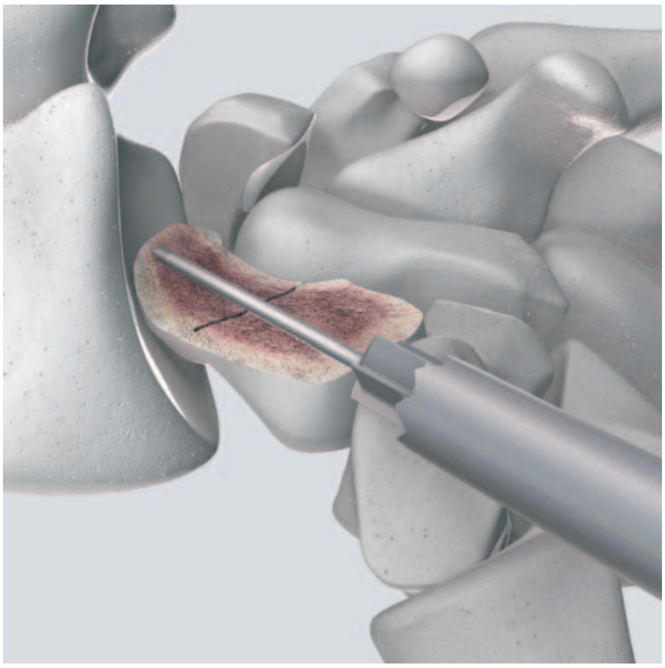

3
Determine screw and thread length

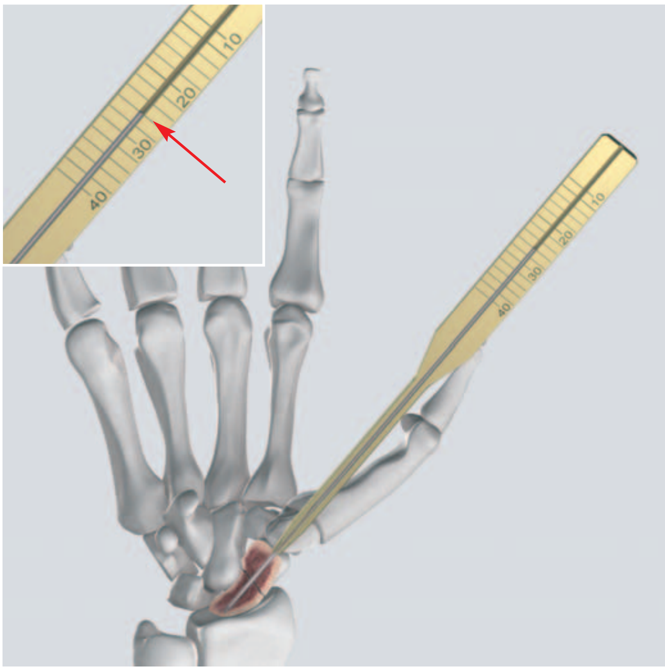

Instruments	
03.226.002	Direct Measuring Device for HCS – Headless Compression Screw ∅ 2.4/3.0 mm
292.623<br>or	Guide Wire ∅ 1.1 mm with trocar tip, length 150 mm, Stainless Steel
292.622	Guide Wire ∅ 1.1 mm with threaded tip, with trocar, length 150 mm, Stainless Steel

Slide the narrow end of the measuring device over the guide wire to the bone.

The measurement on the measuring device shows the depth of the guide wire in the bone in millimeters and directly the appropriate screw length.

If the screw is to be countersunk below the surface of the bone, subtract the appropriate screw length. If a large fracture gap needs to be closed or if the screw is inserted at an angle to the bone surface, substract more.

Note: Only use the guide wire in its original length to ensure correct measurement.

The position of the fracture line determines the thread length

Correctly selected thread length

The shaft thread lies completely within the proximal fragment during compression. Fragments can hence be compressed.

Incorrect thread length

The shaft thread lies over the fracture gap. Fragments cannot be compressed.

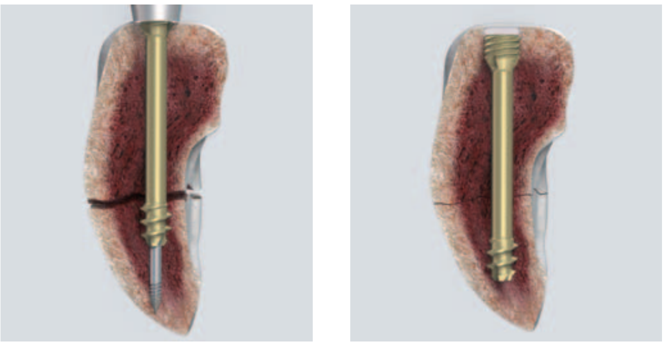

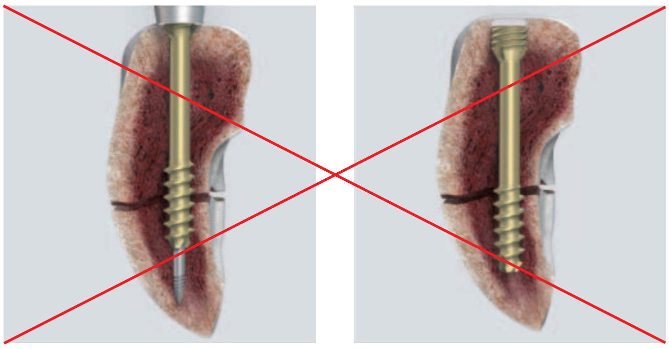

Note: If there is no good bone quality in the distal part of the bone, the distal screw thread can strip-out if too much compression is applied.

4
Predrilling

Instruments	
310.221	Drill Bit ∅ 2.0/1.15 mm, cannulated, length 150/48 mm, 3-flute, for Quick Coupling
312.151	Double Drill Guide 2.0/1.1

Predrilling makes it substantially easier to insert the screw in dense bone.

Slide the drill guide with the drill bit over the guide wire and pre-drill to the desired depth.

Remove the drill guide and verify the effective drilling depth with the image intensifier.

Note: Do not drill beyond the tip of the guide wire. Slowly pull the drill bit straight out while running the power tool in “forward mode” to ensure that the guide wire stays in place.

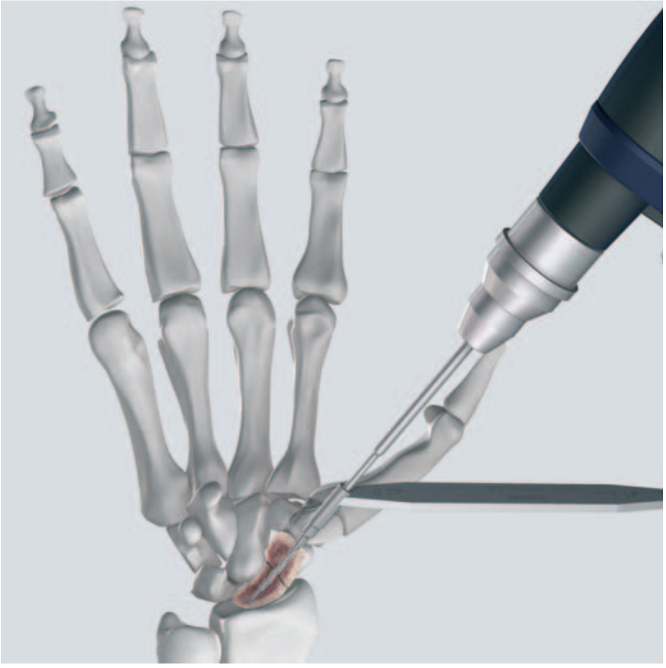

5
Pick up screw

Instruments	
**For HCS 2.4**	
03.226.016	Compression Sleeve for HCS – Headless Compression Screw ∅ 2.4 mm
**For HCS 3.0**	
03.226.000	Compression Sleeve for HCS – Headless Compression Screw ∅ 3.0 mm

Twist the compression sleeve over the head thread of the screw to remove the screw from the screw rack.

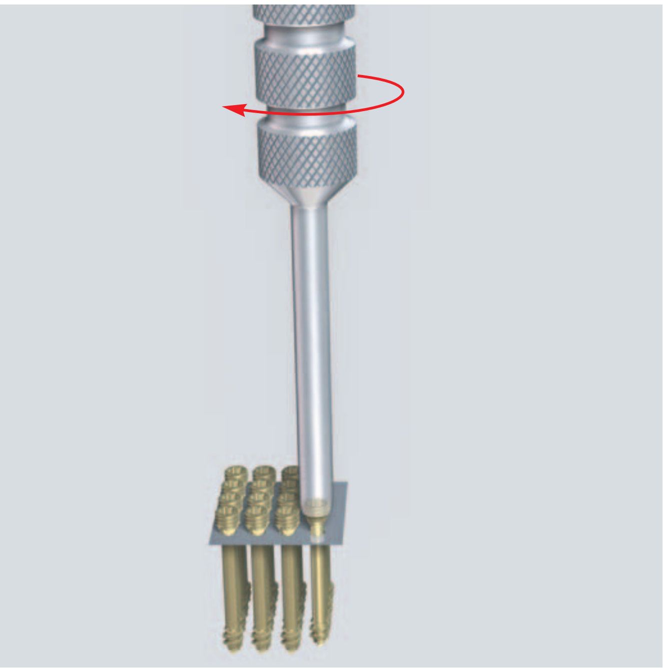

6
Insert screw and compress fragment

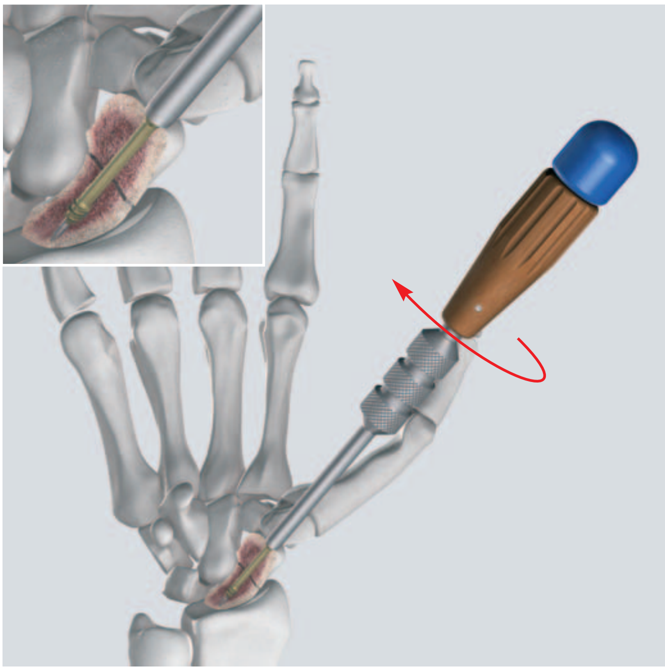

Instruments	
03.226.006	Handle for Compression Sleeve, for HCS – Headless Compression Screw ∅ 1.5–3.0 mm
**For HCS 2.4**	
03.226.016	Compression Sleeve for HCS – Headless Compression Screw ∅ 2.4 mm
**For HCS 3.0**	
03.226.000	Compression Sleeve for HCS – Headless Compression Screw ∅ 3.0 mm

Slide the handle into the selected compression sleeve. Insert the screw into the bone until the fracture gap or the osteotomy is closed and compressed.

Notes

- Verify the correct position of the shaft thread in the proximal fragment using the image intensifier. If the thread lies over the fracture gap or the osteotomy, the gap cannot be compressed.
- Carefully tighten the screw with the compression sleeve. Forceful tightening could cause stripping of the shaft thread.
- If the thread strips, some or all of the compression will be lost. If the screw is then countersunk correctly, the thread will regain purchase, thereby reducing the danger of postoperative screw loosening.
- If loss of compression makes screw extraction necessary, follow the instructions on screw extraction on page 22.

7
Countersink screw

**Instruments**	
03.226.004	Screwdriver Shaft, cannulated, Stardrive, T8, with coloured marking, for HCS – Headless Compression Screw ∅ 2.4 / 3.0 mm
311.430	Handle with Quick Coupling, length 110 mm
**For HCS 2.4**	
03.226.016	Compression Sleeve for HCS – Headless Compression Screw ∅ 2.4 mm
**For HCS 3.0**	
03.226.000	Compression Sleeve for HCS – Headless Compression Screw ∅ 3.0 mm

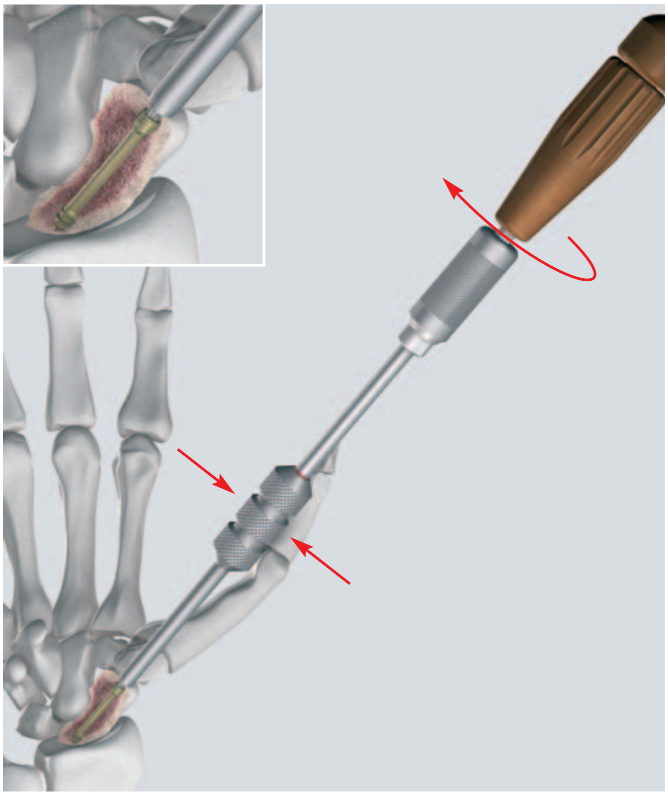

Remove the compression sleeve handle and slide the cannulated screwdriver through the compression sleeve.

Countersink the screw by turning the screwdriver shaft while simultaneously holding the compression sleeve stationary.

Verify the screw position with the image intensifier. Ensure that the screw tip does not penetrate the proximal cortex. Remove and dispose of the guide wire.

Color markings

The color markings on the screwdriver shaft show the position of the screwdriver tip and the head thread of the screw.

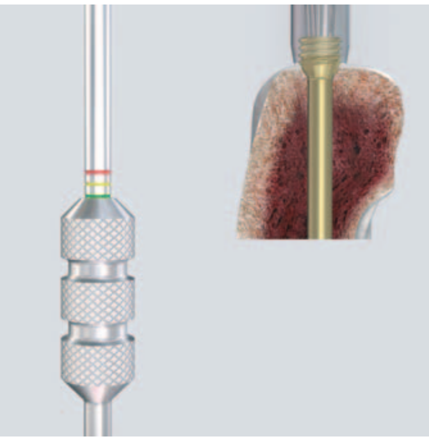

Green mark at the top end of the compression sleeve
The screwdriver tip is seated correctly in the Stardrive recess of the screw.

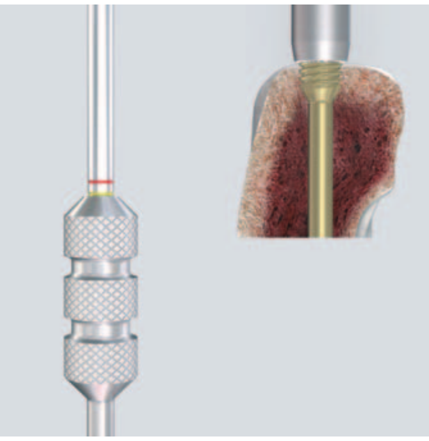

Yellow mark at the top end of the compression sleeve
The top end of the head thread is even with the bone surface.

Note: If the screw is inserted at an angle, it must be countersunk further than the yellow mark so that it does not project from the surface.

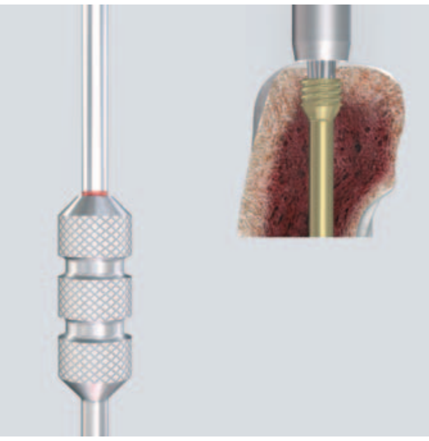

Red mark at the top end of the compression sleeve
The top end of the head thread is approximately 2 mm below the bone surface.

Foot – Chevron Osteotomy for Hallux Valgus

The following simplified surgical technique for a chevron osteotomy serves as example for the use of the HCS 2.4 or 3.0 in the foot.

1

Remove bunion and perform V-shaped osteotomy

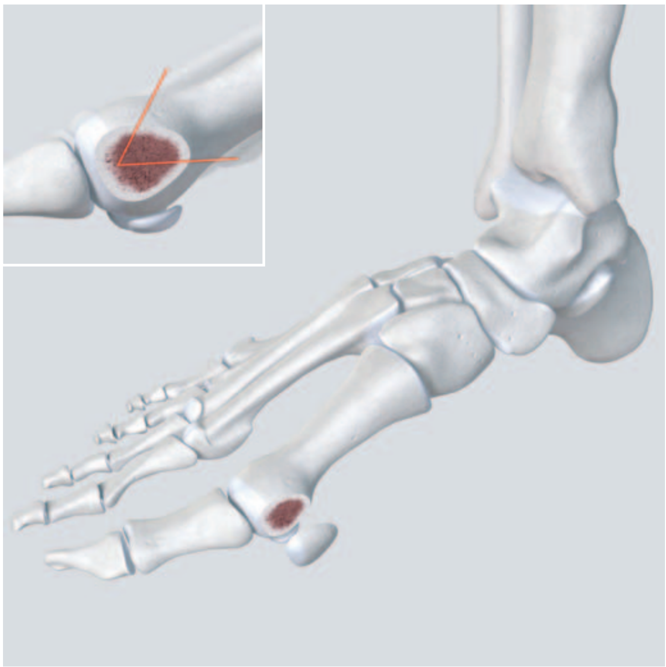

Remove the bunion on the medial side of the first metatarsal with a saw blade under image intensification.

Perform a V-shaped osteotomy (inner angle approx. 55°), with the peak approximately 2 mm distal from the center of the head of the first metatarsal.

2
Move the distal fragment laterally

Move the distal fragment in a lateral direction to correct the alignment.

3
Insert the guide wire

**Instruments**	
292.623	Guide Wire ∅ 1.1 mm with trocar tip, length 150 mm, Stainless Steel
or	
292.622	Guide Wire ∅ 1.1 mm with threaded tip, with trocar, length 150 mm, Stainless Steel
312.151	Double Drill Guide 2.0/1.1

While monitoring with the image intensifier, advance the guide wire through the double drill guide from proximal dorsal to distal plantar through the osteotomy into the bone until the thread tip is anchored in the far cortex.

Note: Do not forcefully insert the guide wire. This may cause it to bend.

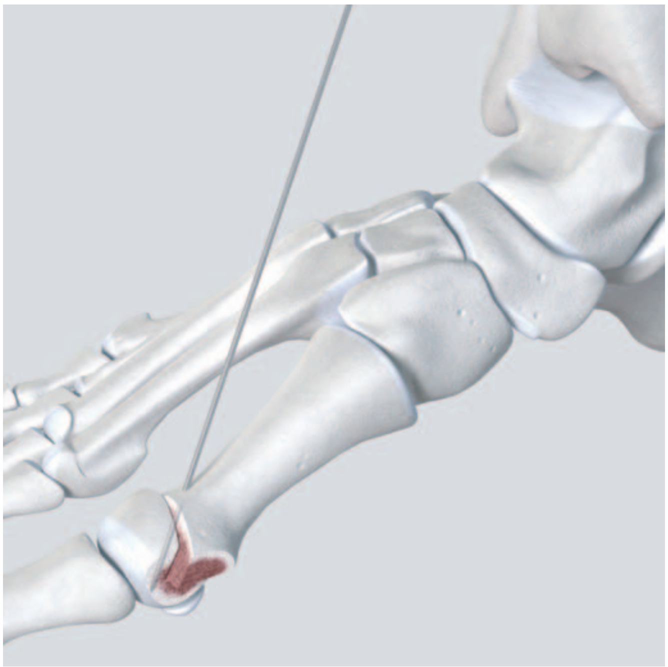

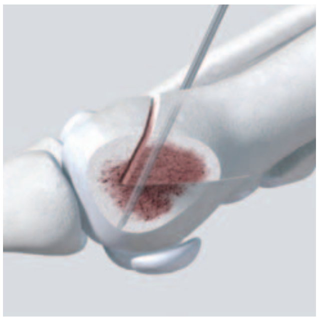

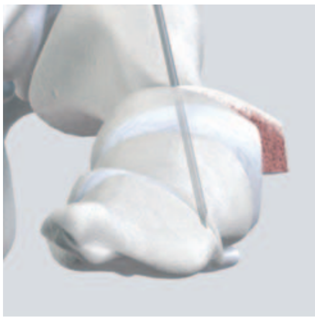

4
Determine screw and thread length

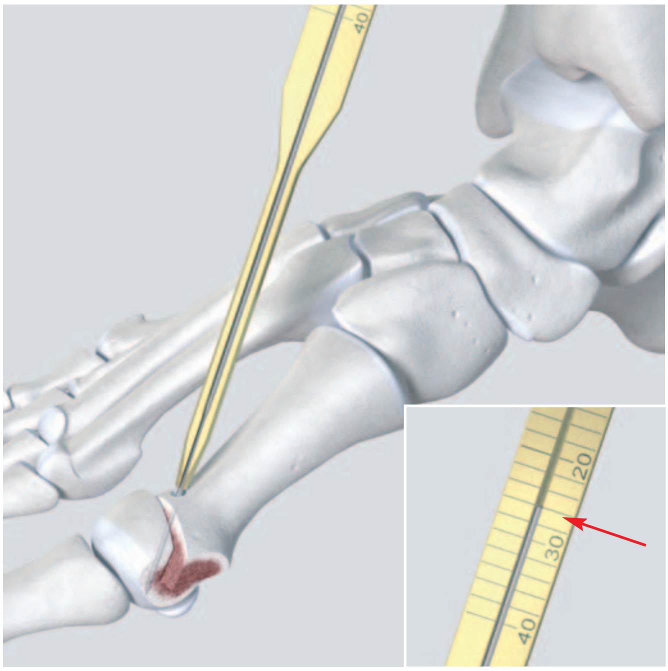

Instrument	
03.226.002	Direct Measuring Device for HCS – Headless Compression Screw ∅ 2.4/3.0 mm

Slide the narrow end of the measuring device over the guide wire to the bone.

The measurement on the measuring device shows the depth of the guide wire in the bone in millimeters.

If the screw is to be countersunk below the surface of the bone, subtract the appropriate screw length. If a large fracture gap needs to be closed or if the screw is inserted at an angle to the bone surface, substract more.

Note: The position of the osteotomy line determines the thread length (see page 8).

5
Predrilling

Instruments	
310.221	Drill Bit ∅ 2.0/1.15 mm, cannulated, length 150/48 mm, 3-flute, for Quick Coupling
312.151	Double Drill Guide 2.0/1.1

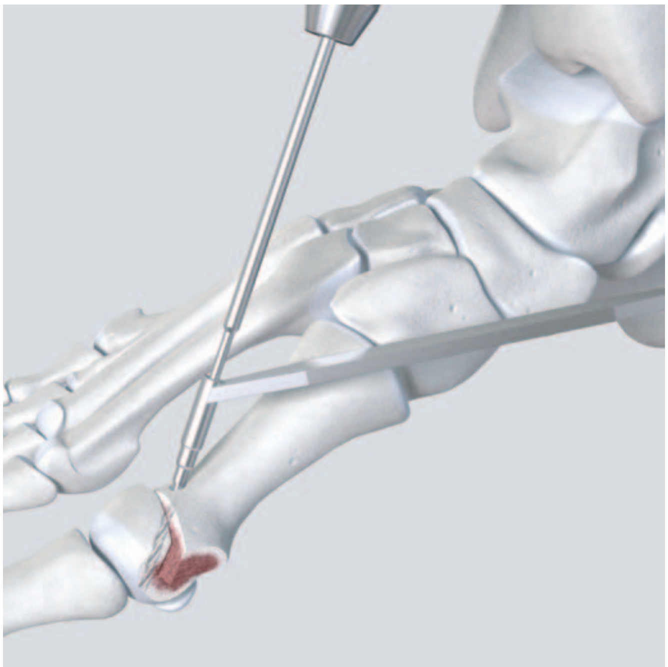

Predrilling makes it substantially easier to insert the screw in dense bone.

Slide the double drill guide with the drill bit over the guide wire and predrill to the desired depth.

Verify the effective drilling depth with the image intensifier.

Note: Do not drill beyond the tip of the guide wire. Slowly pull the drill bit straight out while running the power tool in "forward mode" to ensure that the guide wire stays in place.

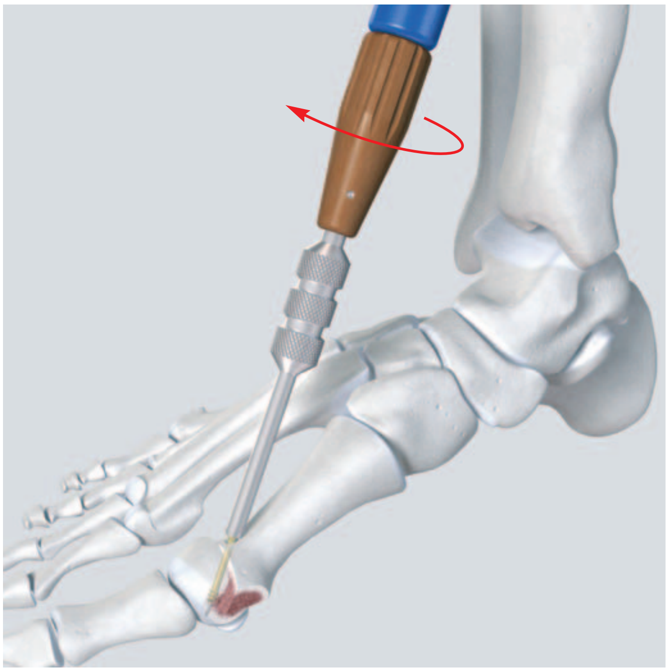

6
Insert screw and compress osteotomy

**Instruments**	
03.226.000	Compression Sleeve for HCS – Headless Compression Screw ∅ 3.0 mm
03.226.006	Handle for Compression Sleeve, for HCS – Headless Compression Screw ∅ 1.5-3.0 mm
**For HCS 2.4**	
03.226.016	Compression Sleeve for HCS – Headless Compression Screw ∅ 2.4 mm
**For HCS 3.0**	
03.226.000	Compression Sleeve for HCS – Headless Compression Screw ∅ 3.0 mm

Twist the selected compression sleeve over the head thread of the screw to remove the screw from the screw rack.

Slide the handle into the compression sleeve. Insert the screw into the bone until the osteotomy is closed and compressed.

Notes
- Verify the correct position of the shaft thread in the distal fragment using the image intensifier. If the thread lies over the osteotomy, the gap cannot be compressed.
- Carefully tighten the screw with the compression sleeve. Forceful tightening could cause stripping of the shaft thread.
- If the thread strips, some or all of the compression will be lost. If the screw is then countersunk correctly, the thread will regain purchase, thereby reducing the danger of postoperative screw loosening.
- If loss of compression makes screw extraction necessary, follow the instructions on screw extraction on page 22.

7
Countersink screw

Instruments	
03.226.000	Compression Sleeve for HCS – Headless Compression Screw ∅ 3.0 mm
03.226.004	Screwdriver Shaft, cannulated, Stardrive, T8, with coloured marking, for HCS – Headless Compression Screw ∅ 2.4/3.0 mm
311.430	Handle with Quick Coupling, length 110 mm
**For HCS 2.4**	
03.226.016	Compression Sleeve for HCS – Headless Compression Screw ∅ 2.4 mm
**For HCS 3.0**	
03.226.000	Compression Sleeve for HCS – Headless Compression Screw ∅ 3.0 mm

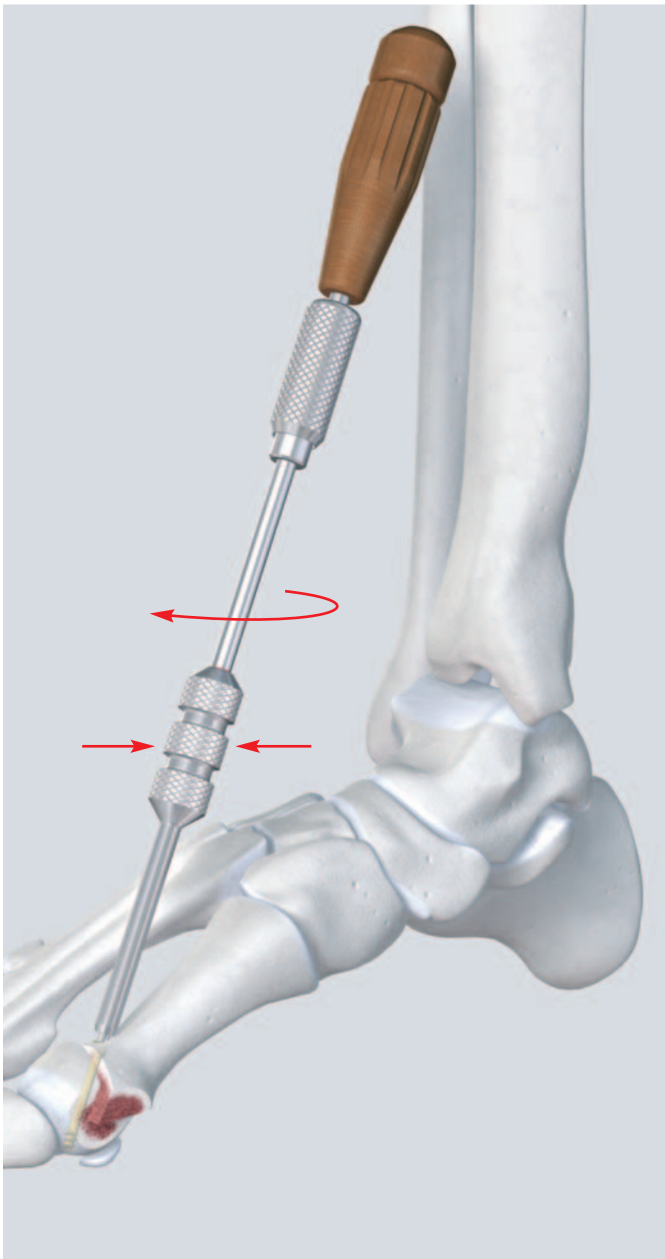

Remove the compression sleeve handle and slide the cannulated screwdriver through the compression sleeve.

Countersink the screw by turning the screwdriver shaft while simultaneously holding the compression sleeve stationary.

Verify the screw position with the image intensifier. Ensure that the screw tip does not penetrate the distal cortex. Remove and dispose of the guide wire.

Note: The color markings on the screwdriver shaft show the position of the screwdriver tip and head thread of the screw (see page 13).

8
Remove protruding bone

Remove the protruding bone of the proximal fragment.

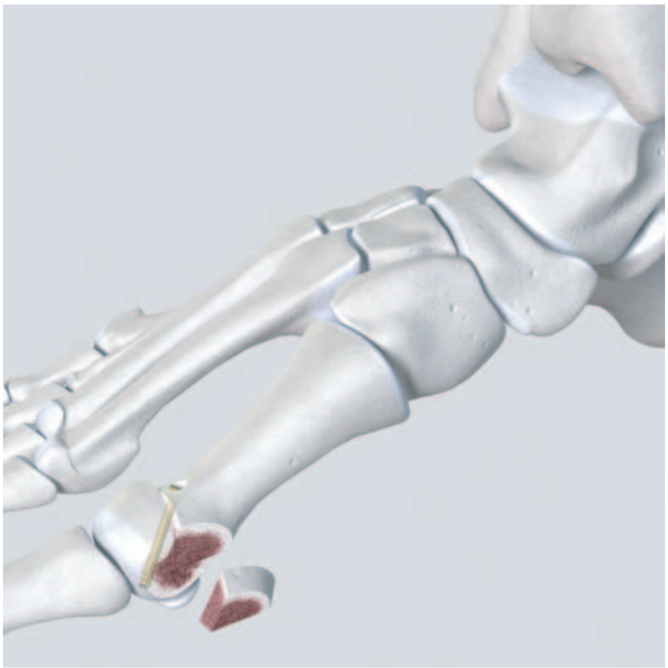

Using the Optional Drill Guide with Stop

The drill guide with stop allows control of the drilling depth and can be used for drilling instead of the double drill guide 2.0/1.1 (312.151).

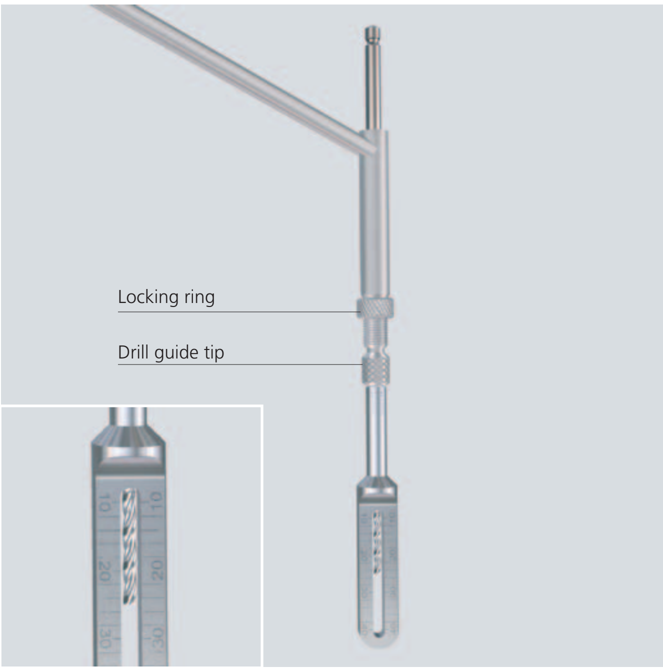

**Instruments**	
310.221	Drill Bit ∅ 2.0/1.15 mm, cannulated, length 150/48 mm, 3-flute, for Quick Coupling
03.226.007	Drill Guide with Stop for Drill Bits ∅ 2.0/1.15 mm No. 310.221
03.226.008	Direct Measuring Device for Drill Guide with Stop No. 03.226.007

To set the drilling depth, insert the drill bit in the drill guide with stop, and slide the measuring device over the drill bit until the retaining device is engaged.

Release the locking ring, and set the drilling depth by rotating the tip of the drill guide. The measurement on the measuring device indicates the set drilling depth in millimeters.

Tighten the locking ring to fix the drilling depth.

Screw Extraction

**Instruments**	
314.467	Screwdriver Shaft, Stardrive, T8, self-holding
or	
03.226.004	Screwdriver Shaft, cannulated, Stardrive, T8, with coloured marking, for HCS – Headless Compression Screw ∅ 2.4/3.0 mm
311.430	Handle with Quick Coupling, length 110 mm
**For HCS 2.4**	
03.226.016	Compression Sleeve for HCS – Headless Compression Screw ∅ 2.4 mm
**For HCS 3.0**	
03.226.000	Compression Sleeve for HCS – Headless Compression Screw ∅ 3.0 mm

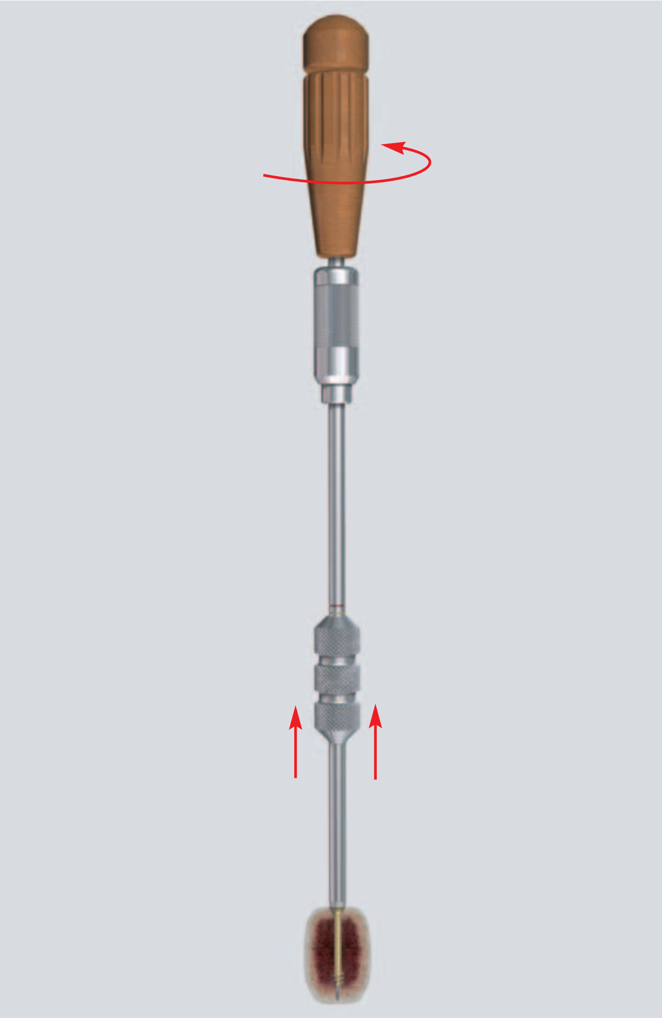

For the extraction of the HCS use a Stardrive screwdriver or Screwdriver Shaft in combination with the handle.

If the screw strips, use the following procedure:

Twist the compression sleeve over the head thread and insert the screwdriver through the compression sleeve into the Stardrive recess of the screw.

Remove the screw by simultaneously pulling on the compression sleeve and turning both the screwdriver and the compression sleeve in counterclockwise direction.

Note: If necessary, expose the recess and part of the head thread with a hollow reamer (e.g. 309.035) or preferred method.

HCS 2.4 mm – Headless Compression Screw, short thread

Art. No.	Screw length (mm) L	Shaft thread length (mm) S
0X.226.209	9	4
0X.226.210	10	4
0X.226.211	11	4
0X.226.212	12	4
0X.226.213	13	4
0X.226.214	14	4
0X.226.215	15	4
0X.226.216	16	4
0X.226.217	17	4
0X.226.218	18	4
0X.226.219	19	4
0X.226.220	20	4
0X.226.221	21	4
0X.226.222	22	4
0X.226.223	23	4
0X.226.224	24	5
0X.226.225	25	5
0X.226.226	26	5
0X.226.227	27	6
0X.226.228	28	6
0X.226.229	29	6
0X.226.230	30	7
0X.226.232	32	7
0X.226.234	34	8
0X.226.236	36	9
0X.226.238	38	9
0X.226.240	40	10

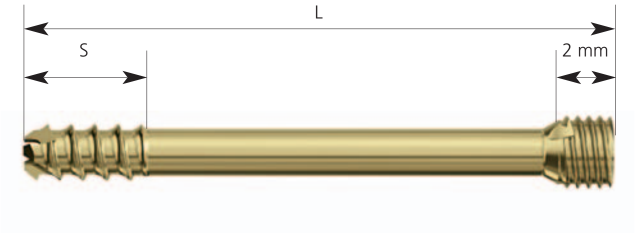

X = 2: Stainless Steel
X = 4: Titanium Alloy (TAN)

All implants are also available sterile packed. Add suffix "S" to article number.

HCS 2.4 mm – Headless Compression Screw, long thread

Art. No.	Screw length (mm) L	Shaft thread length (mm) S
0X.226.316	16	5
0X.226.317	17	6
0X.226.318	18	6
0X.226.319	19	7
0X.226.320	20	7
0X.226.321	21	8
0X.226.322	22	8
0X.226.323	23	8
0X.226.324	24	8
0X.226.325	25	8
0X.226.326	26	10
0X.226.327	27	10
0X.226.328	28	10
0X.226.329	29	10
0X.226.330	30	12
0X.226.332	32	12
0X.226.334	34	14
0X.226.336	36	14
0X.226.338	38	16
0X.226.340	40	16

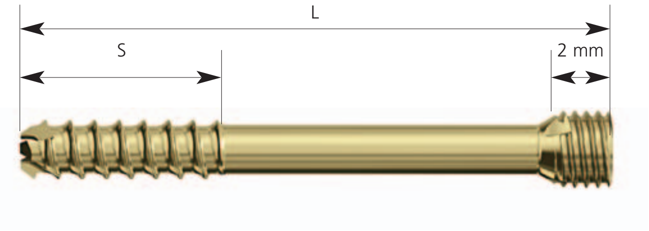

X = 2: Stainless Steel
X = 4: Titanium Alloy (TAN)

All implants are also available sterile packed. Add suffix "S" to article number.

HCS 3.0 mm – Headless Compression Screw, short thread

Art. No.	Screw length (mm) L	Shaft thread length (mm) S
0X.226.010	10	4
0X.226.011	11	4
0X.226.012	12	4
0X.226.013	13	4
0X.226.014	14	4
0X.226.015	15	4
0X.226.016	16	4
0X.226.017	17	4
0X.226.018	18	4
0X.226.019	19	4
0X.226.020	20	4
0X.226.021	21	4
0X.226.022	22	4
0X.226.023	23	4
0X.226.024	24	5
0X.226.025	25	5
0X.226.026	26	5
0X.226.027	27	6
0X.226.028	28	6
0X.226.029	29	6
0X.226.030	30	7
0X.226.032	32	7
0X.226.034	34	8
0X.226.036	36	9
0X.226.038	38	9
0X.226.040	40	10

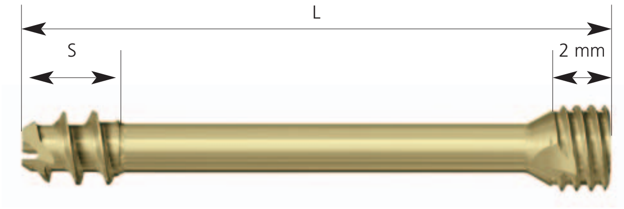

X = 2: Stainless Steel
X = 4: Titanium Alloy (TAN)

All implants are also available sterile packed. Add suffix "S" to article number.

HCS 3.0 mm – Headless Compression Screw, long thread

Art. No.	Screw length (mm) L	Shaft thread length (mm) S
0X.226.116	16	5
0X.226.117	17	6
0X.226.118	18	6
0X.226.119	19	7
0X.226.120	20	7
0X.226.121	21	8
0X.226.122	22	8
0X.226.123	23	8
0X.226.124	24	8
0X.226.125	25	8
0X.226.126	26	10
0X.226.127	27	10
0X.226.128	28	10
0X.226.129	29	10
0X.226.130	30	12
0X.226.132	32	12
0X.226.134	34	14
0X.226.136	36	14
0X.226.138	38	16
0X.226.140	40	16

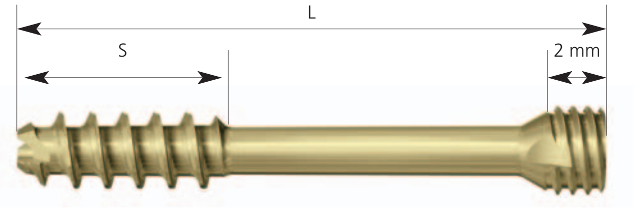

X = 2: Stainless Steel
X = 4: Titanium Alloy (TAN)

All implants are also available sterile packed. Add suffix "S" to article number.

Instruments HCS 2.4 and 3.0

292.623	Guide Wire ∅ 1.1 mm with trocar tip<br>length 150 mm, Stainless Steel	
312.151	Double Drill Guide 2.0/1.1<br>For protecting soft tissue during insertion of guide wires and predrilling	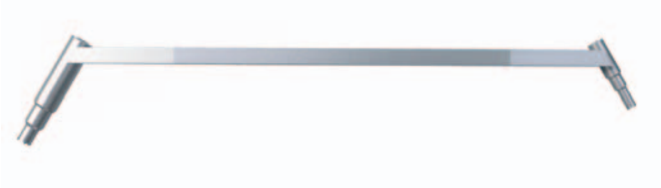
03.226.002	Direct Measuring Device for HCS – Headless Compression Screw<br>∅ 2.4/3.0 mm	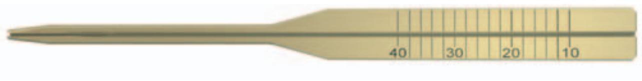
310.221	Drill Bit ∅ 2.0/1.15 mm, cannulated,<br>length 150/48 mm, 3-flute,<br>for Quick Coupling<br>For predrilling	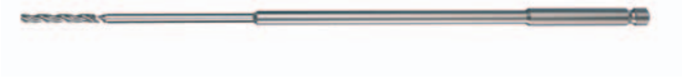
03.226.006	Handle for Compression Sleeve, for HCS – Headless Compression Screw<br>∅ 1.5–3.0 mm	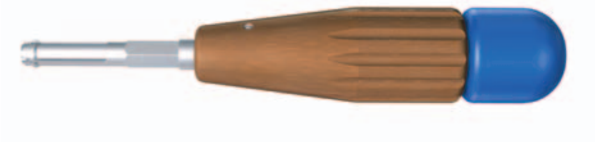
311.430	Handle with Quick Coupling,<br>length 110 mm<br>For Stardrive T8 Screwdriver Shafts<br>(03.226.004 and 314.467)	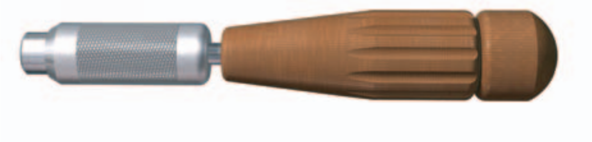
03.226.004	Screwdriver Shaft, cannulated, Stardrive,<br>T8, with coloured marking,<br>for HCS – Headless Compression Screw<br>∅ 2.4/3.0 mm<br>For countersinking the screw; with color markings to control countersink depth	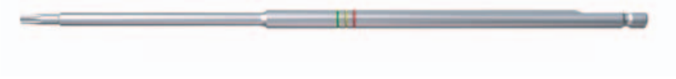

314.467	Screwdriver Shaft, Stardrive T8, self-holding<br>For screw extraction; with self-retaining tip	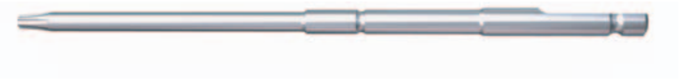
319.970	Screw Forceps, self-holding, length 85 mm	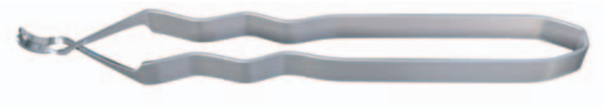
319.291	Cleaning Brush ∅ 1.25 mm, for Cannulated Instruments<br>For postoperative cleaning of cannulated instruments	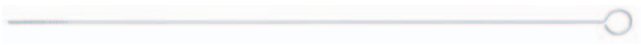
319.292	Cleaning Stylet ∅ 1.1 mm, for Cannulated Instruments<br>For cleaning cannulated instruments during surgery	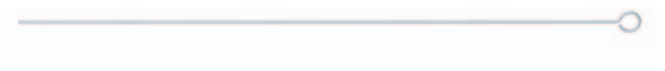
For HCS 2.4<br>03.226.016	Compression Sleeve for HCS – Headless Compression Screw ∅ 2.4 mm<br>For closing the fracture gap and compressing the bone fragments	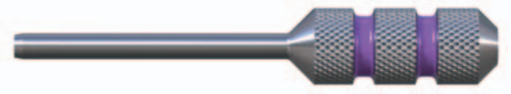
For HCS 3.0<br>03.226.000	Compression Sleeve for HCS – Headless Compression Screw ∅ 3.0 mm<br>For closing the fracture gap and compressing the bone fragments	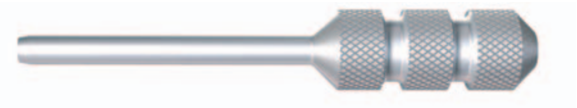

Optional Instruments for HCS 2.4 and 3.0

292.622	Guide Wire ∅ 1.1 mm with threaded tip with trocar, length 150 mm, Stainless Steel	
03.226.003	Trapezium Burr, cannulated<br>For freeing the palmar approach to the distal pole of the scaphoid	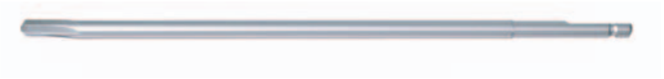
03.226.005	Protection Sleeve for HCS – Headless Compression Screw ∅ 2.4/3.0 mm for Trapezium Burr<br>For protecting soft tissue during use of the trapezium burr	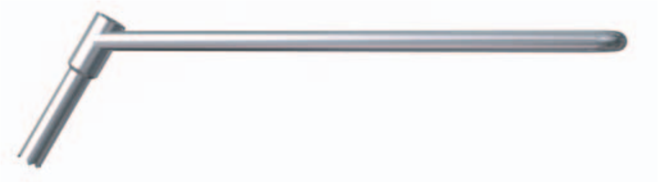
03.226.007	Drill Guide with Stop for Drill Bits ∅ 2.0/1.15 mm No. 310.221<br>For controlled drilling	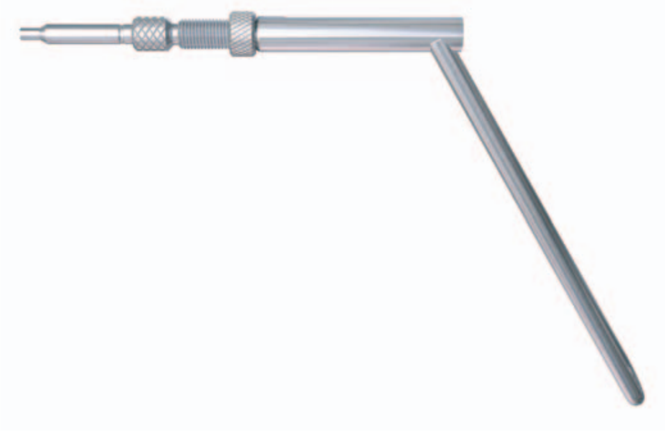
03.226.008	Direct Measuring Device for Drill Guide with Stop No. 03.227.007<br>For determining the drilling depth	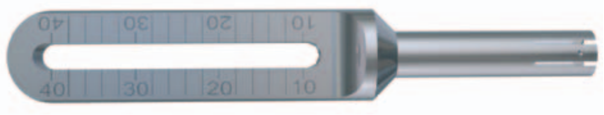
398.408	Periosteal Elevator, slightly curved blade, round tip, width 5 mm<br>For manipulating small bones and bone fragments	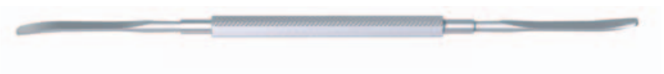
398.409	Sharp Reduction Hook, graded<br>For levering up carpal bones	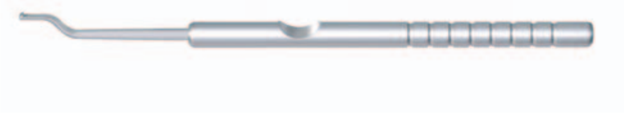

Setlists HCS 2.4 and 3.0

HCS 2.4

01.226.012	Instrument and Implant Set for HCS – Headless Compression Screw ∅ 2.4 mm (Stainless Steel) for Vario Case
01.226.014	Instrument and Implant Set for HCS – Headless Compression Screw ∅ 2.4 mm (Titanium Alloy) for Vario Case

To adapt for use with the HCS 3.0, the following articles must additionally be ordered:

03.226.000	Compression Sleeve for HCS – Headless Compression Screw ∅ 3.0 mm
68.111.443	Insert for Screw Rack Module, for HCS – Headless Compression Screw ∅ 3.0 mm

HCS 3.0

01.226.002	Set for Instruments and Implants for HCS – Headless Compression Screw ∅ 3.0 mm (Stainless Steel), for Vario Case
01.226.004	Set for Instruments and Implants for HCS – Headless Compression Screw ∅ 3.0 mm (Titanium Alloy), for Vario Case

To adapt for use with the HCS 2.4, the following articles must additionally be ordered:

03.226.016	Compression Sleeve for HCS – Headless Compression Screw ∅ 2.4 mm
68.111.446	Insert for Screw Rack Module, for HCS – Headless Compression Screw ∅ 2.4 mm

Synthes GmbH
Eimattstrasse 3
CH-4436 Oberdorf
www.synthes.com

All technique guides are available as PDF files at
www.synthes.com/lit

0123

036.000.323 SE_152904 AC 30100266
© 07/2010 Synthes, Inc. or its affiliates All rights reserved Synthes, Stardrive and Vario Case are trademarks of Synthes, Inc. or its affiliates
