# Supplementary material for: Impact of Different Screw Designs on Durability of Fracture Fixation: In Vitro Study with Cyclic Loading of Scaphoid Bones
Source: PLoS One. 2016 Jan 7;11(1):e0145949. doi: 10.1371/journal.pone.0145949 (PMC4704798; doi:10.1371/journal.pone.0145949)
Supplement: S4 Text — (PDF) [file pone.0145949.s007.pdf]

Handchirurgie

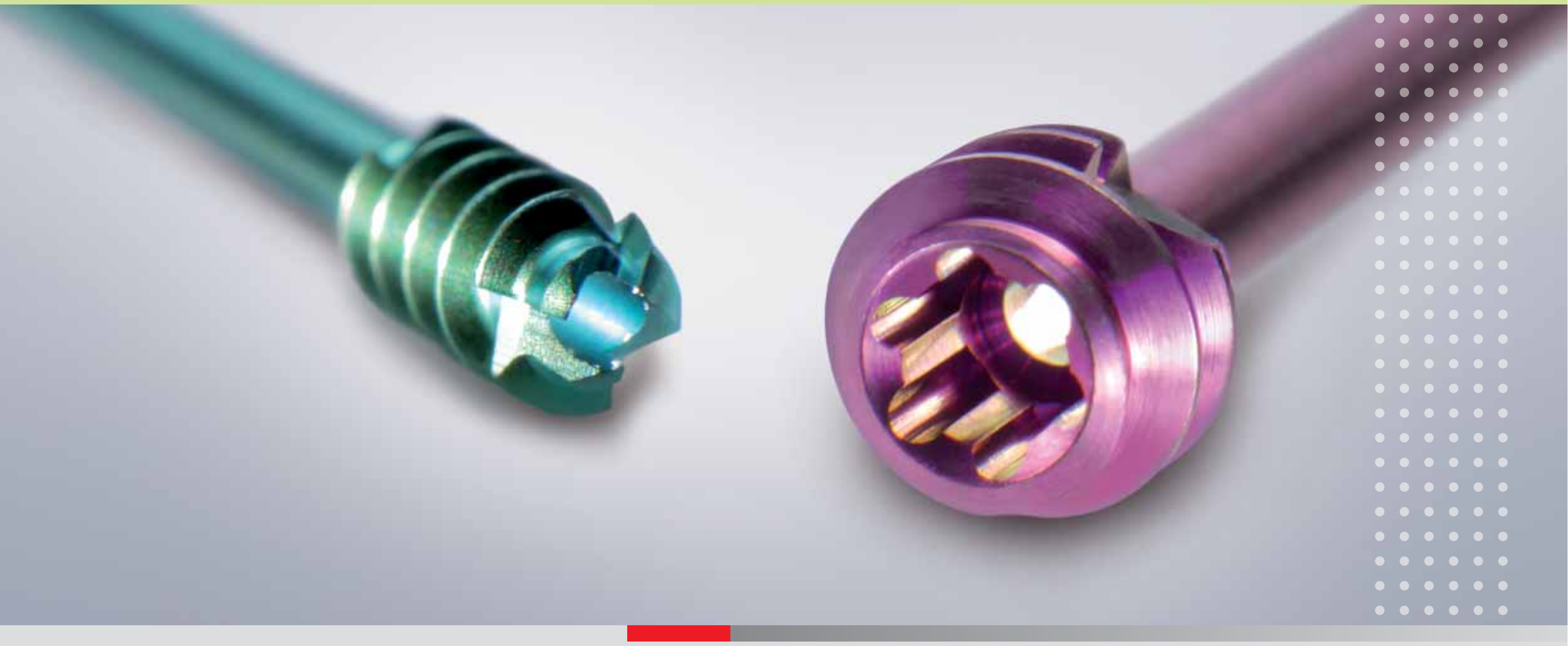

HBS2

Bewährtes im Detail verbessert!

reddot design award
winner 2011

KLS martin
GROUP

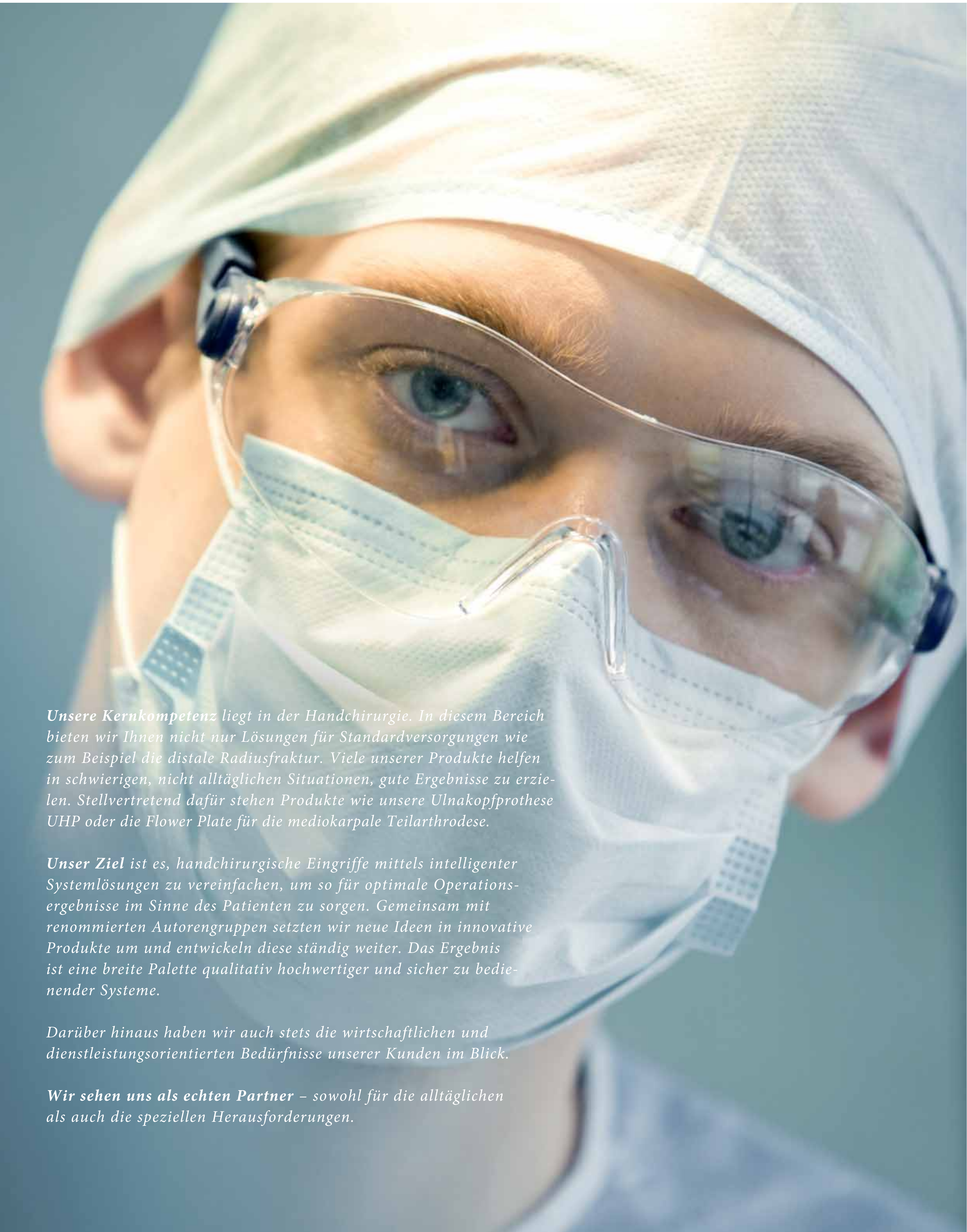

Unsere Kernkompetenz *liegt in der Handchirurgie. In diesem Bereich bieten wir Ihnen nicht nur Lösungen für Standardversorgungen wie zum Beispiel die distale Radiusfraktur. Viele unserer Produkte helfen in schwierigen, nicht alltäglichen Situationen, gute Ergebnisse zu erzielen. Stellvertretend dafür stehen Produkte wie unsere Ulnakopfprothese UHP oder die Flower Plate für die mediokarpale Teilarthrodese.*

Unser Ziel *ist es, handchirurgische Eingriffe mittels intelligenter Systemlösungen zu vereinfachen, um so für optimale Operationsergebnisse im Sinne des Patienten zu sorgen. Gemeinsam mit renommierten Autorengruppen setzten wir neue Ideen in innovative Produkte um und entwickeln diese ständig weiter. Das Ergebnis ist eine breite Palette qualitativ hochwertiger und sicher zu bedienender Systeme.*

Darüber hinaus haben wir auch stets die wirtschaftlichen und dienstleistungsorientierten Bedürfnisse unserer Kunden im Blick.

Wir sehen uns als echten Partner *– sowohl für die alltäglichen als auch die speziellen Herausforderungen.*

Inhaltsverzeichnis

HBS2 – Produktmerkmale 6-9

Indikationen und Operationstechniken 10-11

- Skaphoidfraktur im mittleren Drittel
  Minimalinvasive palmare OP-Technik 12-17
- Skaphoidpseudarthrose
  Offene palmare OP-Technik 18-25
- Skaphoidfraktur / Skaphoidpseudarthrose
  unter Verwendung des Schließaufsatzes
  Offene palmare OP-Technik 26-29

Produktsortiment

- HBS2-Implantate 30-31
- HBS2-Instrumente 32-35
- HBS2-Lagerung 36-37

Das KLS-Martin-Leihsystemwesen „marLOAN“ 38-39

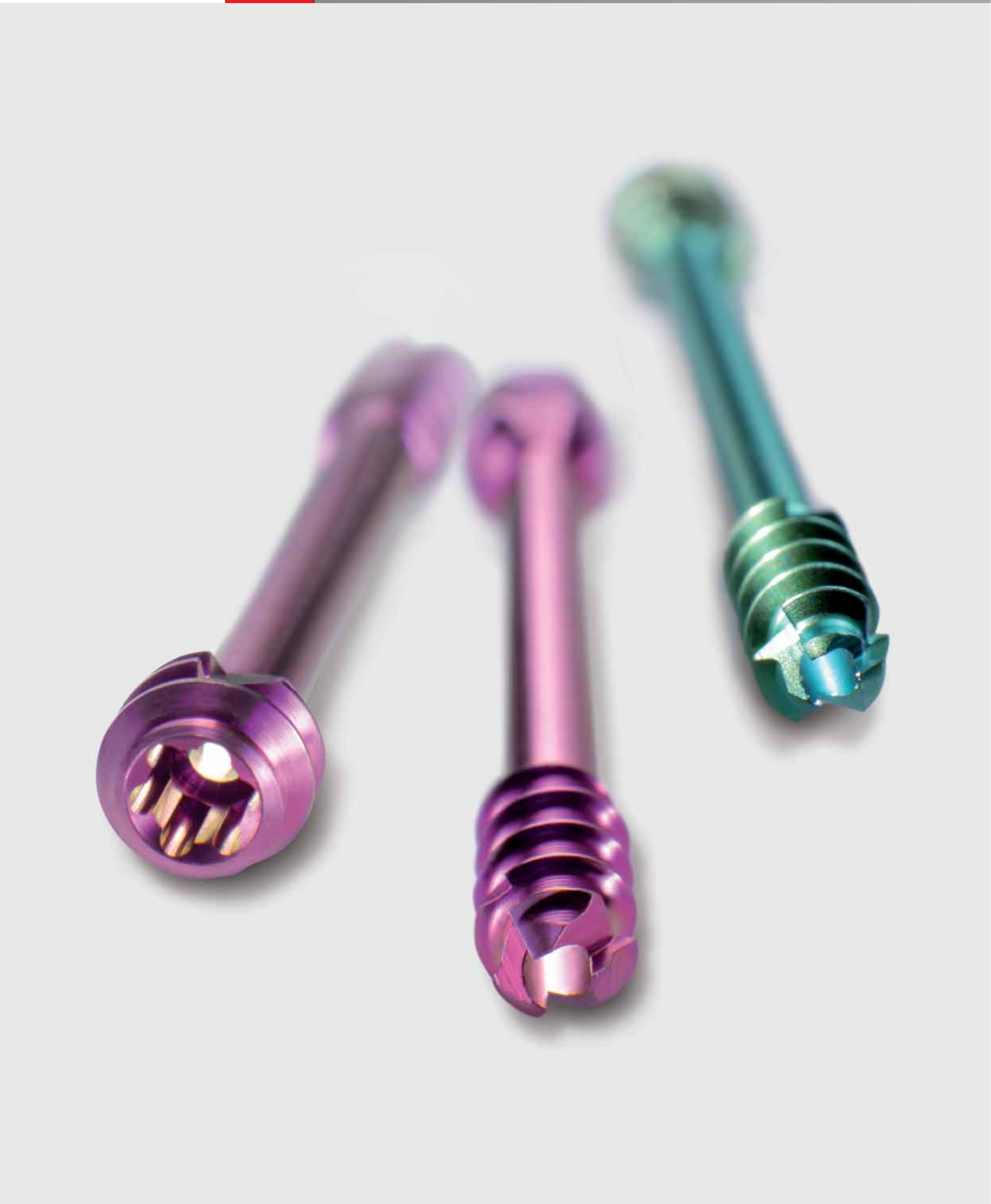

HBS2

Bewährtes im Detail verbessert!

Nach mehr als 100.000 erfolgreichen HBS-Implantationen weltweit war es an der Zeit, die gesammelten Erfahrungen in die neue Generation der Headless Bone Screw einfließen zu lassen.

Eine große Herausforderung bei der Gestaltung der HBS2-Implantate war es einerseits, die bewährte Implantatgeometrie beizubehalten, andererseits jedoch besser auf die individuelle Situation bei der Frakturversorgung eingehen zu können.

Ein weiteres Ziel war, unter Nutzung der nun verfügbaren Technologien die Handhabung von HBS2 für alle Beteiligten zu vereinfachen – den Operateur, das assistierende OP-Personal sowie die Mitarbeiter in der Zentralsterilisation. Durch die Reduktion der OP-Schritte und die verbesserte Handhabung des HBS2-Systems entstehen unmittelbar Vorteile für den Patienten, unberücksichtigt der wirtschaftlichen Aspekte.

Merkmal, Funktion und Nutzen

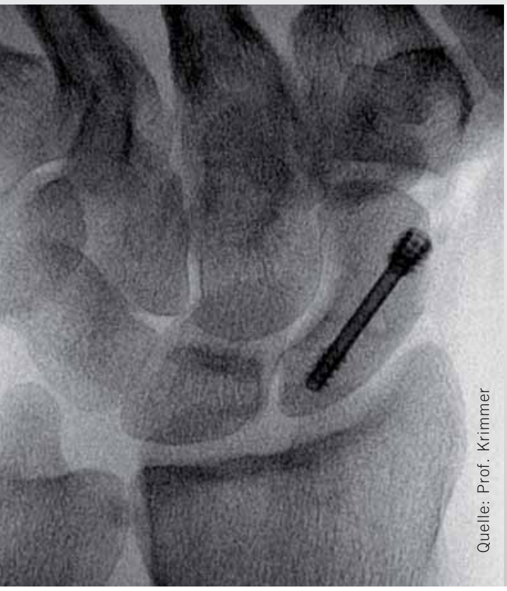

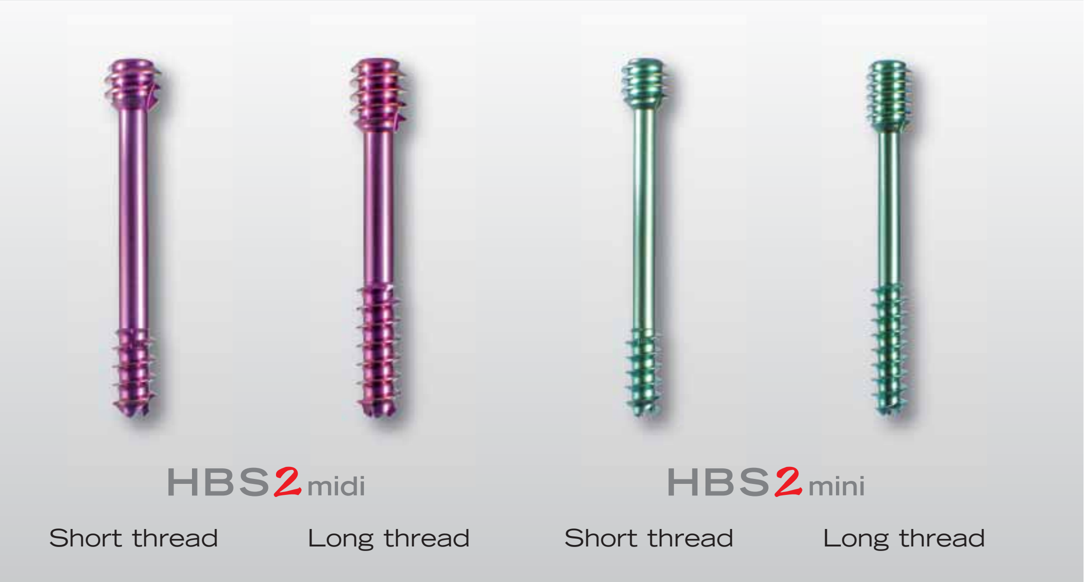

Die bewährte Grundgeometrie der HBS-Schrauben wurde beibehalten. HBS2-Schrauben sind in der Dimension midi und mini erhältlich. Dabei können nun Schrauben mit verschiedenen Gewindelängen gewählt werden:

	Gesamtlänge	Gewindelänge proximal	Gewindelänge distal
**HBS2 midi short thread**	10-30 mm (1-mm-Schritte)	3,5 mm	4-6 mm
**HBS2 midi long thread**	20-40 mm (2-mm-Schritte)	5,0 mm	8-13 mm
**HBS2 mini short thread**	10-30 mm (1-mm-Schritte)	3,5 mm	4-6 mm
**HBS2 mini long thread**	20-40 mm (2-mm-Schritte)	5,0 mm	8-13 mm

Darüber hinaus sind sämtliche Schrauben selbstbohrend und selbstschneidend.
Des Weiteren wurde der Durchmesser des Führungsdrahtes vergrößert. Zur einfacheren Metallentfernung ist das distale Gewinde rückschneidend.

HBS2-Implantate

	Vorteil	*Nutzen*
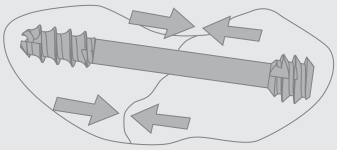	▪ Farbcodierte HBS2-Schrauben für jede Situation	▪ Sichere und effiziente Versorgung der verschiedensten Frakturen, auch bei sehr kleinen Fragmenten.<br>▪ Gezielte Beeinflussung der interfragmentären Kompression.<br>▪ Sichere Überbrückung des Frakturspalts bei bestmöglicher Abstützung im Knochen.
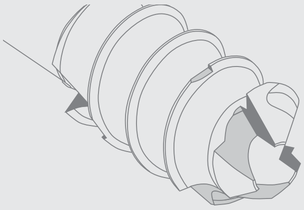	▪ Selbstbohrend und selbstschneidend	▪ Verkürzte und vereinfachte OP-Technik.<br>▪ Investition für Bohrer als Einwegartikel entfällt.
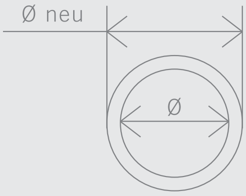	▪ Vergrößerter Führungsdraht-Durchmesser	▪ Noch bessere Führung durch einen um nahezu 20 % kräftigeren Führungsdraht.
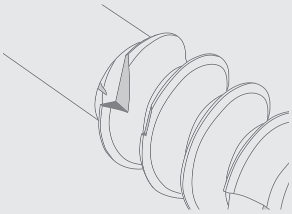	▪ Rückschneidendes Gewinde	▪ Zur einfacheren Entfernung der Schraube.
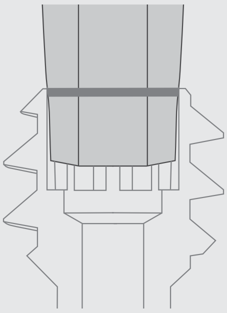	▪ T7 / T8 mit Selbsthaltefunktion	▪ Einfaches Entnehmen, Eindrehen und Entfernen der Schraube.

Merkmal, Funktion und Nutzen

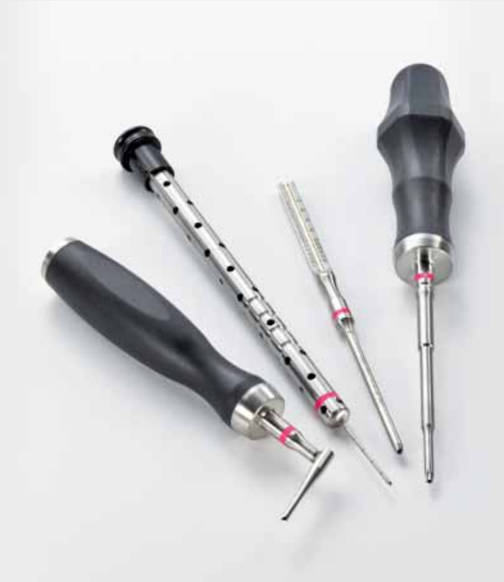

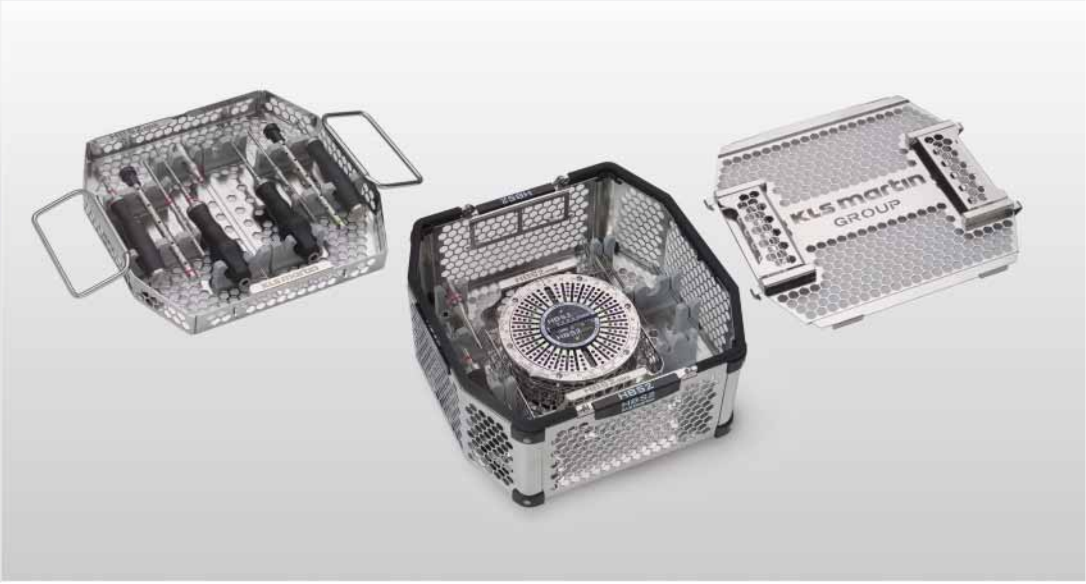

Das komplett neu gestaltete Instrumentarium ist für eine einfache und effiziente Handhabung farbcodiert. In der Regel kann die Versorgung mit nur 3 Instrumenten erfolgen. Für Ausnahmefälle stehen optional Zusatzinstrumente wie der Schließaufsatz zur Verfügung.

Bei der Gestaltung des Lagerungskonzepts waren zwei Aspekte wesentlich. Zum einen die einfache Handhabung, so sind die Instrumente entsprechend dem OP-Ablauf angeordnet. Zum anderen standen die Anforderungen an die Aufbereitung im Mittelpunkt.

HBS2-Instrumente und Lagerung

	Vorteil	*Nutzen*
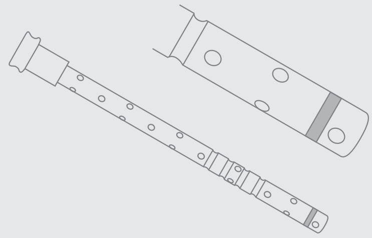	▪ Farbcodiertes Instrumentarium<br>- Midi (magenta)<br>- Mini (grün)	▪ Zur einfachen Identifizierung der jeweiligen Instrumente.
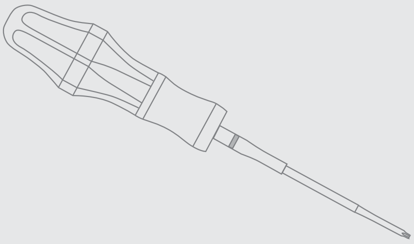	▪ Einteilige Instrumente mit ergonomisch geformten Silikonhandgriffen	▪ Gute taktile Rückmeldung.<br>▪ Keine Kupplungen, die zu Verwechslungen führen können.<br>▪ Keine Teile, die verloren gehen können.
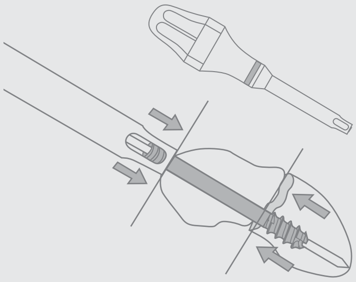	▪ Schließaufsatz	▪ Um einen großen verbliebenen Frakturspalt oder einen Spalt nach Spanimplantation zunächst schließen zu können.<br>▪ Frakturversorgung nach dem Prinzip der Zugschraubenosteo-synthese.
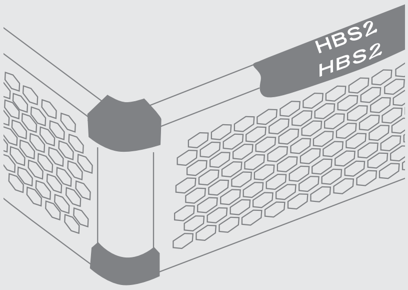	▪ Edelstahllagerung im Honig-wabendesign kombiniert mit Hochleistungskunststoff	▪ Hohe Stabilität bei geringem Gewicht.<br>▪ Gute Durchspülbarkeit durch große Öffnungen.<br>▪ Keine Wasserrückstände.<br>▪ Gute Ergonomie.
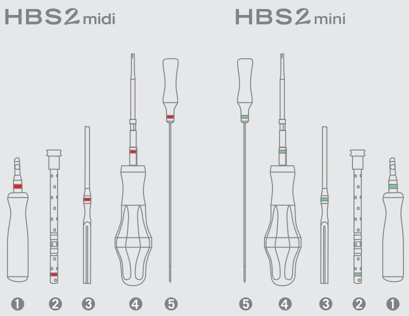	▪ Instrumente sind entsprechend dem OP-Ablauf angeordnet.	▪ Für eine einfache und effiziente Instrumentierung.

Schritt für Schritt
zur optimalen Versorgung

Indikationen

HBS2-Schrauben werden zur Versorgung von intraartikulären und extraartikulären Frakturen und Pseudarthrosen kleiner Knochen und Knochenfragmente verwendet sowie für Arthrodesen an kleinen Gelenken.

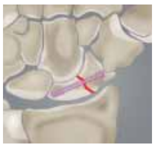

Skaphoidfraktur und Skaphoidpseudarthrose

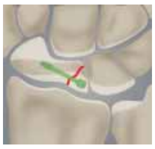

Proximale Polfraktur des Skaphoids

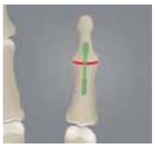

DIP-Arthrodesen

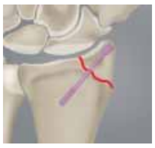

Fraktur des Processus styloideus radii

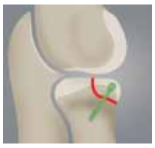

Proximale Radiuskopffraktur

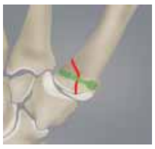

Metakarpalfrakturen

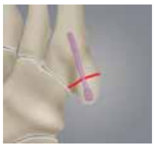

Metatarsalfrakturen

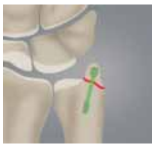

Fraktur des Processus styloideus ulnae

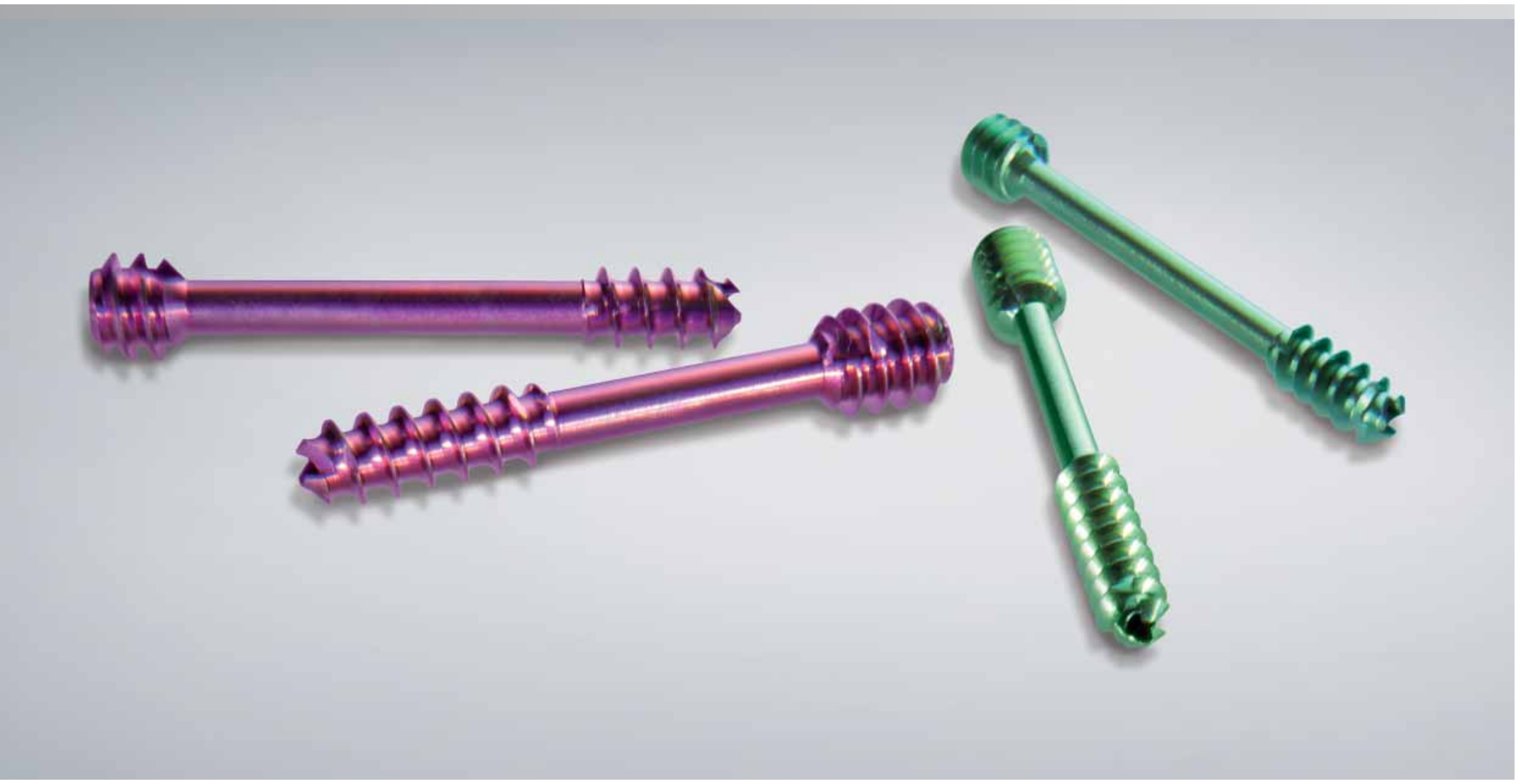

Operationstechniken

Skaphoidfraktur im mittleren Drittel

Minimalinvasive palmare OP-Technik *Seite 12-17*

Prof. Krimmer

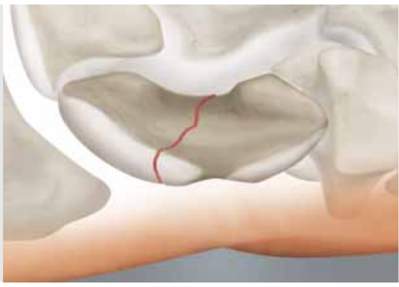

Skaphoidpseudarthrose

Offene palmare OP-Technik *Seite 18-25*

Prof. Krimmer

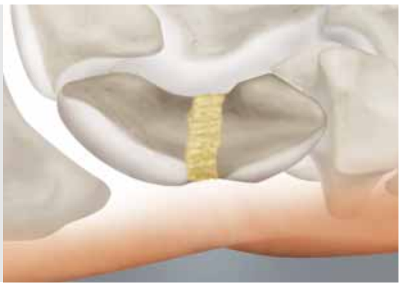

Skaphoidfraktur / Skaphoidpseudarthrose unter Verwendung des Schließaufsatzes

Offene palmare OP-Technik *Seite 26-29*

Prof. Krimmer

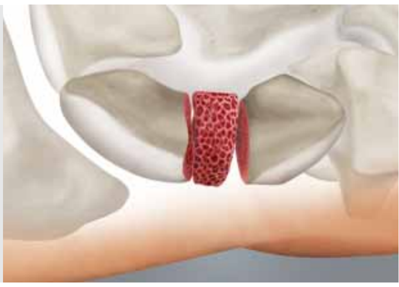

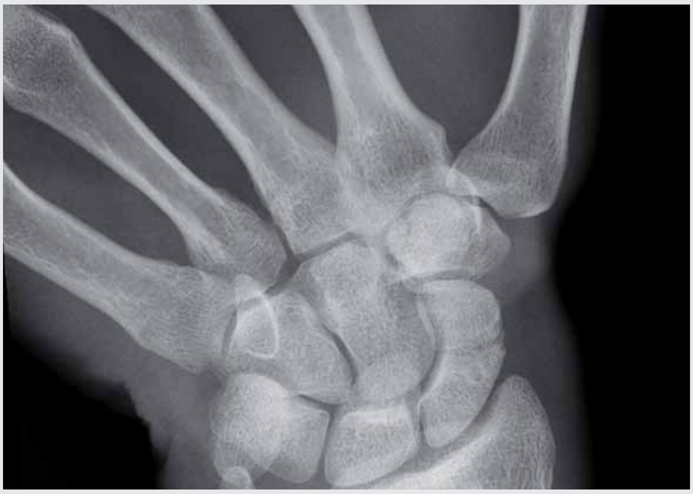

Quelle: Prof. Krimmer

Präoperative Planung

Neben der Durchführung von Standardaufnahmen im A/P- und seitlichen Strahlengang empfiehlt es sich, zusätzliche Aufnahmen in Faustschluss und Ulnarduktion (Stecher-Projektion) vorzunehmen. Eventuell kann eine zusätzliche Aufnahme in Hyperpronation notwendig sein.

Zur weiteren Abklärung sollte grundsätzlich eine hochauflösende Computertomographie durchgeführt werden.

Es ist jeweils darauf zu achten, dass die Bildgewinnung in der Längsachse des Skaphoids erfolgt.

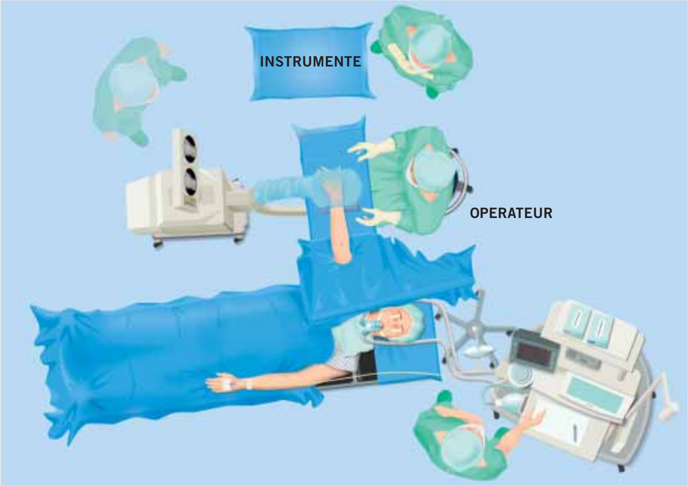

Lagerung des Patienten – minimalinvasiver palmarer Zugang

Der Patient wird in Rückenlage auf dem Operationstisch gelagert. Die zu operierende Hand wird in Überstreckung und Oberarmblutleere auf dem Handtisch positioniert.

Der Bildwandler wird gegenüber dem Operateur so platziert, dass er als Rechtshänder den Führungsdraht von distal nach proximal einbringen kann. Dies bedeutet, dass er am rechten Handgelenk auf der Kopfseite sitzt und beim linken auf der Fußseite. Dies erleichtert das korrekte Einbringen des Führungsdrahtes, da jederzeit Röntgenkontrollen durchgeführt werden können.

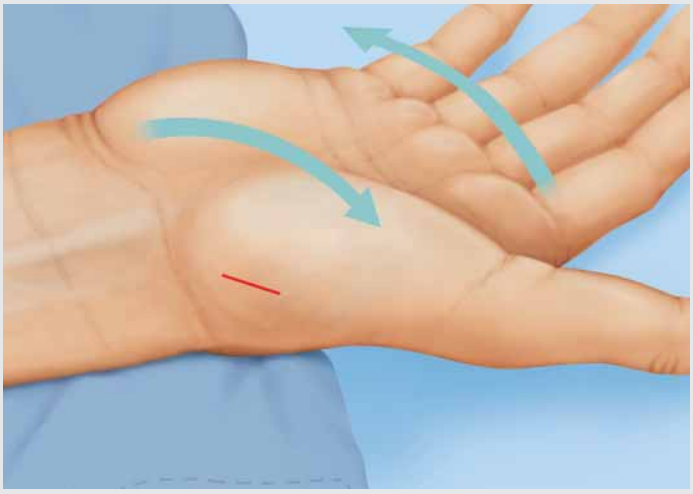

1. Minimalinvasiver palmarer Zugang

Auf Höhe des Skaphoid-Trapezio-Trapezoidal-Gelenks wird ein kurzer, schräger Hautschnitt durchgeführt.

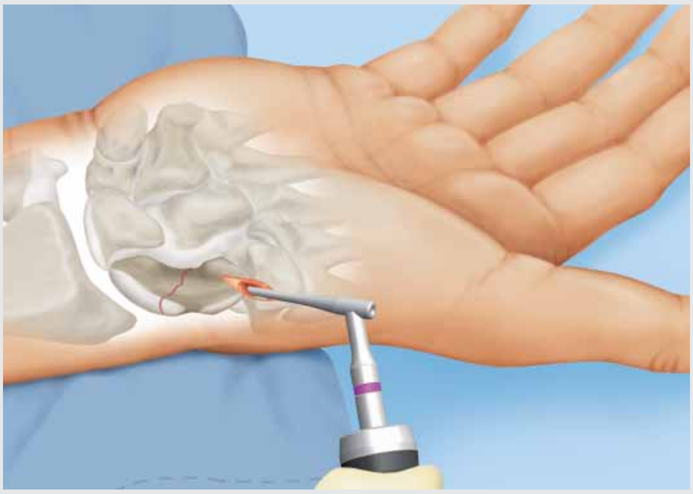

2. Positionierung der K-Draht-Führung

Die K-Draht-Führung wird auf dem Knochen positioniert.

Hinweis:
Eine radiale Positionierung der Einheit auf dem distalen Skaphoid erleichtert die korrekte Positionierung des Führungsdrahtes.

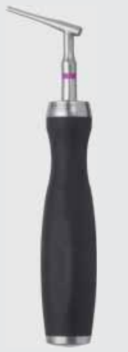

HBS2 midi
K-Draht-Führung

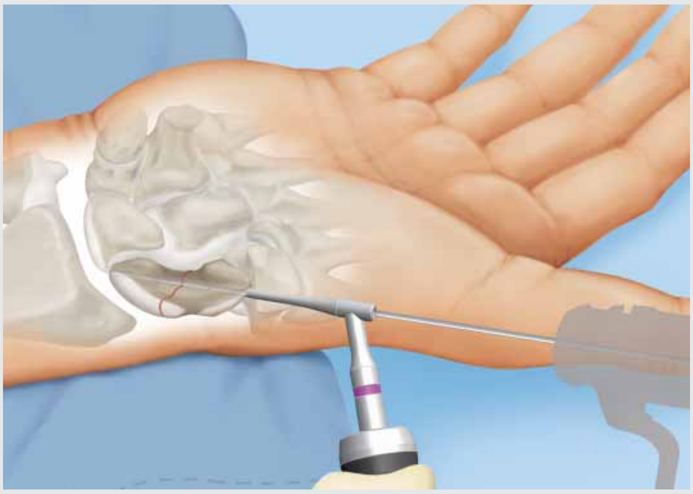

3. Einbringen des Führungsdrahts

Unter Bildwandlerkontrolle wird der Führungsdraht langsam eingebracht und optimal in der Längsachse, zentrisch in beiden Ebenen, im Knochen positioniert.

Die Spitze sollte in die gegenüberliegende Kortikalis ein-, diese aber nicht durchdringen.

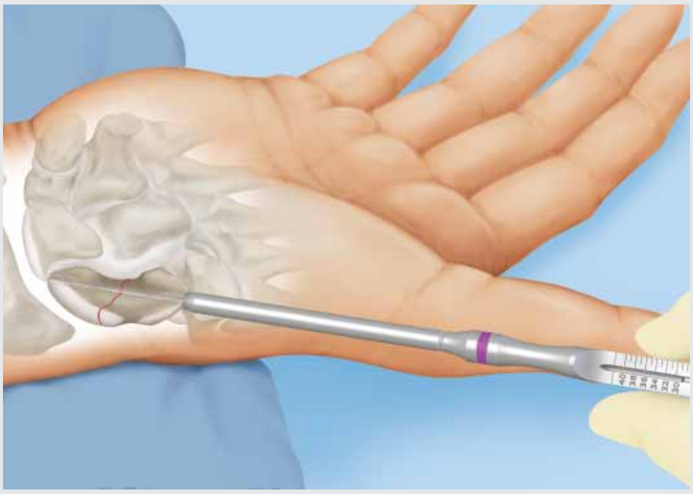

4. Längenbestimmung

Nach korrekter Positionierung des Führungsdrahtes wird die K-Draht-Führung entfernt und zur Längenbestimmung der Schraube die Messlehre über das hervorstehende Ende des Führungsdrahtes geschoben und direkt auf dem Knochen aufgesetzt.

Die Länge des eingebrachten Führungsdrahtanteils kann nun an der Skala abgelesen werden.

Zur Bestimmung der Schraubenlänge sind vom angezeigten Wert in Abhängigkeit vom Frakturspalt 2 - 4 mm abzuziehen.

Im Regelfall liegt die Schraubenlänge zwischen 22 und 26 mm.

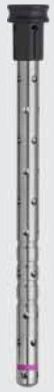

HBS2 midi
K-Draht-Spender,
∅ 1,1 mm

HBS2 midi
Führungsdraht,
∅ 1,1 mm, 125 mm

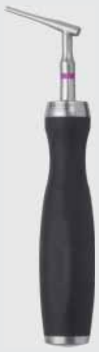

HBS2 midi
K-Draht-Führung

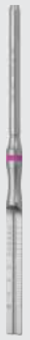

HBS2 midi
Messlehre

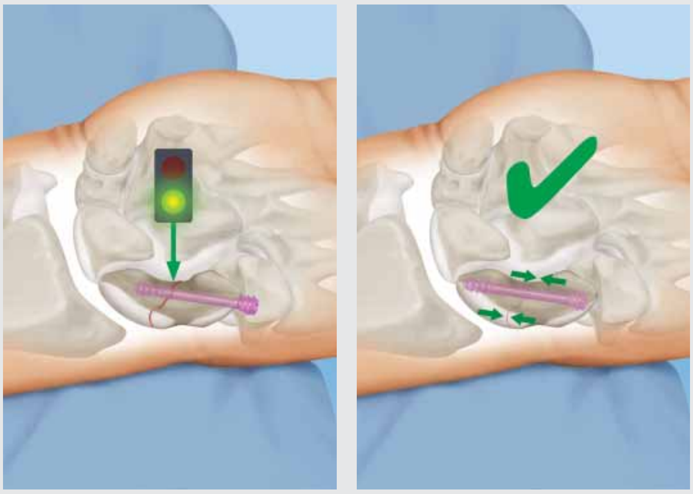

5. Auswahl der Schraube

Bei der Wahl des Schraubentyps ist die Position des Frakturspalts ausschlaggebend.

Das distale Gewinde der HBS2-Schraube muss den Frakturspalt komplett überbrückt haben, bevor das kurze proximale Gewinde im Knochen fasst. Nur so kann interfragmentäre Kompression erzielt werden.

Mittels der grünen Ampel wird dargestellt, wie eine korrekt gewählte HBS2-Schraube funktioniert. Die rote Ampel zeigt, was passiert, wenn eine Schraube mit zu langem Gewindeanteil gewählt wird.

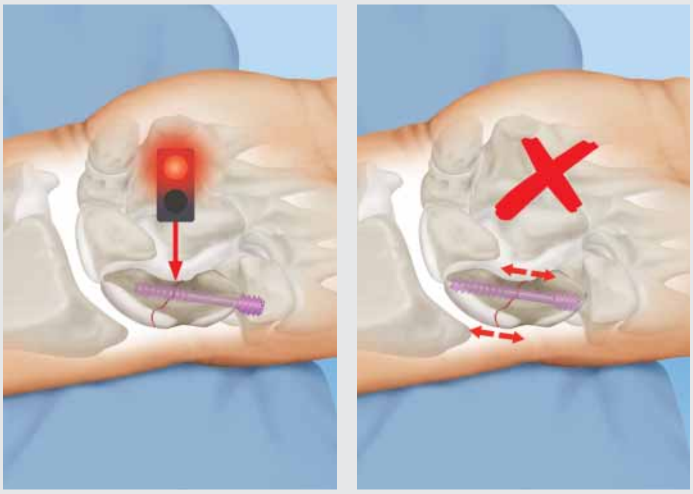

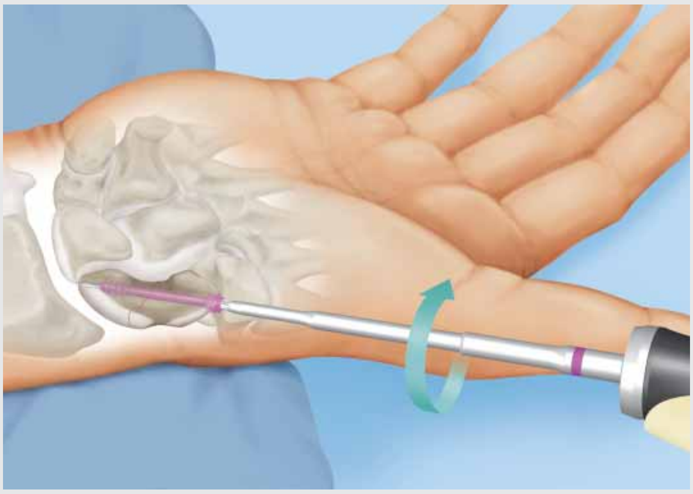

6. Einbringen der Schraube

Mit dem Schraubendreher wird nun eine Schraube passender Länge aus dem Rack entnommen und über den Führungsdraht implantiert.

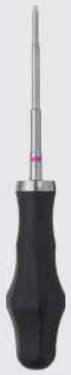

HBS2 midi
Schraubendreher T8

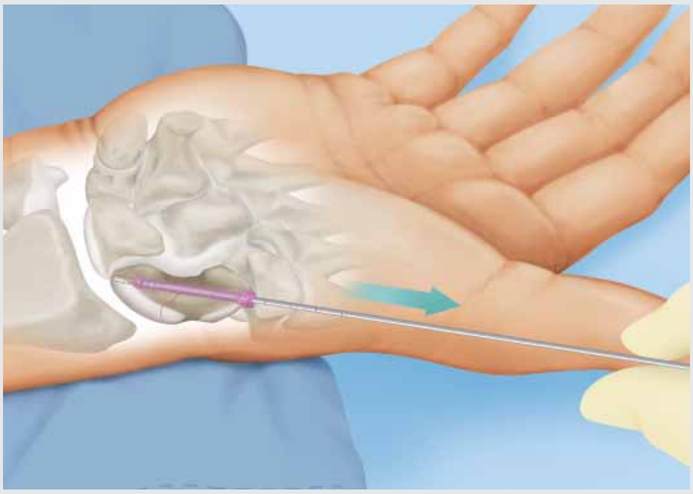

7. Entnahme des Führungsdrahtes

Sobald das distale Schraubengewinde den Frakturspalt überschritten hat (Röntgenkontrolle), sollte der Führungsdraht zurückgezogen werden, um ein Verkanten der Schraube gegen den Draht zu verhindern.

Mit dem Greifen des proximalen Gewindes wird Kompression auf die Fraktur ausgeübt. Die Kompression ist über die Länge des proximalen Gewindes und die unterschiedlichen Gewindesteigungen definiert.

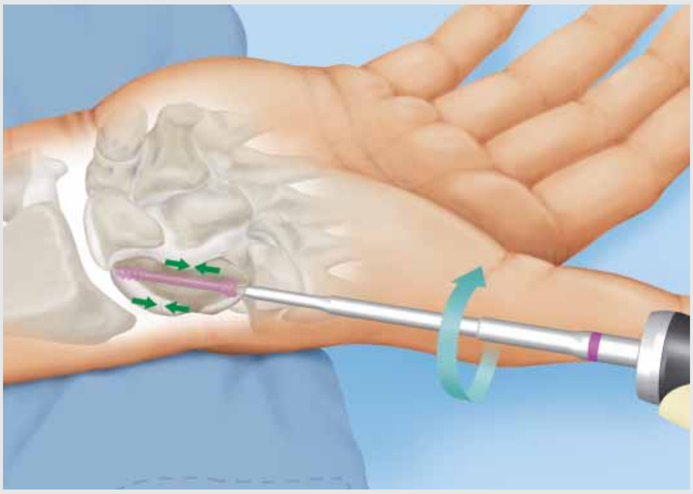

8. Finale Position der Schraube

Die Schraube wird nun noch ein bis zwei Umdrehungen eingedreht, damit das proximale Gewinde leicht unterhalb der Knochenoberfläche zum Liegen kommt.

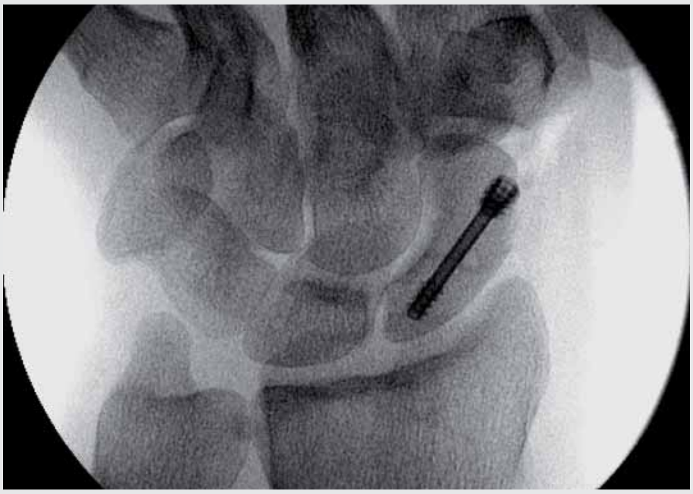

Quelle: Prof. Krimmer

Nachbehandlung

In Abhängigkeit der postoperativen Schmerzen wird für 2 Wochen ein elastisch polsternder Verband angelegt. Alternativ kann bei stärkerer Schmerzsymptomatik für 1 bis 2 Wochen eine palmare Schiene verwendet werden.

Die erste Kontrolle mit Röntgenaufnahmen im A/P- und seitlichen Strahlengang sowie in Stecherprojektion erfolgt nach 6 Wochen.

Im Zweifelsfall wird ergänzend eine hochauflösende Computertomographie in der Längsachse des Skaphoids durchgeführt.

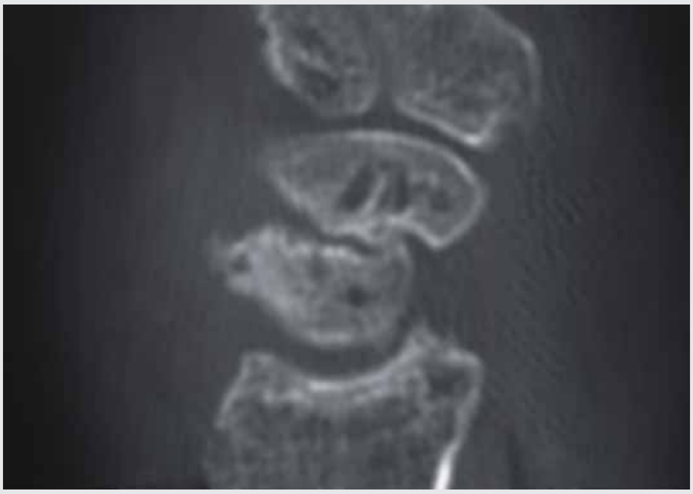

Quelle: Prof. Krimmer

Präoperative Planung

Neben der Durchführung von Standardaufnahmen im A/P- und seitlichen Strahlengang empfiehlt es sich, zusätzliche Aufnahmen in Faustschluss und Ulnarduktion (Stecher-Projektion) vorzunehmen. Eventuell kann eine zusätzliche Aufnahme in Hyperpronation notwendig sein.

Zur weiteren Abklärung sollte grundsätzlich eine hochauflösende Computertomographie durchgeführt werden.

Es ist jeweils darauf zu achten, dass die Bildgewinnung in der Längsachse des Skaphoids erfolgt.

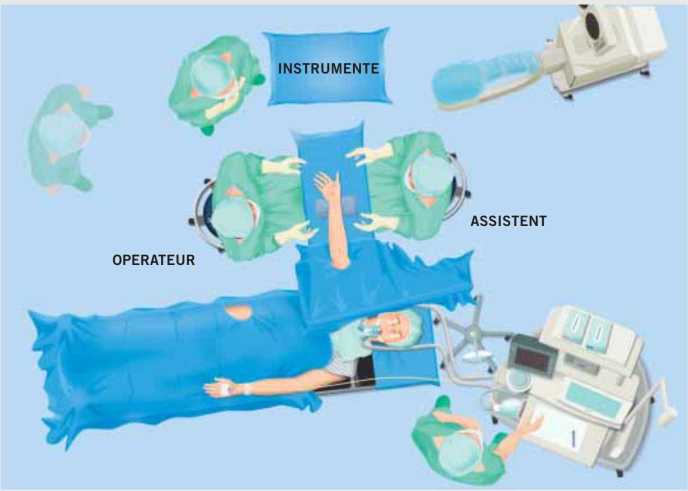

Lagerung des Patienten – offener palmarer Zugang

Der Patient wird in Rückenlage auf dem Operationstisch gelagert. Die zu operierende Hand wird in Überstreckung und Oberarmblutleere auf dem Handtisch positioniert.

Für den Fall, dass die Entnahme eines kortikospongiösen Spans aus dem Beckenkamm notwendig sein sollte, wird zusätzlich das Becken des Patienten vorbereitet und abgedeckt.

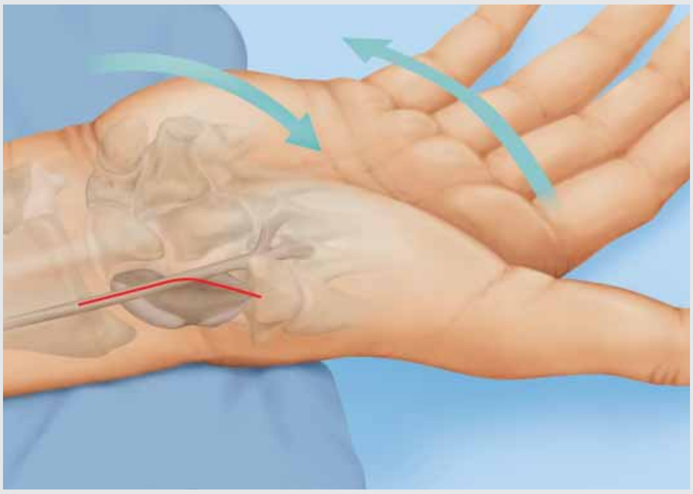

1. Offener palmarer Zugang

Beginnend am Tuberculum scaphoidei wird ein Hautschnitt in leicht gebogener Form entlang der FCR-Sehne durchgeführt.

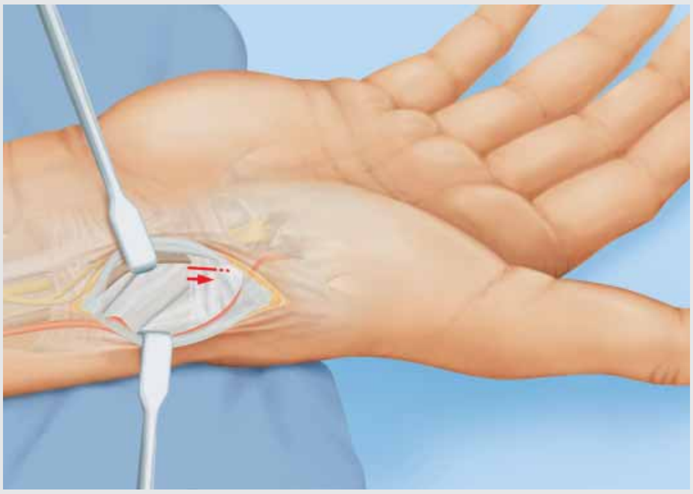

2. Offener palmarer Zugang

Die Sehnenscheide des Flexor carpi radialis wird isoliert und eröffnet und die FCR-Sehne nach ulnar retrahiert.

Der Schnitt wird nach distal bis zum Ramus palmaris superficialis der Arteria radialis fortgeführt.

Gegebenenfalls muss der Ast der Arteria radialis ligiert werden.

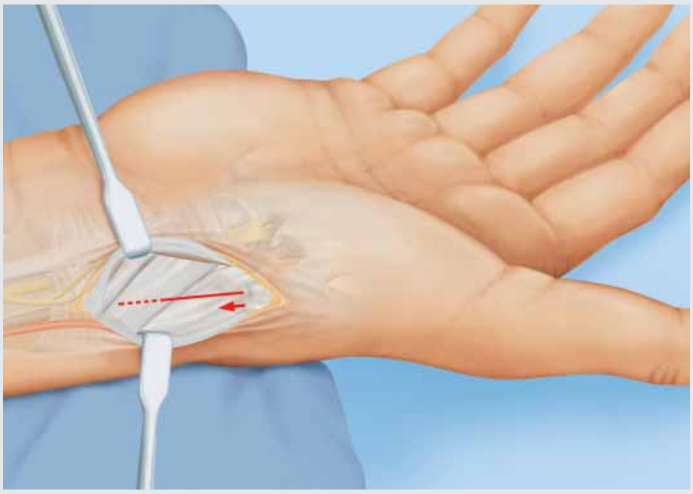

3. Offener palmarer Zugang

Danach wird distal das STT-Gelenk lokalisiert und quer eröffnet.

Die seitlichen Kapsel-Band-Strukturen sollten erhalten bleiben, da hierüber der wesentliche Anteil der arteriellen Blutversorgung des Skaphoids erfolgt.

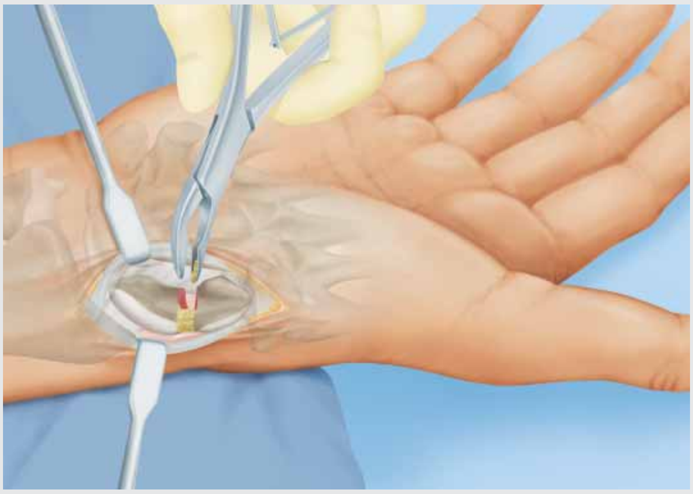

4. Ausräumen der Pseudarthrose

Die Pseudarthrose wird in der Regel mit einem Luer großzügig ausgeräumt. Sollte der Knochen besonders hart sein, kann ein Meißel verwendet werden.

Die Resektion sollte so weit erfolgen, bis spongiöse Knochenstruktur erkennbar ist.

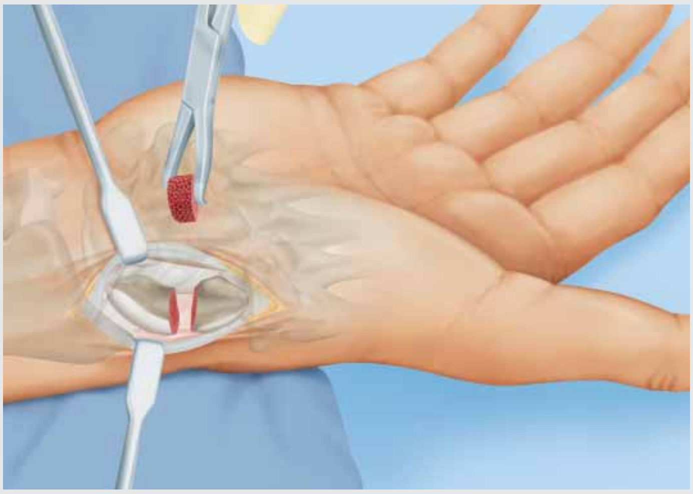

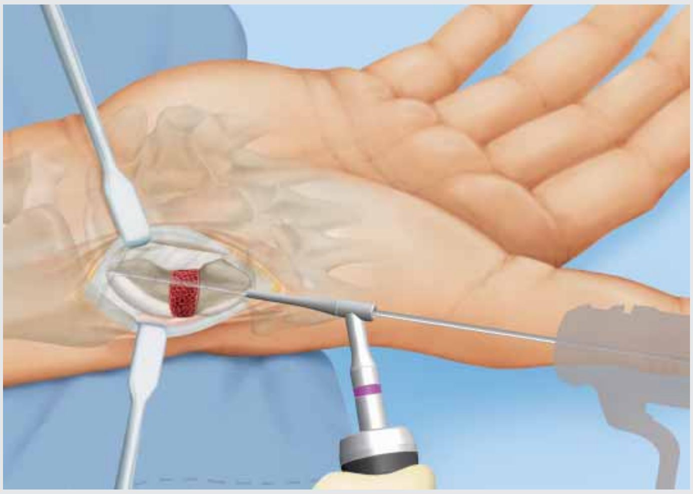

5. Implantation eines Knochenspans

Nachdem die Pseudarthrose ausgeräumt ist, wird ein kortiko-spongiöser Knochenspan aus dem Beckenkamm entnommen. Bevorzugt kommt hierbei die Beckenkammspongiosaentnahme-fräse (Art.-Nr. 23-190-05-07 oder 23-190-06-07) zur Anwendung. Die Operationszeit ist hierbei verkürzt und die Morbidität deutlich geringer als bei herkömmlichen Techniken.

Durch diese Technik ist die Spongiosa verdichtet und damit stabil, so dass beide Kortikali entfernt werden können, mit dem Vorteil der begünstigten Gefäßeinsprossung während der Knochenheilung.

Nun kann der Knochenspan eingesetzt werden. Im Falle einer „Humpback“-Deformität ist darauf zu achten, dass die Aufrichtung des distalen Fragments mit der anatomischen Rekonstruktion des Skaphoids einhergeht.

Bei sehr ausgedehnten Defekten ist die Stabilisierung durch einen temporären K-Draht hilfreich, der möglichst auf der ulnaren Seite eingebracht werden sollte, damit er nicht mit dem Führungsdraht kollidiert.

6. Einbringen des Führungsdrahts

Unter Bildwandlerkontrolle wird der Führungsdraht langsam eingebracht und optimal in der Längsachse, zentrisch in beiden Ebenen, im Knochen positioniert.

Die Spitze sollte in die gegenüberliegende Kortikalis ein-, diese aber nicht durchdringen.

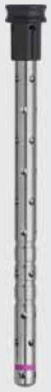

HBS2 midi
K-Draht-Spender,
∅ 1,1 mm

HBS2 midi
Führungsdraht,
∅ 1,1 mm, 125 mm

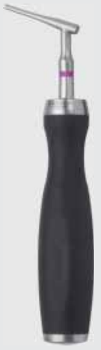

HBS2 midi
K-Draht-Führung

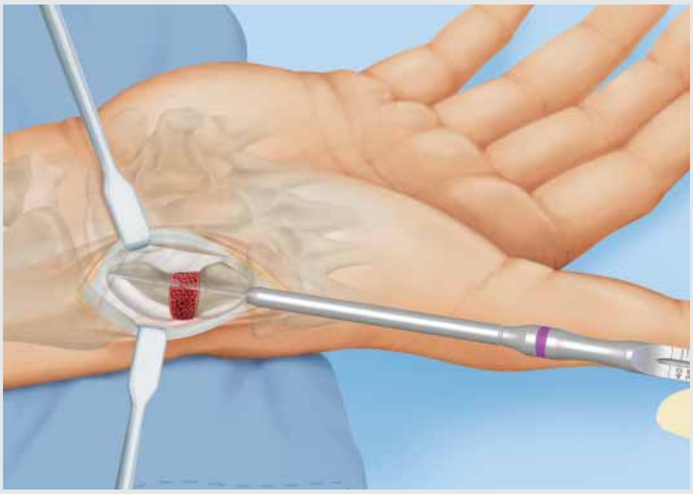

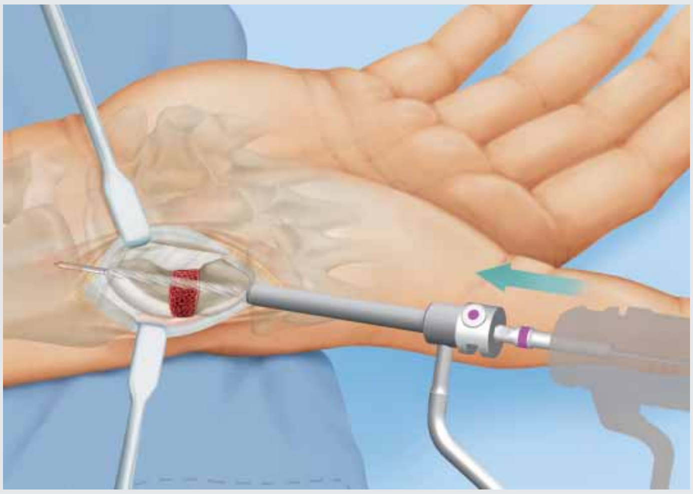

7. Längenbestimmung

Nach korrekter Positionierung des Führungsdrahtes wird die K-Draht-Führung entfernt und zur Längenbestimmung der Schraube die Messlehre über das hervorstehende Ende des Führungsdrahtes geschoben und direkt auf dem Knochen aufgesetzt.

Die Länge des eingebrachten Führungsdrahtanteils kann nun an der Skala abgelesen werden.

Zur Bestimmung der Schraubenlänge sind vom angezeigten Wert ca. 2 mm abzuziehen, sofern der kortikospongiöse Knochenspan press-fit eingebracht wurde.

Im Regelfall liegt die Schraubenlänge zwischen 22 und 26 mm.

8. Vorbohren

Bei der Versorgung von länger zurückliegenden Skaphoidpseudarthrosen mit sklerosiertem proximalen Fragment wird empfohlen, mit dem kanülierten Bohrer ohne Stopp unter Bildwandlerkontrolle vorsichtig über den Führungsdraht vorzubohren, da hier eine besonders harte Knochenstruktur vorliegt.

Alternativ kann, wie abgebildet, mit dem kanülierten Bohrer mit Stopp in Verbindung mit der Gewebeschutzhülse aufgebohrt werden. Hierzu wird mit Hilfe des Tiefenanschlags die zuvor gemessene Länge am Bohrer eingestellt. Der Bohrer wird nun entlang des Führungsdrahtes unter Bildwandlerkontrolle so weit eingebracht, bis der Tiefenanschlag auf der Gewebeschutzhülse aufsitzt.

Hinweis:
Um ein eventuelles Herausfallen des Führungsdrahtes nach dem Bohren zu verhindern, kann dieser vor dem Aufbohren weiter bis in den distalen Radius vorgeschoben werden. Nach diesem Arbeitsschritt sollte das Handgelenk nicht mehr weiter bewegt werden, um ein Abbrechen des Drahtes zu verhindern.

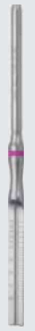

HBS2 midi
Messlehre

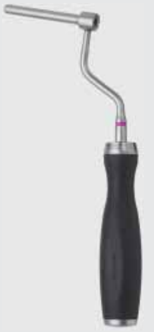

HBS2 midi
Gewebeschutzhülse

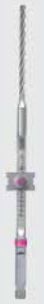

HBS2 midi
Spiralbohrer mit Stopp, kanüliert, AO-Anschluss, Ø 2,3 / 1,1 mm

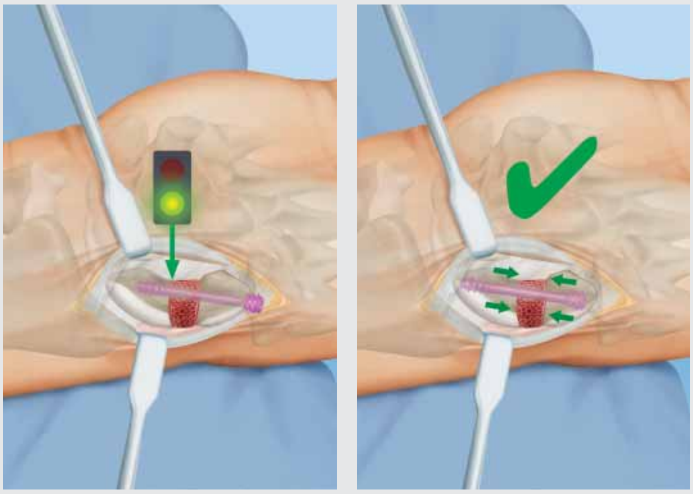

9. Auswahl der Schraube

Bei der Wahl des Schraubentyps ist die Position der Pseudarthrose beziehungsweise des Knochenspans ausschlaggebend.

Das distale Gewinde der HBS2-Schraube muss die Pseudarthrosenzone komplett überbrückt haben, bevor das kurze proximale Gewinde im Knochen fasst. Nur so kann interfragmentäre Kompression erzielt werden.

Mittels der grünen Ampel wird dargestellt, wie eine korrekt gewählte HBS2-Schraube funktioniert. Die rote Ampel zeigt, was passiert, wenn eine Schraube mit zu langem Gewindeanteil gewählt wird.

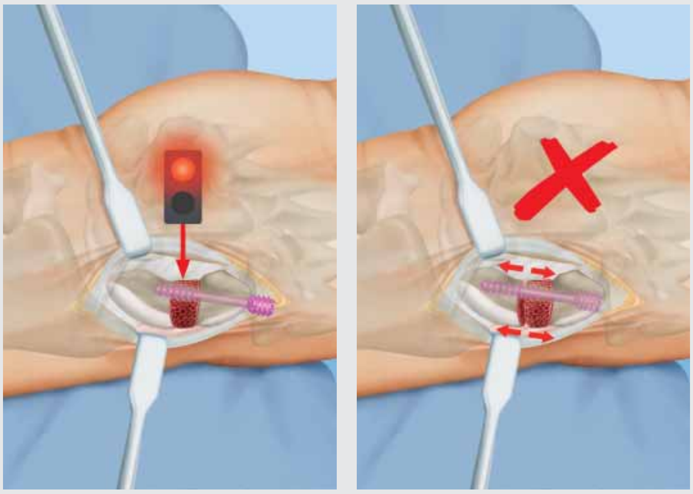

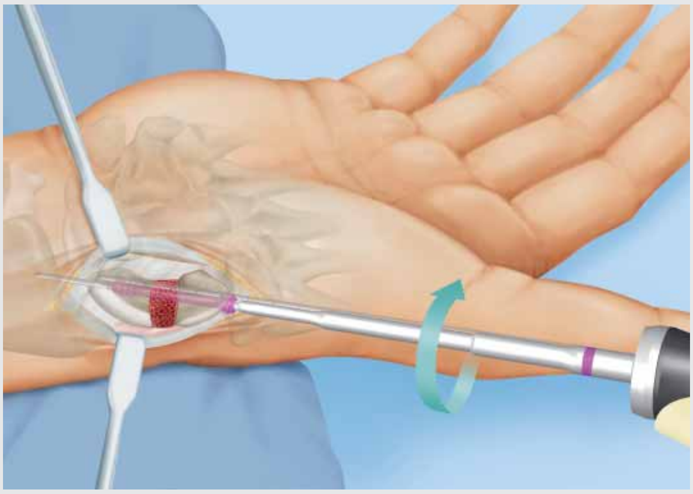

10. Einbringen der Schraube

Mit dem Schraubendreher wird nun eine Schraube passender Länge aus dem Rack entnommen und über den Führungsdraht implantiert.

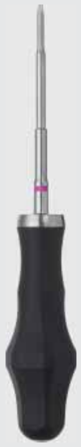

HBS2 midi
Schraubendreher T8

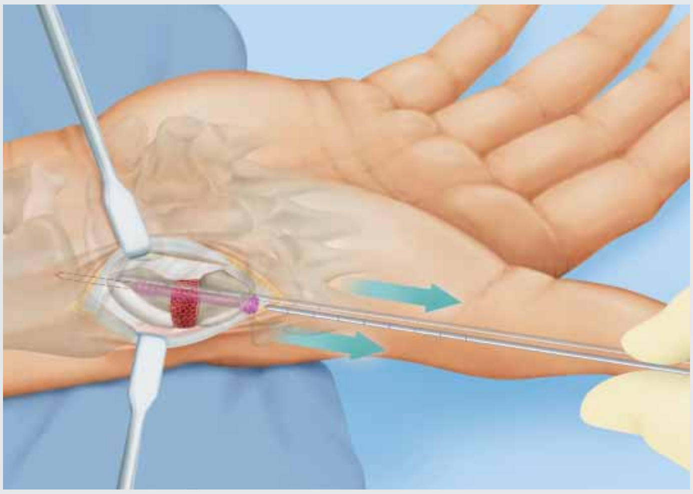

11. Entnahme des Führungsdrahtes

Sobald das distale Schraubengewinde den Knochenspan überschritten hat (Röntgenkontrolle), sollte der Führungsdraht zurückgezogen werden, um ein Verkanten der Schraube gegen den Draht zu verhindern. Gegebenenfalls wird nun auch der Antirotationsdraht entfernt.

Mit dem Greifen des proximalen Gewindes wird Kompression auf die Fraktur ausgeübt. Die Kompression ist über die Länge des proximalen Gewindes und die unterschiedlichen Gewindesteigungen klar definiert.

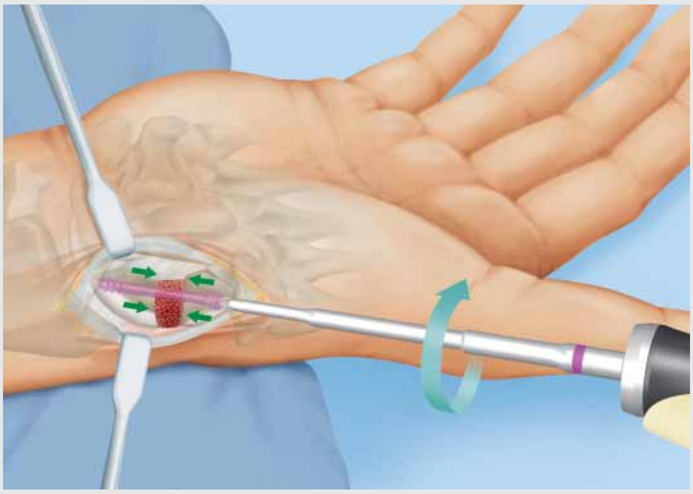

12. Finale Position der Schraube

Die Schraube wird nun noch ein bis zwei Umdrehungen eingedreht, damit das proximale Gewinde leicht unterhalb der Knochenoberfläche zum Liegen kommt.

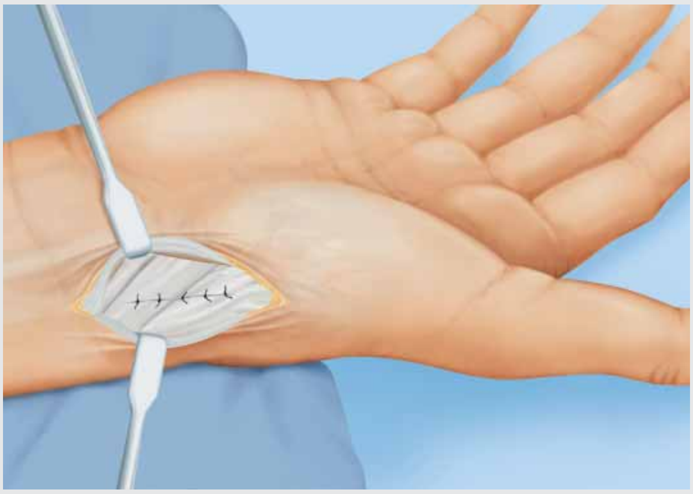

13. Wundverschluss

Es sollte ein sorgfältiger Wundverschluss erfolgen, mit exakter Adaptation der Kapsel-Band-Struktur und gesondertem Verschluss des vorderen Sehnenscheidenblattes der FCR-Sehne, um Vernarbungen vorzubeugen, die zur Bewegungseinschränkung führen können.

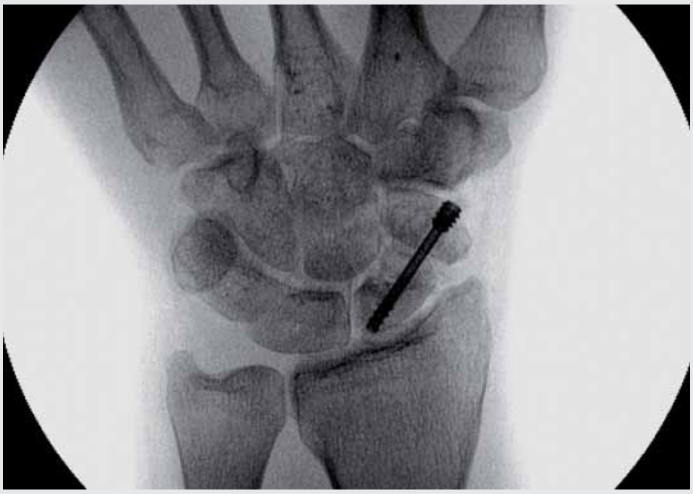

Quelle: Prof. Krimmer

Nachbehandlung

Nach dem Eingriff erfolgt eine Gipsruhigstellung für 6 Wochen.

Die erste Kontrolle mit Röntgenaufnahmen im A/P- und seitlichen Strahlengang sowie in Stecherprojektion erfolgt nach 6 Wochen.

Im Zweifelsfall wird ergänzend eine hochauflösende Computertomographie in der Längsachse des Skaphoids durchgeführt.

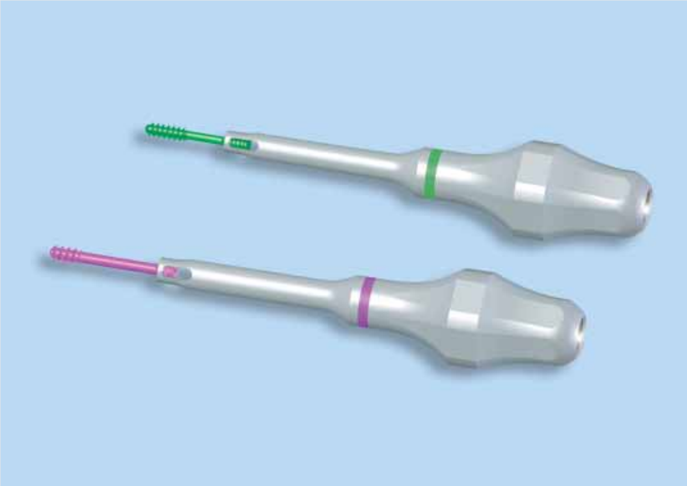

1. Schließen des Frakturspalts oder der Pseudarthrosenzone

Sollte der Frakturspalt besonders groß sein oder die Pseudarthrosenzone nach der Spanimplantation einen Spalt aufweisen, wird empfohlen, diesen zunächst mit dem Schließaufsatz zu schließen.

Die HBS2-Schraube wird über das proximale Gewinde mit dem Schließaufsatz verbunden. Dabei schließt das proximale Gewinde der HBS2-Schraube bündig mit dem Schließaufsatz ab.

Hinweis:
Das analoge Instrumentarium steht auch für die HBS2 mini zur Verfügung.

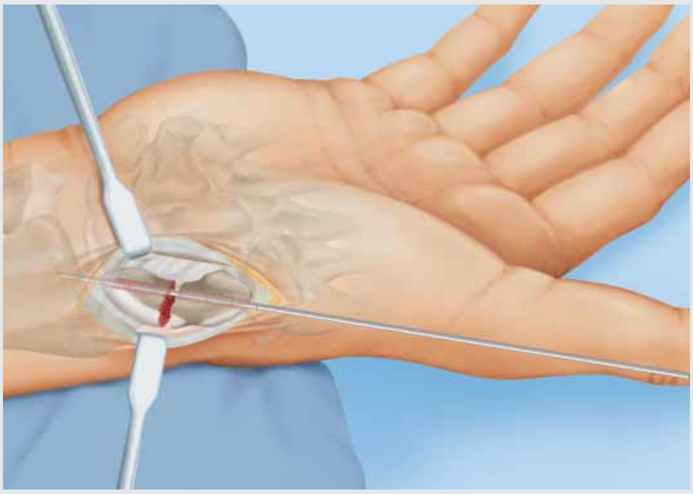

2. Einbringen des Führungsdrahtes, Längenbestimmung und Vorbohren

Wie auf Seite 21 und 22 beschrieben, wird auch bei Verwendung des Schließaufsatzes der Führungsdraht eingebracht und die Länge der Schraube bestimmt.

Um den Widerstand beim Eindrehen der Schraube mit dem Schließaufsatz möglichst gering zu halten, wird empfohlen vorzubohren, wie auf Seite 22 beschrieben.

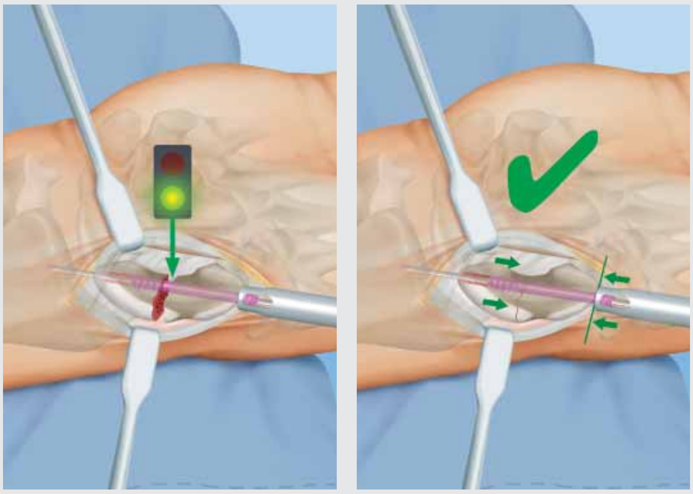

3. Auswahl der Schraube

Bei der Wahl des Schraubentyps ist die Position des Frakturspalts beziehungsweise der Pseudarthrosenzone ausschlaggebend.

Insbesondere bei Verwendung des Schließaufsatzes ist darauf zu achten, dass das distale Gewinde der HBS2-Schraube entweder den Frakturspalt oder die Pseudarthrosenzone komplett überbrückt hat, bevor das kurze proximale Gewinde im Knochen fasst. Nur so kann zunächst der verbliebene Spalt geschlossen und anschließend interfragmentäre Kompression erzielt werden.

Mittels der grünen Ampel wird dargestellt, wie eine korrekt gewählte HBS2-Schraube in Verbindung mit dem Schließaufsatz funktioniert. Die rote Ampel zeigt, was passiert, wenn eine Schraube mit zu langem Gewindeanteil gewählt wird.

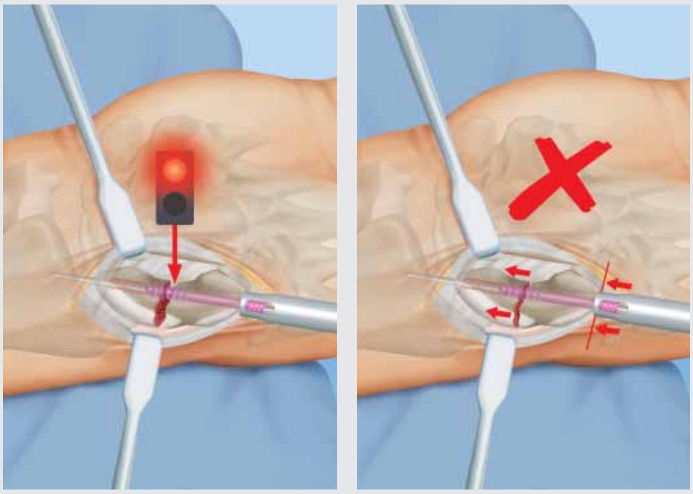

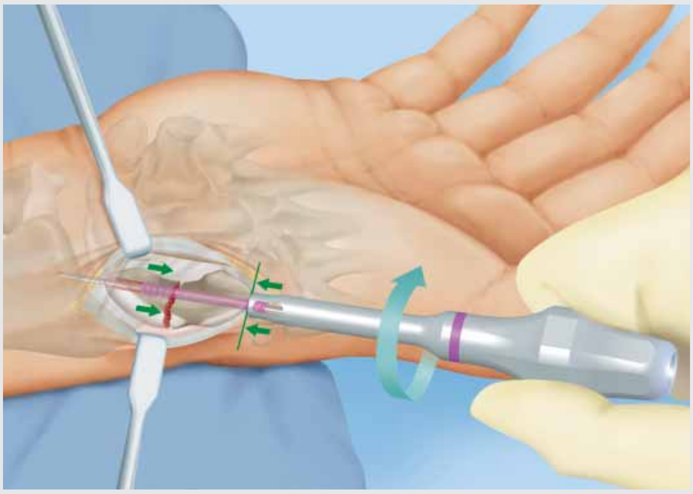

4. Zugschraubenosteosynthese mit dem Schließaufsatz

Dadurch, dass sich der Schließaufsatz auf dem Kahnbein stützt, bildet sich ein Gegenlager, und der verbliebene Spalt kann nach dem Prinzip der Zugschraubenosteosynthese unter Sichtkontrolle geschlossen werden.

Hinweis:
Mit dieser Technik kann auch an anderen Stellen das Zugschraubenprinzip mit dem Vorteil des versenkten Schraubenkopfes in idealer Weise realisiert werden.

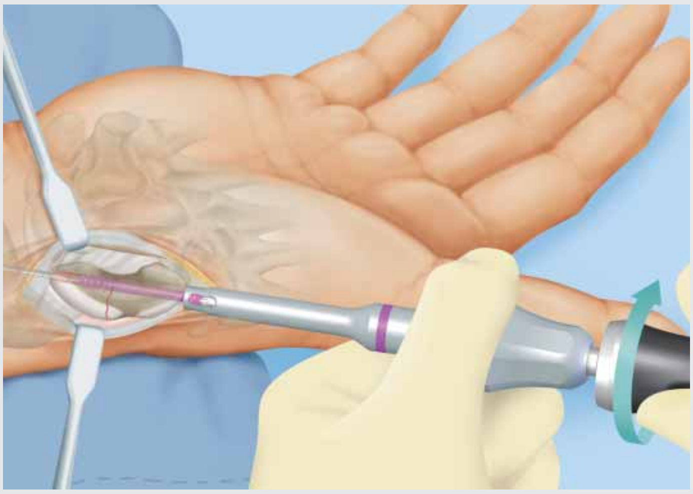

5. Einbringen der Schraube

Nachdem der Spalt mit dem Schließaufsatz geschlossen wurde, wird die HBS2-Schraube mit dem Schraubendreher durch die Kanülierung des Schließaufsatzes in die finale Position gebracht.

Mit dem Greifen des proximalen Gewindes im Skaphoid wird definiert interfragmentäre Kompression aufgebracht. Die Kompression ist über die Länge des proximalen Gewindes und die unterschiedlichen Gewindesteigungen klar definiert.

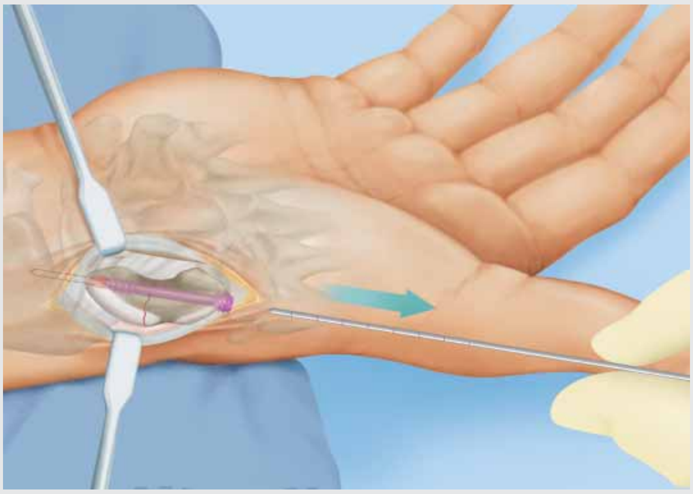

6. Entnahme des Führungsdrahtes

Sobald das proximale Gewinde greift, sollte der Führungsdraht und der Schließaufsatz entfernt werden.

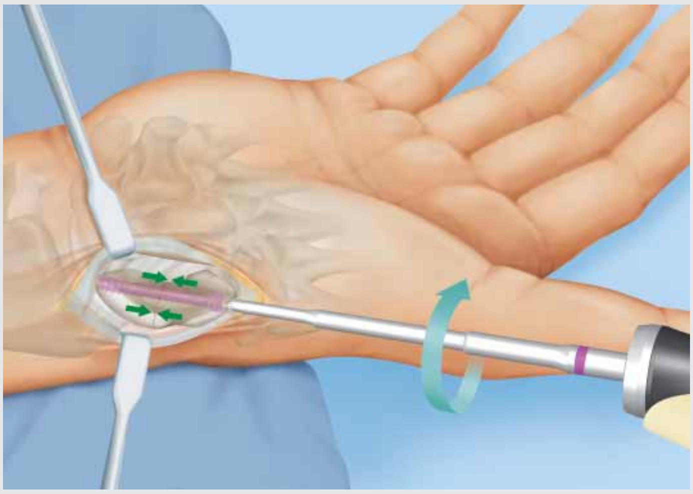

7. Finale Position der Schraube

Die Schraube wird nun noch ein bis zwei Umdrehungen eingedreht, damit das proximale Gewinde leicht unterhalb der Knochenoberfläche zum Liegen kommt.

Implantate, Instrumente und Lagerung
HBS2

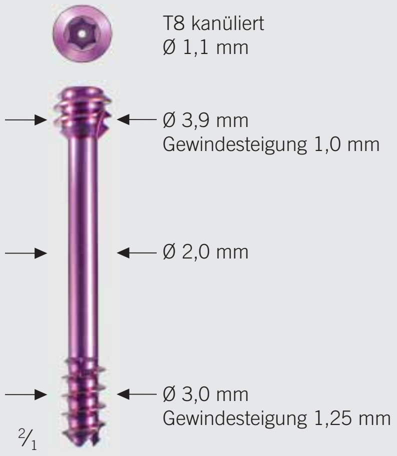

HBS2 midi short thread

Ti

1 unit(s)

Gewindelänge proximal
p = 3,5 mm

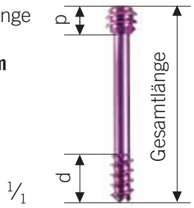

Gesamtlänge (mm)	Art.-Nr.	Gewindelänge distal d (mm)
10	26-800-10-09	4,0
11	26-800-11-09	4,0
12	26-800-12-09	4,0
13	26-800-13-09	4,0
14	26-800-14-09	5,0
15	26-800-15-09	5,0
16	26-800-16-09	5,0
17	26-800-17-09	5,0
18	26-800-18-09	5,0
19	26-800-19-09	5,0
20	26-800-20-09	5,0
21	26-800-21-09	6,0
22	26-800-22-09	6,0
23	26-800-23-09	6,0
24	26-800-24-09	6,0
25	26-800-25-09	6,0
26	26-800-26-09	6,0
27	26-800-27-09	6,0
28	26-800-28-09	6,0
29	26-800-29-09	6,0
30	26-800-30-09	6,0

HBS2 midi long thread

Ti

1 unit(s)

Gewindelänge proximal
p = 5 mm

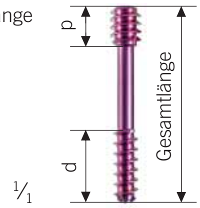

Gesamtlänge (mm)	Art.-Nr.	Gewindelänge distal d (mm)
20	26-810-20-09	9,0
22	26-810-22-09	9,0
24	26-810-24-09	9,0
26	26-810-26-09	10,0
28	26-810-28-09	10,0
30	26-810-30-09	11,0
32	26-810-32-09	11,0
34	26-810-34-09	12,0
36	26-810-36-09	12,0
38	26-810-38-09	12,0
40	26-810-40-09	13,0

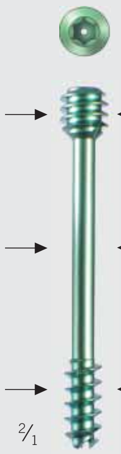

Icon-Erläuterungen

- Ti – TiAl6V4
- T-Drive kanüliert
- 1 unit(s) – Verpackungseinheit

HBS2 mini short thread

Gewindelänge proximal
p = 3,5 mm

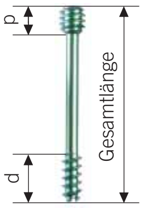

Gesamtlänge (mm)	Art.-Nr.	Gewindelänge distal d (mm)
10	26-820-10-09	4,0
11	26-820-11-09	4,0
12	26-820-12-09	4,0
13	26-820-13-09	4,0
14	26-820-14-09	5,0
15	26-820-15-09	5,0
16	26-820-16-09	5,0
17	26-820-17-09	5,0
18	26-820-18-09	5,0
19	26-820-19-09	5,0
20	26-820-20-09	5,0
21	26-820-21-09	6,0
22	26-820-22-09	6,0
23	26-820-23-09	6,0
24	26-820-24-09	6,0
25	26-820-25-09	6,0
26	26-820-26-09	6,0
27	26-820-27-09	6,0
28	26-820-28-09	6,0
29	26-820-29-09	6,0
30	26-820-30-09	6,0

HBS2 mini long thread

Gewindelänge proximal
p = 5 mm

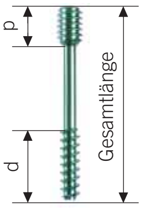

Gesamtlänge (mm)	Art.-Nr.	Gewindelänge distal d (mm)
20	26-830-20-09	9,0
22	26-830-22-09	9,0
24	26-830-24-09	9,0
26	26-830-26-09	10,0
28	26-830-28-09	10,0
30	26-830-30-09	11,0
32	26-830-32-09	11,0
34	26-830-34-09	12,0
36	26-830-36-09	12,0
38	26-830-38-09	12,0
40	26-830-40-09	13,0

Instrumente
HBS2 midi

HBS2 midi
Standardinstrumentarium:

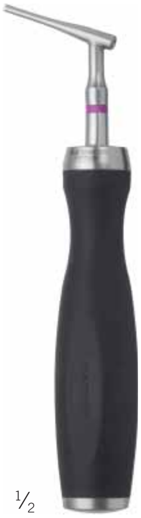
½

26-850-02-07
K-Draht-Führung
15 cm/6"

St Sic 1 unit(s)

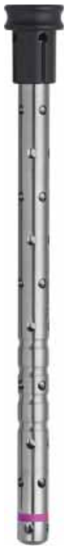
½

26-850-13-07
K-Draht-Spender
15 cm/6"
∅ 1,1 mm

St 1 unit(s)

½

26-850-00-05
Führungsdraht
125 mm
∅ 1,1 mm

St 10 unit(s)

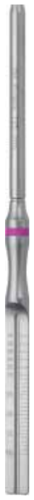
½

26-850-06-07
Messlehre

St 1 unit(s)

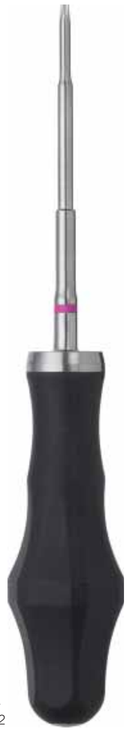
½

26-850-17-07
Schraubendreher T8

St Sic 1 unit(s)

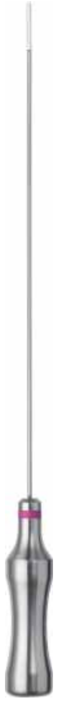
½

26-850-03-07
Reinigungs-Draht

St 1 unit(s)

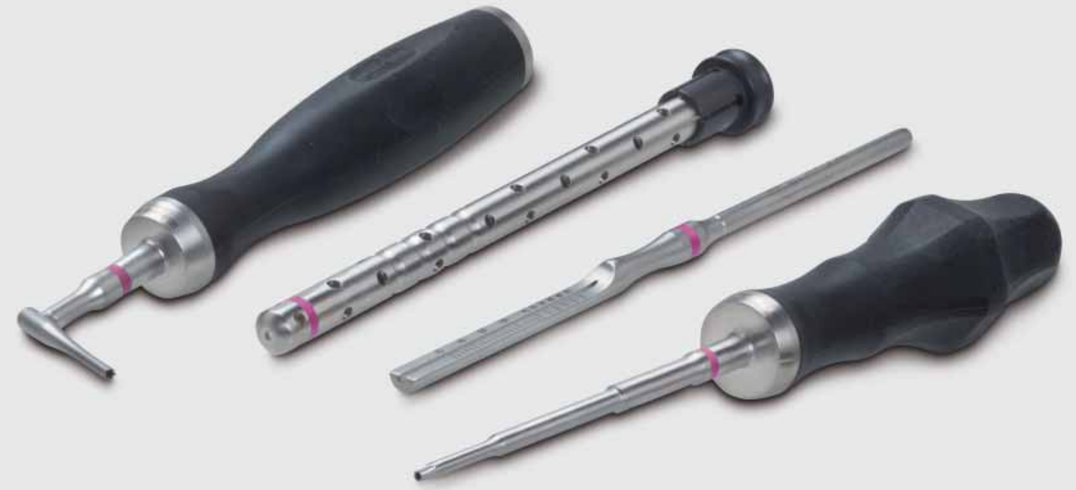

Icon-Erläuterungen

- HBS2 midi
- St Stahl
- Sic Silikon
- T-Drive kanüliert
- 1 unit(s) Verpackungseinheit

HBS2 midi
Optionale Instrumente:

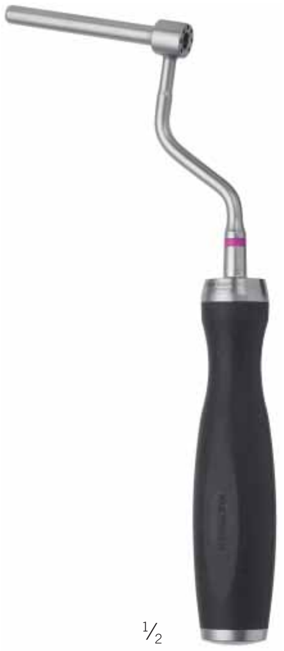

½

26-850-01-07
Gewebeschutzhülse

St Sic 1 unit(s)

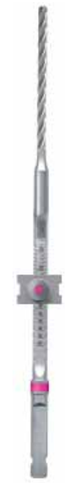

½

26-850-19-09
Spiralbohrer
mit Stopp, kanüliert,
AO-Anschluss
Ø 2,3 / 1,1 mm

St 1 unit(s)

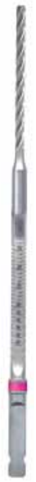

½

26-850-20-09
Spiralbohrer
ohne Stopp, kanüliert
AO-Anschluss
Ø 2,3 / 1,1 mm

St 1 unit(s)

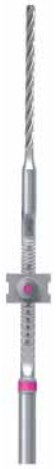

½

26-850-09-09
Spiralbohrer
mit Stopp, kanüliert,
zylindrisch
Ø 2,3 / 1,1 mm

St 1 unit(s)

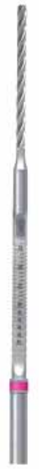

½

26-850-10-09
Spiralbohrer
ohne Stopp, kanüliert,
zylindrisch
Ø 2,3 / 1,1 mm

St 1 unit(s)

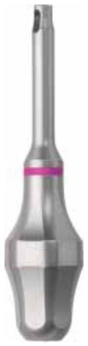

½

26-850-22-07
Schließaufsatz

St 1 unit(s)

Instrumente
HBS2 mini

HBS2 midi
Standardinstrumentarium:

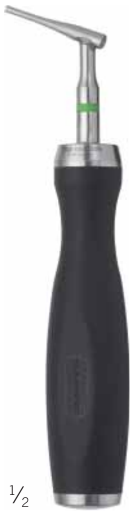

½

26-875-02-07
K-Draht-Führung
15 cm/6"

St Sic 1 unit(s)

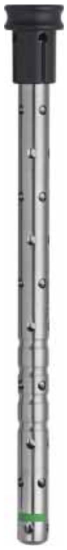

½

26-875-13-07
K-Draht-Spender
15 cm/6"
∅ 0,9 mm

St 1 unit(s)

½

26-875-00-05
Führungsdraht
125 mm
∅ 0,9 mm

St 10 unit(s)

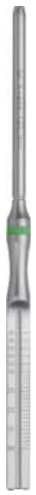

½

26-875-06-07
Messlehre

St 1 unit(s)

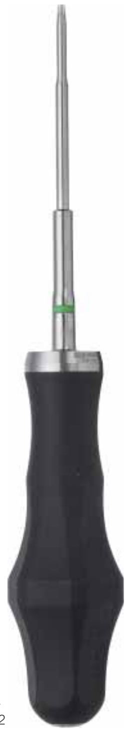

½

26-875-17-07
Schraubendreher
T6/T7 kanüliert

St Sic 1 unit(s)

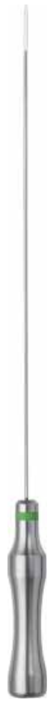

½

26-875-03-07
Reinigungs-Draht

St 1 unit(s)

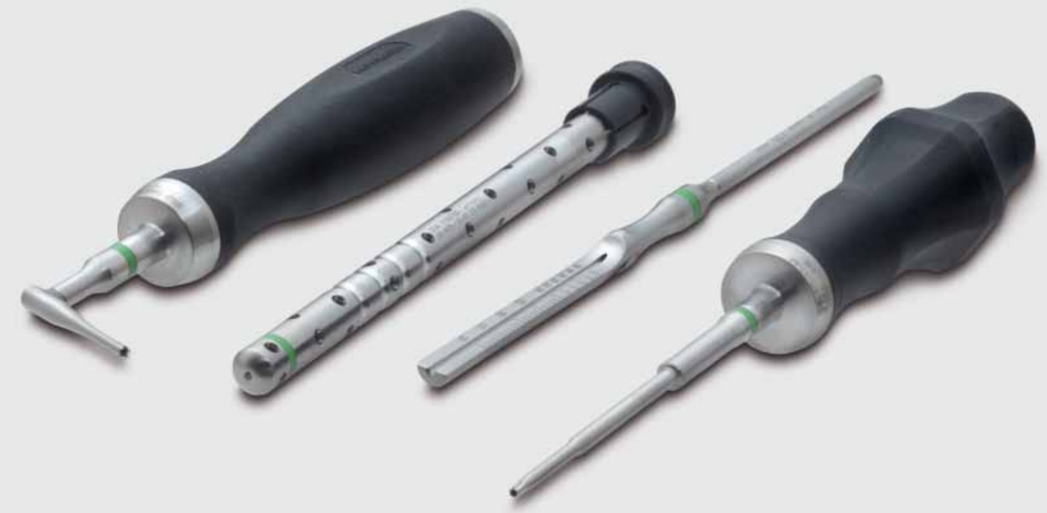

Icon-Erläuterungen

- HBS2 mini
- St Stahl
- Sic Silikon
- T-Drive kanüliert
- 1 unit(s) Verpackungseinheit

HBS2 midi
Optionale Instrumente:

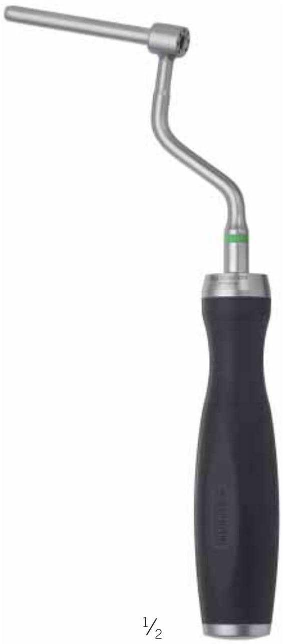

½

26-875-01-07
Gewebeschutzhülse

St Sic 1 unit(s)

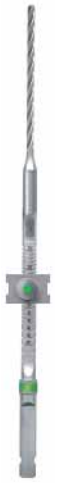

½

26-875-19-09
Spiralbohrer
mit Stopp, kanüliert,
AO-Anschluss
Ø 1,9 / 0,9 mm

St 1 unit(s)

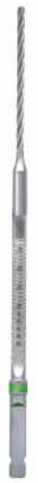

½

26-875-20-09
Spiralbohrer
ohne Stopp, kanüliert
AO-Anschluss
Ø 1,9 / 0,9 mm

St 1 unit(s)

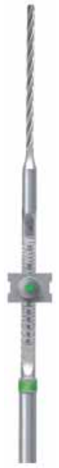

½

26-875-09-09
Spiralbohrer
mit Stopp, kanüliert,
zylindrisch
Ø 1,9 / 0,9 mm

St 1 unit(s)

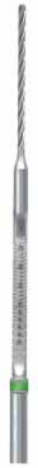

½

26-875-10-09
Spiralbohrer
ohne Stopp, kanüliert,
zylindrisch
Ø 1,9 / 0,9 mm

St 1 unit(s)

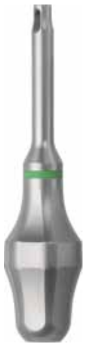

½

26-875-22-07
Schließaufsatz

St 1 unit(s)

Lagerung
HBS2 midi und HBS2 mini

Die HBS2-Lagerung besteht aus verschiedenen Modulen.

Im Instrumenteneinsatz werden sämtliche HBS2-Instrumente, die für eine Operation zwingend notwendig sind, einzeln gelagert.

Optional verfügbare Instrumente wie kanülierte Bohrer mit verschiedenen Ansätzen, die Gewebeschutzhülse oder der Schließaufsatz können im Lagerungskorb ebenfalls einzeln gelagert werden. Darüber hinaus steht eine Freilagerungsfläche zur Verfügung, die individuell genutzt werden kann.

Das Schraubenrondell kann insgesamt 128 Schrauben aufnehmen, jeweils zwei pro Typ und Länge. Entsprechend dem Indikationsspektrum ist die Bestückung individuell vorzunehmen.

Die **HBS2-Set-Nr. 26-800-00-04** beinhaltet neben dem Standardinstrumentarium jeweils einen HBS2-midi-Bohrer und einen HBS2-mini-Bohrer mit Stopp und AO-Anschluss sowie eine Auswahl an Implantaten, die insbesondere auf die Versorgung von Skaphoidfrakturen und Skaphoidpseudarthrosen abgestimmt sind.

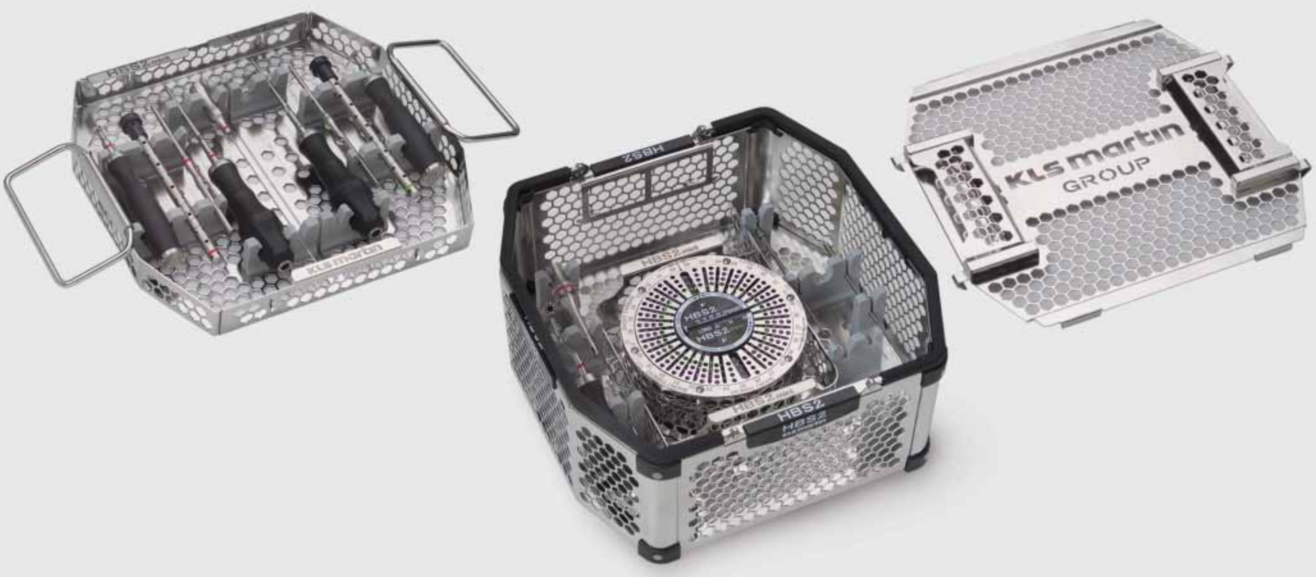

Lagerung		
HBS2 midi	HBS2 mini	
55-910-54-04		Lagerung, bestehend aus:
		Deckel, Instrumenteneinsatz, Lagerungskorb, Schraubenrondell

Standardinstrumentarium		
HBS2 midi	HBS2 mini	
26-850-02-07	26-875-02-07	K-Draht-Führung
26-850-13-07	26-875-13-07	K-Draht-Spender
26-850-00-05	26-875-00-05	Führungsdraht
26-850-06-07	26-875-06-07	Messhülse
26-850-17-07	26-875-17-07	Schraubendreher
26-850-03-07	26-875-03-07	Reinigungsdraht

Optionale Instrumente		
HBS2 midi	HBS2 mini	
26-850-01-07	26-875-01-07	Gewebeschutzhülse
26-850-22-07	26-875-22-07	Schließaufsatz
26-850-09-09	26-875-09-09	Spiralbohrer mit Stopp, kanüliert, zylindrischer Ansatz
26-850-19-09	26-875-19-09	Spiralbohrer mit Stopp, kanüliert, AO-Ansatz
26-850-10-09	26-875-10-09	Spiralbohrer, kanüliert, zylindrischer Ansatz
26-850-20-09	26-875-20-09	Spiralbohrer, kanüliert, AO- Ansatz
26-850-12-07	26-875-12-07	Bohrtiefenanschlag/Stopp

Das KLS-Martin-Leihsystemwesen …
… marLOAN

Warum Leihsysteme?

Insbesondere für Indikationen mit niedriger Inzidenz stellt sich die Frage, ob die Anschaffung eines Implantatsystems sinnvoll ist. Aus der Sicht des Chirurgen fällt die Antwort wahrscheinlich positiv aus, denn in der Ausnahmesituation möchte er auf das bestmögliche System zurückgreifen können. Allerdings ist die Anschaffung aus wirtschaftlicher Sicht kaum vertretbar.

Hier stellt das Leihsystem für die Klinik eine interessante Alternative dar. Wir stellen ein auf Funktion und Vollständigkeit geprüftes System der neuesten Generation leihweise zur Verfügung.

Damit entfällt für die Klinik sowohl die Anschaffung als auch die aufwändige und kostenintensive Instandhaltung und Lagerhaltung des jeweiligen Systems.

Wie funktioniert marLOAN?

Für den deutschsprachigen Raum stehen über 40 Leihsysteme für die Handchirurgie bereit.

Unter der Rufnummer
07461-706-777
oder per E-Mail
mar.loan@klsmartin.com

können Leihsysteme bei Verfügbarkeit bis spätestens 24 Stunden vor OP-Beginn reserviert werden. Dies gilt für Werktage. Gerne können Sie auch den für Sie zuständigen KLS-Martin-Mitarbeiter kontaktieren. Das empfehlen wir insbesondere dann, wenn Sie diesen Service erstmalig in Anspruch nehmen wollen.

Eine kurze Information nach dem Eingriff genügt uns, um die Abholung für Sie zu organisieren.

Berechnet werden in der Regel lediglich pauschal der uns entstehende Aufwand sowie der Verbrauch an Implantaten und Einmalartikeln.

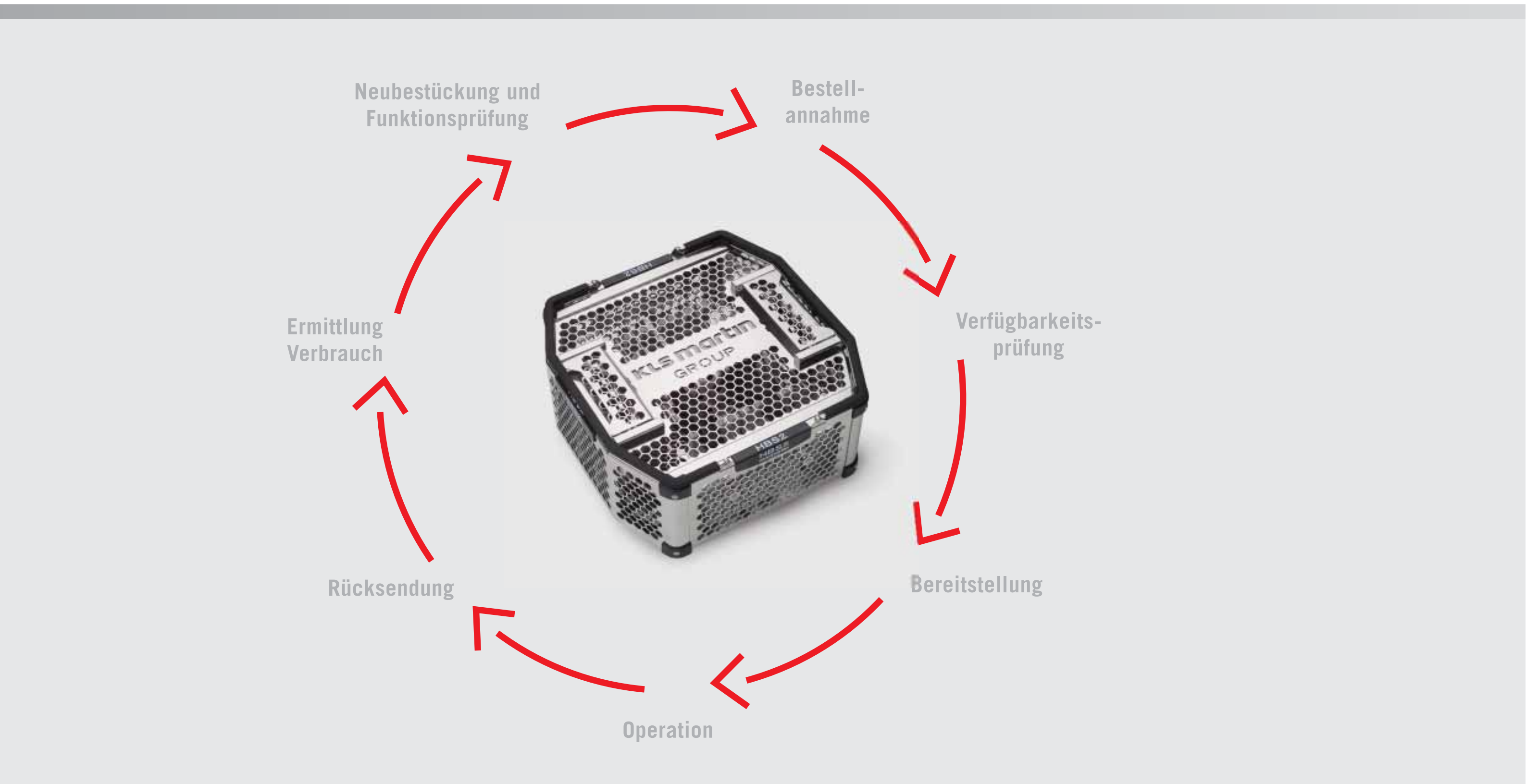

Leihsysteme für den Bereich Handchirurgie

- **Explantationsinstrumentarium**
  Zur sicheren und einfachen Metallentfernung von KLS-Martin-Systemen.
- **FAROS C**
  Multidirektional winkelstabiles Plattensystem für die Radiusrekonstruktion.
- **Flower Plate**
  Multidriektional winkelstabiles Plattensystem für die mediokarpale Teilarthrodese.
- **GENOS MC / mini**
  Interner Distraktor zur Rekonstruktion der Metakarpalia und Brachymetakarpie.
- **Handchirurgische Spezialinstrumente**
  Nach Rücksprache können auch diese leihweise zur Verfügung gestellt werden.
- **HBS / HBS2**
  Headless Bone Screw primär zur Versorgung von Frakturen und Pseudarthrosen kleiner Knochen und Knochenfragmente.
- **IXOS®**
  Multidirektional winkelstabiles Plattensystem zur Versorgung von akuten Radiusfrakturen.
- **MOH / LPS**
  Multidirektional winkelstabiles Plattensystem zur Versorgung von Fingerfrakturen.
- **UHP**
  Ulnakopfprothese bei Störungen des distalen Radioulnargelenkes.
- **URS mini**
  Multidirektional winkelstabiles Plattensystem primär für die ulnare Verkürzungsosteotomie.

KLS Martin Group

Karl Leibinger GmbH & Co. KG
78570 Mühlheim · Germany
Tel. +49 7463 838-0
info@klsmartin.com

KLS Martin GmbH + Co. KG
79224 Umkirch · Germany
Tel. +49 7665 9802-0
info@klsmartin.com

Stuckenbrock Medizintechnik GmbH
78532 Tuttlingen · Germany
Tel. +49 7461 165880
verwaltung@stuckenbrock.de

Rudolf Buck GmbH
78570 Mühlheim · Germany
Tel. +49 74 63 99516-30
info@klsmartin.com

KLS Martin France SARL
68000 Colmar · France
Tel. +33 3 89216601
france@klsmartin.com

Martin Italia S.r.l.
20871 Vimercate (MB) · Italy
Tel. +39 039 6056731
italia@klsmartin.com

Martin Nederland/Marned B.V.
1270 AG Huizen · The Netherlands
Tel. +31 35 5234538
nederland@klsmartin.com

KLS Martin UK Ltd.
Reading RG1 3EU · United Kingdom
Tel. +44 (0) 1189 000 570
uk@klsmartin.com

Nippon Martin K.K.
Osaka 541-0046 · Japan
Tel. +81 6 62289075
nippon@klsmartin.com

KLS Martin L.P.
Jacksonville, Fl 32246 · USA
Tel. +1 904 6417746
usa@klsmartin.com

Gebrüder Martin GmbH & Co. KG
Representative Office
121471 Moscow · Russia
Tel. +7 499 792-76-19
russia@klsmartin.com

Gebrüder Martin GmbH & Co. KG
Representative Office
201203 Shanghai · China
Tel. +86 21 2898 6611
china@klsmartin.com

Gebrüder Martin GmbH & Co. KG
Representative Office
Dubai · United Arab Emirates
Tel. +971 4 454 16 55
middleeast@klsmartin.com

Gebrüder Martin GmbH & Co. KG
Ein Unternehmen der KLS Martin Group
Ludwigstaler Str. 132 · 78532 Tuttlingen · Germany
Postfach 60 · 78501 Tuttlingen · Germany
Tel. +49 7461 706-0 · Fax +49 7461 706-193
info@klsmartin.com · www.klsmartin.com

CE 0297
11.11 · 90-472-01-06 · Printed in Germany

Copyright by Gebrüder Martin GmbH & Co. KG · Alle Rechte vorbehalten · Technische Änderungen vorbehalten
We reserve the right to make alterations · Cambios técnicos reservados · Sous réserve de modifications techniques · Ci riserviamo il diritto di modifiche tecniche
